# Effect of Patient Financial Incentives on Statin Adherence and Lipid Control: A Randomized Clinical Trial

**DOI:** 10.1001/jamanetworkopen.2020.19429

**Published:** 2020-10-09

**Authors:** Iwan Barankay, Peter P. Reese, Mary E. Putt, Louise B. Russell, George Loewenstein, David Pagnotti, Jiali Yan, Jingsan Zhu, Ryan McGilloway, Troyen Brennan, Darra Finnerty, Karen Hoffer, Sakshum Chadha, Kevin G. Volpp

**Affiliations:** 1 Department of Management, The Wharton School, University of Pennsylvania, Philadelphia; 2 Department of Business Economics and Public Policy, The Wharton School, University of Pennsylvania, Philadelphia; 3 Center for Health Incentives and Behavioral Economics, University of Pennsylvania, Philadelphia; 4 Department of Biostatistics, Epidemiology, and Informatics, Perelman School of Medicine, University of Pennsylvania, Philadelphia; 5 Department of Medicine, Perelman School of Medicine, University of Pennsylvania, Philadelphia; 6 Corporal Michael J. Crescenz VA Medical Center, Philadelphia, Pennsylvania; 7 Department of Medicine and Medical Ethics and Health Policy, Perelman School of Medicine, University of Pennsylvania, Philadelphia; 8 Department of Social and Decision Sciences, Carnegie Mellon University, Pittsburgh, Pennsylvania; 9 Department of Health Policy and Management, T. H. Chan School of Public Health, Harvard University, Boston, Massachusetts; 10 CVS Health, Woonsocket, Rhode Island; 11 Rutgers New Jersey Medical School, Newark, New Jersey

## Abstract

**Importance:**

Financial incentives can improve medication adherence and cardiovascular disease risk, but the optimal design to promote sustained adherence after incentives are discontinued is unknown.

**Objective:**

To determine whether 6-month interventions involving different financial incentives to encourage statin adherence reduce low-density lipoprotein cholesterol (LDL-C) levels from baseline to 12 months.

**Design, Setting, and Participants:**

This 4-group, randomized clinical trial was conducted from August 2013 to July 2018 among several large US insurer or employer populations and the University of Pennsylvania Health System. The study population included adults with elevated risk of cardiovascular disease, suboptimal LDL-C control, and evidence of imperfect adherence to statin medication. Data analysis was performed from July 2017 to June 2019.

**Interventions:**

The interventions lasted 6 months during which all participants received daily medication reminders and an electronic pill bottle. Statin adherence was measured by opening the bottle. For participants randomized to the 3 intervention groups, adherence was rewarded with financial incentives. The sweepstakes group involved incentives for daily adherence. In the deadline sweepstakes group, incentives were reduced if participants were adherent only after a reminder. The sweepstakes plus deposit contract group split incentives between daily adherence and a monthly deposit reduced for each day of nonadherence.

**Main Outcomes and Measures:**

The primary outcome was change in LDL-C level from baseline to 12 months.

**Results:**

Among 805 participants randomized (199 in the simple daily sweepstakes group, 204 in the deadline sweepstakes group, 201 in the sweepstakes plus deposit contract group, and 201 in the control group), the mean (SD) age was 58.5 (10.3) years; 519 participants (64.5%) were women, 514 (63.9%) had diabetes, and 273 (33.9%) had cardiovascular disease. The mean (SD) baseline LDL-C level was 143.2 (42.5) mg/dL. Measured adherence at 6 months (defined as the proportion of 180 days with electronic pill bottle opening) in the control group (0.69; 95% CI, 0.66-0.72) was lower than that in the simple sweepstakes group (0.84; 95% CI, 0.81-0.87), the deadline sweepstakes group (0.86; 95% CI, 0.83-0.89), and the sweepstakes plus deposit contract group (0.87; 95% CI, 0.84-0.90) (*P* < .001 for each incentive group vs control). LDL-C levels were measured for 636 participants at 12 months. Mean LDL-C level reductions from baseline to 12 months were 33.6 mg/dL (95% CI, 28.4-38.8 mg/dL) in the control group, 32.4 mg/dL (95% CI, 27.3-37.6 mg/dL) in the sweepstakes group, 33.2 mg/dL (95% CI, 28.1-38.3 mg/dL) in the deadline sweepstakes group, and 36.5 mg/dL (95% CI, 31.3-41.7 mg/dL) in the sweepstakes plus deposit contract group (adjusted *P* > .99 for each incentive group vs control).

**Conclusions and Relevance:**

Compared with the control group, different financial incentives improved measured statin adherence but not LDL-C levels. This result points to the importance of directly measuring health outcomes, rather than simply adherence, in trials aimed at improving health behaviors.

**Trial Registration:**

ClinicalTrials.gov Identifier: NCT01798784

## Key Points

**Question:**

Can daily financial incentives for medication adherence induce lasting habits for statin adherence and sustained reductions in low-density lipoprotein cholesterol (LDL-C) levels even after financial incentives are discontinued?

**Findings:**

In a randomized clinical trial of individuals at elevated risk of cardiovascular disease and with suboptimal cholesterol levels and imperfect adherence, participants in the intervention groups received financial incentives for statin adherence for 6 months. Measured adherence was better among individuals receiving financial incentives, but the change in LDL-C level from baseline to 12 months, the primary outcome, did not differ between intervention and control groups.

**Meaning:**

Measured improvements in adherence after financial incentives did not translate into improved LDL-C levels.

## Teaser

This randomized clinical trial examines whether 6-month interventions involving different financial incentives to encourage statin adherence reduce low-density lipoprotein cholesterol levels from baseline to 12 months.

## Introduction

Heart disease is the leading cause of death in the US.^1^ Among individuals with atherosclerotic cardiovascular disease (ASCVD), statins (HMG-CoA reductase inhibitors) lower the risk of myocardial infarction, with modest cost and manageable adverse effects.^2,3^ For most patients at high risk of ASCVD, statin therapy should be lifelong. However, nonadherence is common,^4,5^ with more than 50% of patients in some cohorts no longer taking statins by 1 year after myocardial infarction.^6^

Financial incentives promote adherence by offering a salient reward for the typically distant effects of statins on cardiovascular disease.^7,8^ Previous studies have focused on efficacy during the active phase of the intervention.^9^ However, which incentive structure promotes durable medication adherence habits after the incentive ends remains a central and largely unanswered question. We conducted a 4-group, randomized clinical trial designed specifically to get closer to an understanding of the optimal structure of financial incentives for statin adherence lasting 6 months and whether these interventions would achieve sustained low-density lipoprotein cholesterol (LDL-C) reductions at 12 months.

The trial design was based on interdisciplinary theories of habit formation and persistence from psychology, management, and economics, and tested 3 distinct hypotheses. First, evidence from psychology suggests that repetition induces automaticity and habits after incentives are removed.^10^ Thus, in the simple sweepstakes group, we offered a daily incentive for medication adherence. Second, management theories suggest that habits arise as a consequence of newly established routines, which we encouraged participants in the deadline sweepstakes group to initiate by paying the full daily financial incentive only if the medication was taken before receiving a daily reminder.^11,12^ Finally, economic theories of habit persistence emphasize that medication adherence reflects past behavior with an effect that diminishes over time.^13,14,15^ To make more salient the extent to which behavior is connected over time, the incentives were focused on helping participants to understand how their present behavior will benefit them in the near future. We operationalized this with loss aversion in the sweepstakes plus deposit contract group, where participants received half of the rewards as a daily incentive and half in the form of a deposit that was reduced each time the participant failed to take the statin; the balance was paid at the end of each month.^16,17,18^ We hypothesized that each of the 3 financial incentive interventions would be more effective than control in achieving LDL-C level reduction at 12 months, 6 months after incentives were discontinued. We also hypothesized that the sweepstakes plus deposit contract would be more effective than either the deadline sweepstakes or standard lottery in achieving sustained reductions in LDL-C levels.

## Methods

### Overview

This study was a randomized clinical trial aimed at improving LDL-C levels. Sweepstakes-based financial incentives were offered to encourage medication adherence over a 6-month intervention period, and the primary outcome, LDL-C level, was evaluated after another 6 months to test for lasting improvements in health outcomes 6 months after the end of incentives. The study was conducted from August 2013 to July 2018. The protocol was approved by the University of Pennsylvania’s institutional review board and the study’s data safety monitoring board. The methods have been described in detail elsewhere,^15^ and the complete trial protocol is shown in Supplement 1. This study follows the Consolidated Standards of Reporting Trials (CONSORT) reporting guideline. Participants provided either written or oral informed consent, as described later.

### Study Populations

Participants were recruited from employees at 4 companies served by a large national pharmacy benefits manager (employers), beneficiaries of a large health plan (insurer), and from the University of Pennsylvania Health System (Penn Medicine).^15^ For potentially eligible patients at Penn Medicine who did not enroll in the study, we obtained institutional review board permission to review deidentified LDL-C records, thus allowing comparison with usual care.

### Eligibility

Initially, eligibility was limited to individuals with diabetes, LDL-C level greater than 130 mg/dL (to convert LDL-C to mmol/L, multiply by 0.0259), and an annual statin medication possession ratio in pharmacy records of less than 80%. We subsequently broadened eligibility criteria to include individuals with a statin prescription who self-reported nonadherence and had either (1) LDL-C level greater than 100 mg/dL and a diagnosis of ASCVD or an American College of Cardiology/American Heart Association Task Force 10-year cardiovascular disease risk score of at least 7.5%, or (2) LDL-C level greater than 190 mg/dL with no other risk factors, or (3) both.

We excluded individuals younger than 18 years, those with contraindications to statin use or adverse effects from statins, such as active or progressive liver disease, and those who did not or could not give consent. For Penn Medicine, individuals enrolled in another behavioral clinical trial or taking proprotein convertase subtilisin–kexin type 9 inhibitors were ineligible.

### Screening, Informed Consent, and Surveys

**Figure 1.**
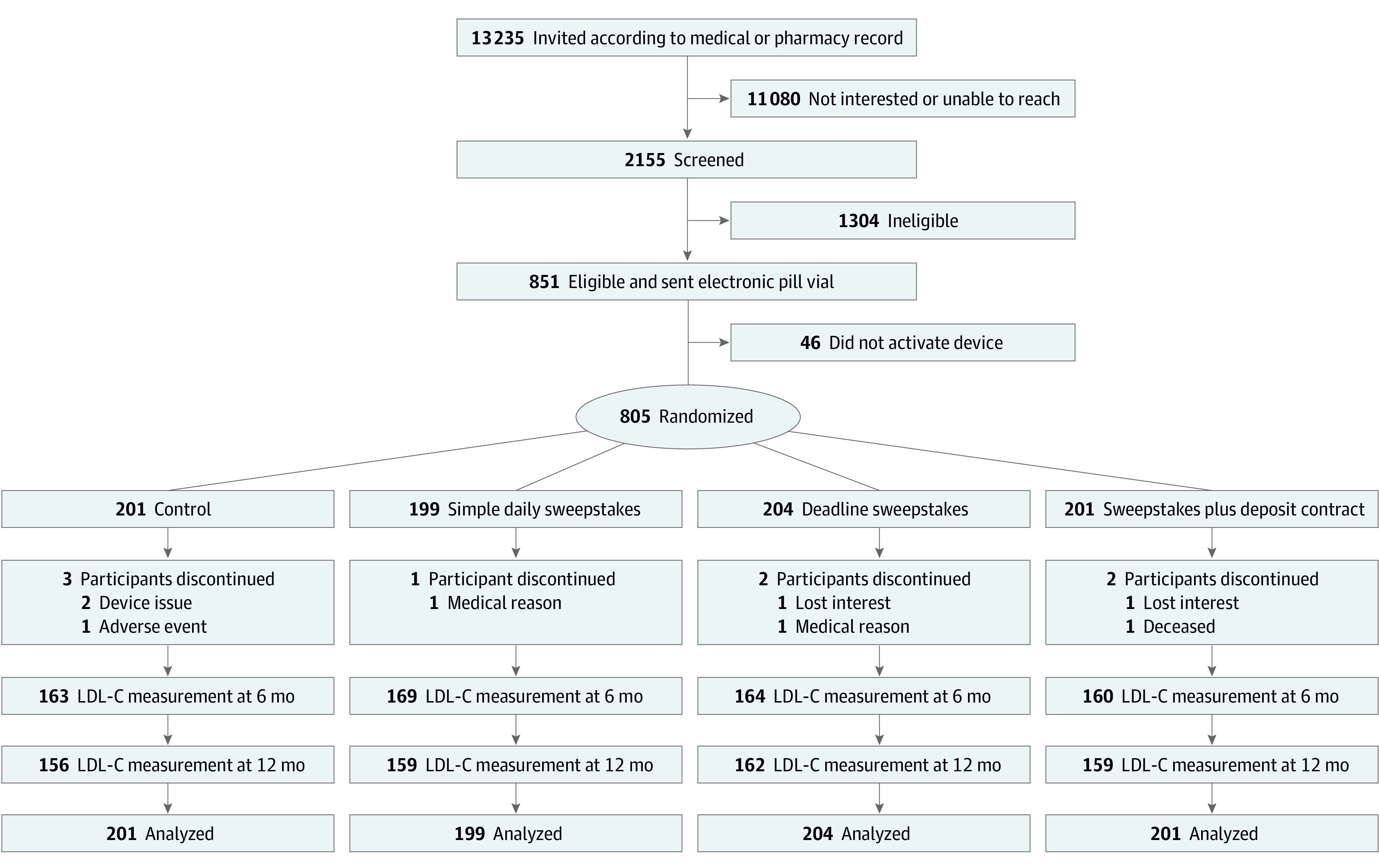
Patient Recruitment and Randomization Flowchart LDL-C indicates low-density lipoprotein cholesterol.

As shown in Figure 1, we invited 13 235 individuals to create accounts on the Way to Health website.^15,19^ Through the website or by telephone with a coordinator, 2155 potential participants provided informed consent and completed a screening survey to confirm eligibility. Invitees were also queried about demographic characteristics; their health characteristics; their knowledge, skill, and confidence in managing their health; risk perception; motivation to engage in behavioral change related to health; and perceived financial constraints.

### Electronic Pill Bottles

A total of 851 participants were eligible, consented, and were mailed electronic pill bottles. When opened, the pill bottle wirelessly transmitted a signal to the Way to Health server. For the purpose of the study, 1 daily bottle opening indicated measured adherence to statin medication that day. In a previous study,^15^ we provided further details on the design and rationale for this trial, as well as a discussion of using electronic pill bottles for adherence measurement.

### Randomization and Blinding

A total of 805 individuals activated their pill bottle and were block-randomized via the Way to Health software platform using computer-generated sequences with equal allocation to each group. Random block sizes were 4, 8, or 12 participants. Participants were aware of their assignment. Investigators and analytical staff were blinded to assignment.

### Interventions

In addition to electronic pill bottles, all participants in all groups received daily reminders to take their statin via text message, automated telephone call, or email per participant preference. Providing reminders to all participants was a deliberate design choice, permitting an analysis that could isolate the effect of financial incentives from messaging because the intervention groups would necessarily receive daily feedback about whether they earned financial incentives. The default reminder time was 10:00 pm, but participants could choose an earlier time before randomization. All participants received up to $200 for completing study milestones, including enrollment and LDL-C measurements at baseline and at 6 and 12 months. The control group received no further intervention. Over a 6-month period, individuals in the 3 intervention groups were eligible for financial incentives based on their measured adherence. We delivered incentives as a sweepstakes to make use of people’s tendency to overestimate small probabilities.^20,21^ On days when patients were nonadherent, we also invoked loss aversion through messages about the reward that they missed. Participants in the intervention groups were informed about their earnings the following day.^15^

Each participant was assigned a 2-digit number. Every day, a random 2-digit number was generated by Way to Health and compared with the participants’ assigned numbers to determine the size of the financial incentives. A fully adherent participant in a financial incentives group could expect to earn, on average, $2.80 per day or $504 total through the 180-day intervention period.

#### Simple Sweepstakes

If both digits matched (1 in 100 probability), an adherent participant received $100. If 1 digit matched (18 in 100 probability), an adherent participant received $10.

#### Deadline Sweepstakes

The incentive was identical to simple sweepstakes if the participant took their statin before their daily reminder. If the participant took their statin after receiving the reminder, the incentive was halved to $50 for a 2-digit match and $5 for a 1-digit match. Because participants chose the time of day for the reminder before randomization and before learning about incentives for adherence, they could not choose the time to maximize their chances of winning in this group.

#### Sweepstakes Plus Deposit Contract

Participants were eligible for daily sweepstake rewards, but with half the expected monetary value of simple sweepstakes. In addition, on the first day of each 30-day month, $45 was deposited into a virtual monthly account. Deductions of $1.50 were made each day that a participant was nonadherent. The amount remaining was paid at the end of the month and the deposit reset to $45 for the next month.

### Outcomes

The primary outcome was change in LDL-C level from baseline to a target date of 12 months, plus or minus 30 days of the target date. Prespecified secondary outcomes included measured adherence over the 6-month intervention period, defined as the proportion of 180 days in which the pill bottle registered an opening, change in LDL-C level from baseline to 6 months, and change in DL-C level between 6 and 12 months. We also examined measured adherence in the final 30 days of the intervention period. For participants recruited from Penn Medicine, we also examined LDL-C values determined from the electronic health record (EHR) collected during routine care up to 24 months after the study (36 months after enrollment) to explore the durability of LDL-C response. Finally, to estimate the change in LDL-C level at 6 months for Penn Medicine patients who were invited to join the study but did not enroll, we collected LDL-C measurements in usual care from the EHR.

### Statistical Analysis

The intention-to-treat analysis specified a linear model with study group and LDL-C level at baseline as covariates and change in LDL-C level from baseline to 12 months as the primary outcome. In stage 1, we tested whether mean change in LDL-C level from baseline to 12 months differed between each intervention group and control, adjusting with a Holm-Bonferroni correction. In stage 2, we planned to compare pairs of interventions deemed different from control in stage 1, adjusting with the Tukey honest significant difference test. Secondarily, in a multivariable model, we adjusted for source population (employers, insurer, or Penn Medicine), sex, education, income, and race. We also tested for differences in change in LDL-C among groups across the intervention period (baseline to 6 months) and after the intervention (6 to 12 months).

The primary analysis accounted for missing data (21% of participants did not have LDL-C measurements at 12 months; balanced across groups) using the fully conditional specification form of multiple imputation.^10,11^ A total of 100 imputations yielded values within range of observed data and 99% relative efficiency. Results were combined using standard Rubin formulae.^12^ Complete case analyses are reported secondarily in Supplement 1.

All hypothesis tests were 2-sided. The primary analysis maintained the familywise type I error rate at 0.05; the remaining analyses were considered exploratory and used a nominal type I error rate of 0.05. Statistical significance was set at *P* < .05.

The study was designed to detect a greater than or equal to 10 mg/dL mean difference in change in LDL-C level between control and any intervention groups, a value considered relevant to clinical reduction in ASCVD events.^15^ On the basis of an earlier trial,^9^ the assumed change in LDL-C SD was 24.5 mg/dL. Assuming 20% loss to follow-up for change in LDL-C, simulations suggested that 200 participants in each group would provide greater than 90% power to detect a 10 mg/dL difference between any intervention and control and at least 80% power to detect an 8.5 mg/dL mean difference between at least 1 pair of intervention groups.^22^ Prespecified subgroup analyses included explorations of any effect of an incentive by source population, sex, race, income, or baseline LDL-C cutoff values (100-129 mg/dL, 130-159 mg/dL, 160-190 mg/dL, and >190 mg/dL).^15^

Measured adherence across the 6-month intervention period was compared across groups using a simple linear model. The incentive groups were pooled, because any effect of a financial incentive on LDL-C level should be mediated primarily through improved adherence. We first assessed the association between measured 6-month adherence and change in LDL-C at 6 or 12 months. For comparability to the model for our primary outcome, we then added baseline LDL-C level as a predictor.

We performed a sensitivity analysis accounting for the effect of the type of electronic pill bottle on the primary outcome. We performed several post hoc analyses. To explore the durability of LDL-C response, LDL-C level from the EHR for up to 24 months after the conclusion of the trial was plotted as a function of time. We also used a deidentified data set from Penn Medicine to examine changes in LDL-C levels measured during usual care in adults who were identified as eligible according to the EHR, but who were not included in the study. LDL-C level was analyzed as a function of time using a mixed-effects model to account for correlations among repeated measurements. This analysis was used to estimate mean LDL-C level change from baseline to 6 and 12 months in the nonenrolled patients.

Simulations for calculating sample size and analysis of the association between measured adherence and were performed in R statistical software version 2.5.1 (R Project for Statistical Computing). All other analyses were conducted using SAS statistical software version 9.4 (SAS Institute). Data analysis was performed from July 2017 to June 2019.

## Results

### Recruitment and Enrollment

Figure 1 displays study enrollment and group assignment. A total of 805 participants were randomized: 199 in the simple daily sweepstakes group, 204 in the deadline sweepstakes group, 201 in the sweepstakes plus deposit contract group, and 201 in the control group. Seven hundred thirty-eight participants (91.7%) were recruited through Penn Medicine.^15^ Participant characteristics were well balanced across study groups (Table 1). The participants had a mean (SD) age of 58.5 (10.3) years, 519 (64.5%) were female, 383 (47.6%) were Black, 429 (53.3%) were married, and 579 (72.1%) had at least some college education. A total of 514 (63.9%) had diabetes, 273 (33.9%) had a diagnosis of ASCVD, and 108 (13.4%) had a screening LDL-C level greater than 190 mg/dL. Change in LDL-C level from baseline to 12 months, the primary outcome, was measured in 636 participants (79.0%) (Figure 1). Multiple imputations were used for missing LDL-C data as the primary analysis. eTable 1 in Supplement 2 shows further description of participants’ baseline characteristics.

**Table 1.** Baseline Participant Characteristics by Intervention Group^a^

Characteristic	Participants, No. (%)				
	Total (N = 805)	Control (n = 201)	Sweepstakes (n = 199)	Deadline sweepstakes (n = 204)	Sweepstakes and contract (n = 201)
Age, mean (SD), y	58.5 (10.3)	57.9 (10.5)	58 (9.2)	59.9 (11.1)	58.1 (10.5)
Female	519 (64.5)	130 (64.7)	130 (65.3)	131 (64.2)	128 (63.7)
Race/ethnicity^b^					
Black	383 (47.6)	89 (44.3)	102 (51.3)	102 (50.2)	90 (44.8)
White	369 (45.9)	98 (48.8)	85 (42.7)	89 (43.8)	97 (48.3)
Other	52 (6.5)	14 (7.0)	12 (6.0)	12 (5.9)	14 (7.0)
Hispanic or Latino^b^	22 (2.8)	8 (4.0)	6 (3.1)	5 (2.5)	3 (1.5)
Education^b^					
High school or less	223 (27.8)	61 (30.3)	63 (32.0)	55 (27.0)	44 (22.0)
Some college	257 (32.0)	64 (31.8)	65 (33.0)	59 (28.9)	69 (34.5)
College degree	322 (40.1)	76 (37.8)	69 (35.0)	90 (44.1)	87 (43.5)
Annual income					
<$50 000	414 (51.4)	105 (52.2)	101 (50.8)	106 (52.0)	102 (50.7)
≥$50 000	380 (47.2)	93 (46.3)	96 (48.2)	95 (46.6)	96 (47.8)
Do not wish to answer	11 (1.4)	3 (1.5)	2 (1.0)	3 (1.5)	3 (1.5)
Marital status					
Single	206 (25.6)	54 (26.9)	54 (27.1)	53 (26.0)	45 (22.4)
Married or unmarried partners	429 (53.3)	113 (56.2)	99 (49.7)	103 (50.5)	114 (56.7)
Divorced or widowed	170 (21.1)	34 (16.9)	46 (23.1)	48 (23.5)	42 (20.9)
Smoke >5 cigarettes on most days^b^	87 (10.8)	18 (9.0)	27 (13.6)	23 (11.3)	19 (9.5)
Recruitment source					
Employer or insurance	67 (8.3)	17 (8.5)	16 (8.0)	18 (8.8)	16 (8.0)
Penn Medicine	738 (91.7)	184 (91.5)	183 (92.0)	186 (91.2)	185 (92.0)
Baseline LDL-C level, mean (SD), mg/dL	143.2 (42.5)	146.6 (45.4)	145.6 (43.7)	140.5 (41.1)	140.1 (39.5)
Diagnosed with diabetes	514 (63.9)	120 (59.7)	136 (68.3)	127 (62.3)	131 (65.2)
Diagnosed with ASCVD	273 (33.9)	77 (38.3)	56 (28.1)	74 (36.3)	66 (32.8)
Screening LDL-C level >190 mg/dL	108 (13.4)	30 (14.9)	27 (13.6)	24 (11.8)	27 (13.4)
10-y ASCVD risk of at least 7.5%^b^^,^^c^	12 (1.5)	4 (2.0)	2 (1.0)	5 (2.5)	1 (0.5)
Pill bottle model^d^					
1	507 (63.1)	134 (67.1)	125 (63.1)	123 (60.1)	125 (62.1)
2	184 (23.1)	44 (22.1)	45 (23.1)	48 (24.1)	47 (23.1)
3	63 (8.1)	17 (8.1)	16 (8.1)	18 (9.1)	12 (6.1)
Multiple models	51 (6.1)	6 (3.1)	13 (7.1)	15 (7.1)	17 (8.1)

SI conversion factor: To convert LDL-C to mmol/L, multiply by 0.0259.

Abbreviations: ASCVD, atherosclerotic cardiovascular disease; LDL-C, low-density lipoprotein cholesterol.

^a^ No differences across columns were statistically significant.

^b^ Contains missing data. Percentages will not sum to 100.

^c^ Assessed only if participant was not eligible according to diagnoses of diabetes or ASCVD or LDL-C level greater than 190 mg/dL.

^d^ See Putt et al^15^ for detailed description of pill bottle designs and Supplement 1 for sensitivity analysis.

Over the 6-month intervention period, the mean per-patient incentive payments were $414.84 for the simple sweepstakes group, $384.24 for the deadline sweepstakes group, and $465.42 in the sweepstakes plus deposit contract group. Measured adherence at 6 months (ie, the proportion of 180 days with electronic pill bottle opening) in the control group (0.69; 95% CI, 0.66-0.72) was lower than that in the simple sweepstakes group (0.84; 95% CI, 0.81-0.87), the deadline sweepstakes group (0.86; 95% CI, 0.83-0.89), and the sweepstakes plus deposit contract group (0.87; 95% CI, 0.84-0.90) (*P* < .001 for each incentive group vs the control group) (Table 2) (see also eTable 2 in Supplement 2 for subgroup analyses and eTable 3 in Supplement 2 for analysis of data from final 30 days of the intervention phase).

**Table 2.** Change in LDL-C Level and Measured Adherence by Intervention Group

Outcome, time, and metric	Trial group, mean (95% CI)			
	Control	Simple sweepstakes	Deadline sweepstakes	Sweepstakes and deposit
Change in LDL-C level, mg/dL^a^				
0-12 mo (primary outcome)^b^				
Change in LDL-C	−33.6 (−38.8 to −28.4)	−32.4 (−37.6 to −27.3)	−33.2 (−38.3 to −28.1)	−36.5 (−41.7 to −31.3)
Difference from control		1.2 (−6.1 to 8.4)	0.5 (−6.9 to 7.8)	−2.9 (−10.1 to 4.4)
* P* value^c^		>.99	>.99	>.99
0-6 mo^b^				
Change in LDL-C	−37.2 (−42.1 to −32.4)	−35.5 (−40.2 to −30.7)	−33.6 (−38.4 to −28.9)	−36.5 (−41.4 to −31.7)
Difference from control		1.8 (−5.0 to 8.6)	3.6 (−3.2 to 10.5)	0.7 (−6.1 to 7.5)
* P* value		.61	.30	.84
6-12 mo^b^				
Change in LDL-C	3.6 (−2.4 to 9.6)	3.0 (−3.0 to 9.0)	0.5 (−5.4 to 6.3)	0.0 (−5.9 to 6.0)
Difference from control		−0.6 (−9.0 to 7.8)	−3.2 (−11.5 to 5.2)	−3.6 (−12.0 to 4.8)
* P* value		.89	.46	.40
Measured adherence^d^				
0-6 mo				
Measured adherence	0.69 (0.66 to 0.72)	0.84 (0.81 to 0.87)	0.86 (0.83 to 0.89)	0.87 (0.84 to 0.90)
Difference from control		0.15 (0.11 to 0.19)	0.17 (0.13 to 0.21)	0.19 (0.15 to 0.23)
* P* value		<.001	<.001	<.001
Final 30 d				
Measured adherence	0.59 (0.55 to 0.63)	0.77 (0.73 to 0.81)	0.83 (0.79 to 0.87)	0.84 (0.80 to 0.88)
Difference from control		0.18 (0.12 to 0.24)	0.24 (0.18 to 0.30)	0.25 (0.19 to 0.31)
* P* value		<.001	<.001	<.001

SI conversion factor: To convert LDL-C to mmol/L, multiply by 0.0259.

Abbreviation: LDL-C, low-density lipoprotein cholesterol.

^a^ Missing follow-up LDL-C measurements were addressed using multiple imputation; change in LDL-C is modeled as a function of study group with centered baseline LDL-C included as a covariate.

^b^ These values were calculated as LDL-C at the later time point minus LDL-C at the earlier time point; negative values indicated a reduction in LDL-C at the later time point.

^c^ For pairwise comparisons of change in LDL-C from baseline (0) to 12 months, Holm-Bonferroni adjusted *P* values are reported.

^d^ Calculated as the proportion of first 180 days with recorded electronic pill bottle openings.

**Figure 2.**
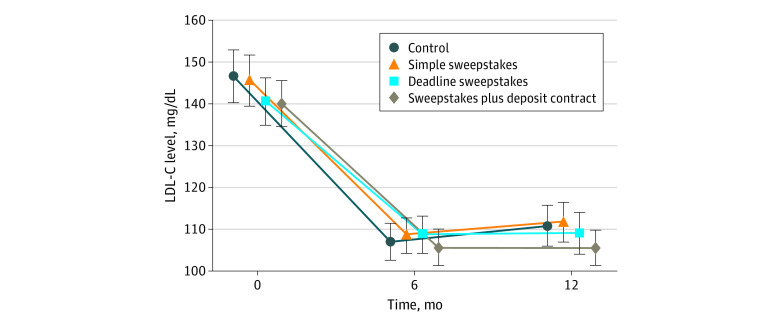
Low-Density Lipoprotein Cholesterol (LDL-C) Levels Over Time by Intervention Group Data points show mean values, and error bars show 95% CIs. To convert LDL-C to mmol/L, multiply by 0.0259.

Figure 2 shows baseline LDL-C level and changes in LDL-C level over time among participants with LDL-C measurements. At baseline, the mean (SD) LDL-C level was 143.2 (42.5) mg/dL. At 12 months, mean LDL-C reductions from baseline were substantial and similar across all groups: 33.6 mg/dL (95% CI, 28.4-38.8 mg/dL) in the control group, 32.4 mg/dL (95% CI, 27.3-37.6 mg/dL) in the simple sweepstakes group, 33.2 mg/dL (95% CI, 28.1-38.3 mg/dL) in the deadline sweepstakes group, and 36.5 mg/dL (95% CI, 31.3-41.7 mg/dL) in the sweepstakes plus deposit contract group (Holm-Bonferroni adjusted *P* > .99 for each incentive group vs the control group) (Table 2). Because no incentive group differed from the control group, no further pairwise comparisons were made. Mean change in LDL-C level from baseline to 6 months was also similar among intervention and control groups, as was mean change in LDL-C level from 6 to 12 months. Among prespecified subgroups, there were no notable differences in mean change in LDL-C level across groups for any pair of time points. A complete case analysis also demonstrated no significant differences in mean change in LDL-C level across groups (eTable 4 in Supplement 2).

To examine the durability of results in post hoc analyses, we considered 399 of the Penn Medicine trial participants (54.1% of 738 enrolled) who had both an on-study LDL-C measurement at 12 months and at least 1 off-study LDL-C measurement in the EHR between 12 and 36 months. On average, these study participants maintained their LDL-C levels over the poststudy period (eFigure 1 and eTable 5 in Supplement 2).

The large decrease in LDL-C levels among controls was unexpected. As a second post hoc analysis, we sought to determine whether nonenrolled patients also experienced a large decrease. The Penn Medicine EHR identified 4404 eligible patients, from whom we had enrolled 805 study participants.^15^ Nonenrolled patients had slightly lower LDL-C levels at baseline and were more likely to be male and White (see eTable 6 in Supplement 2 for characteristics of nonenrolled patients). Among the nonenrolled patients, 2490 (67.9%) had at least 1 LDL-C measurement in the EHR after the baseline LDL-C measurement. Their estimated mean reduction in LDL-C level was 31.0 mg/dL (95% CI, 29.5-32.5 mg/dL) at 6 months and 27.8 mg/dL (95% CI, 26.0-29.6 mg/dL) at 12 months (eTable 6, eTable 7, and eFigure 2 in Supplement 2), comparable to the changes observed in the control group, suggesting that the large decrease in LDL level was not induced by study participation.

**Figure 3.**
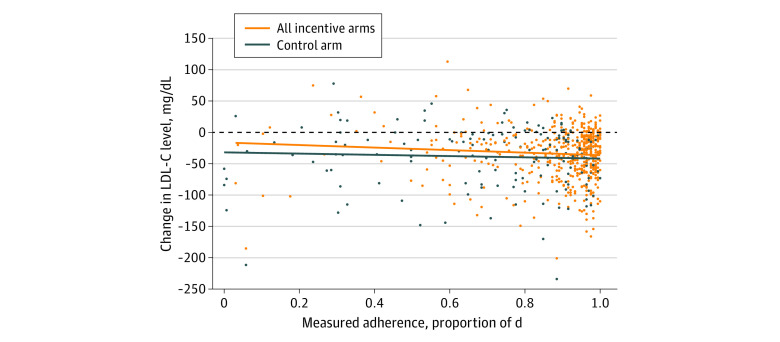
Change in Low-Density Lipoprotein Cholesterol (LDL-C) Levels for the Control and Combined Incentive Groups Change in LDL-C is shown as a function of measured adherence (proportion of days with electronic pill bottle openings) over the 6-month intervention period. The dots show observed data, and the lines indicate predicted values with 95% CIs for separate linear regressions of change in LDL-C on measured adherence alone. To convert LDL-C to mmol/L, multiply by 0.0259.

An exploration of the relationship between measured adherence and change in LDL-C yielded additional findings. Figure 3 plots the data for measured adherence over the 6-month intervention (proportion of 180 days with electronic pill bottle opening) vs change in LDL-C level at 6 months, with separate regression lines for the control and the combined incentive groups. Figure 3 shows that the association of measured adherence with changes in LDL-C differed for the intervention vs control participants. For any given level of measured adherence, the control group demonstrated larger reduction in change in LDL-C level than the incentive group (mean difference, 6.2 mg/dL; 95% CI, 0.4-12.0 mg/dL; *P* = .04). Further analyses appear in eTable 8 and eFigure 3 in Supplement 2.

## Discussion

In this randomized clinical trial testing the effects of carefully designed rewards to promote habit formation in statin adherence, financial incentives improved measured adherence but did not improve LDL-C levels compared with a control group. We used insights from economic theory, management, and psychology to test 3 behavioral incentive interventions. The first group received simple daily incentives to instill automaticity. To produce a self-initiated health routine, participants in a second group received a full incentive for taking the statin before a daily reminder and a reduced incentive if the statin was ingested after the reminder. The third combined a hybrid sweepstakes plus deposit contract, emphasizing loss aversion. We found that the simple financial incentives led to comparable effects on measured adherence and LDL-C level changes as the more complex interventions. In designing the trial, we hypothesized that statin adherence would be a context in which the instant gratification provided by salient financial incentives could be impactful because statins convey no immediate, perceptible benefits to most patients. Instead, the health benefits are distant and diffused in the future. Given this strong theoretical basis for using financial incentives to promote medication adherence, careful consideration should be given as to why none of the incentives improved LDL-C level over the control group. The results give rise to important considerations in the pressing research agenda to induce positive and lasting changes in health behavior using time-limited interventions.

First, we draw attention to the fact that participants were at high risk for ASCVD events, were already prescribed statins, and were recruited after a clinical encounter showing elevated LDL-C levels. Most were receiving care within an academic health system. Notably, they had high adherence during the trial, as measured by opening of electronic pill bottles, across all study groups. Most high-risk individuals presenting with high LDL-C levels in a health system would be expected to receive usual care interventions and counseling to lower their cholesterol. Both control participants and eligible nonenrolled patients achieved clinically meaningful reductions in LDL-C levels that were comparable to the reductions in the intervention groups at the conclusion of the 12-month study. Given that eligibility relied on suboptimal LDL-C levels recently measured in usual care, clinicians may already have been active in intensifying the dose or the type of statin and encouraging healthful behavior concurrent with the trial. Mean reversion can also explain some of the LDL-C improvement. Taken together, these observations suggest that although financial incentives did not reduce LDL-C levels in this population of patients, financial incentives could still be a useful intervention for patients with lower degrees of health engagement who do not use or have access to primary care. This is especially possible because the effect of statins on LDL-C reduction at the start of medication regimens is substantial and well-documented.^23,24,25^

Second, our data present the possibility that financial incentives improved adherence, but that this better statin adherence did not lead to greater improvements in LDL-C levels vs control. To explore this idea, we used values from the first 6 months of adherence data when the interventions were in effect. Over that period, for the same level of measured adherence, the controls tended to have better change in LDL-C level than those in the incentive groups. This discordance between measured adherence and LDL-C level between intervention and control patients could be explained if financial incentives led participants to neglect other health-related behaviors that affect cholesterol, such as diet and exercise. Another possibility is that control participants, who did not receive incentives for adherence, took their medication from other pill bottles, leading to a potential upward bias in the estimated difference in adherence across groups.

Third, given the high adherence even in the control group, the marginal adherence gain among intervention groups may have generated only a small effect on LDL-C level. Although adherence was a secondary outcome here, and electronic pill bottle data are an improvement over self-reported adherence data or medication refill rates, our study reinforces the imperative to go beyond adherence and instead focus, as was done in this trial, on the health outcomes or validated surrogate outcomes of primary interest. This is particularly salient because ASCVD is the primary cause of death in the US, and a major public health priority is to lower its incidence by better managing the associated risk factors.

### Limitations

In addition to the challenges of accurately measuring medication adherence, we acknowledge other limitations of this study. Approximately 21% of participants did not have a 12-month LDL-C measurement, despite robust efforts to encourage participants to complete this laboratory test. To address bias, we followed a prespecified analysis plan of multiple imputation to account for missingness. We also acknowledge that our findings apply to long-term statin users with incomplete adherence who were principally recruited from a single health system. Individuals who are newly prescribed a statin, those without consistent relationships with primary care or specialty physicians, or those who do not access routine care might respond differently to interventions similar to those used here.

## Conclusions

In conclusion, this large randomized clinical trial showed that theory-driven financial incentives for statin adherence may have improved adherence but did not result in better LDL-C levels compared with the control group. In light of abundant evidence from other settings that adherence to statins and other effective medicines is often poor, investigators must continue to concentrate on developing effective strategies to improve adherence. Our findings suggest that these future interventions might focus in particular on patients with low levels of health engagement or who do not have consistent relationships with their physicians. Our results also underscore the importance of directly measuring and targeting health outcomes, rather than only adherence, in the study of financial incentives.

## Notes

Supplement 1. **Trial Protocol**

Supplement 2. **eTable 1.** Baseline Characteristics by Arm **eTable 2. **Measured Adherence 0-6 Months (Means, 95% CI) by Arm **eTable 3. **Measured Adherence During Final 30 Days of Intervention (Means, 95% CI) by Arm **eTable 4. **Change in LDL-C From Baseline to 12 Months (Means, 95% CI) by Arm **eTable 5. **Penn Medicine: Baseline Characteristics of Participants With and Without a Post-Study LDL-C Measurement **eFigure 1. **Penn Medicine: Change in LDL-C Post-Study With Loess Smoothed Curves **eTable 6. **Penn Medicine: Baseline Characteristics of Eligible Nonenrolled Patients Compared to Enrolled Participants in Control Group **eFigure 2. **Penn Medicine: Nonenrolled Individuals: Change in LDL-C From Baseline With Loess Smoothing Curves **eTable 7.** Penn Medicine: Mean (95% CI) Change From Baseline in LDL-C at 6 and 12 Months for Participants Enrolled to the Control Arm and for Nonenrolled Individuals **eTable 8. **Regression Models for the Reduction in Δ*_LDL_* From Baseline to Either 6- or 12-Months by 6-Month Measured Adherence (Proportion of Days With Electronic Pill Bottle Opened Over 180 Days) **eFigure 3. **Change in LDL-C From Baseline to 6 Months in Individuals With Measured Adherence of Zero During Either the Entire 6 Months (n=2) or the Final 30 Days of the Intervention (n=23 for Control and n=16 for Incentives)

Supplement 3. **Data Sharing Statement**
