# Supplementary material for: Effect of Patient Financial Incentives on Statin Adherence and Lipid Control: A Randomized Clinical Trial
Source: JAMA Netw Open. 2020 Oct 9;3(10):e2019429. doi: 10.1001/jamanetworkopen.2020.19429 (PMC7547367; doi:10.1001/jamanetworkopen.2020.19429)
Supplement: Supplement 1. — Trial Protocol [file jamanetwopen-e2019429-s001.pdf]

The Habit Formation Trial
Statistical Analysis Plan

Table of Contents

1 Statistical Analysis Plan: Habit Formation 1

2 Original Statistical Analysis Plan 1

2.1 Descriptive analyses 1

2.2 Primary analysis 2

2.3 Secondary analyses 2

3 Additional Analyses Specified Prior to Completion of Trial 3

4 Exploratory Analyses Specified after Completion of the Trial 4

4.1 Measured adherence and ΔLDL 4

4.2 Post-study changes in LDL-c for enrolled participants 4

4.3 ΔLDL from baseline to 6 months in non-enrolled subjects. 4

1 Statistical Analysis Plan: Habit Formation

This document contains the original statistical analysis plans as well as modifications that were made both while the study was ongoing and after completion of the study.

2 Original Statistical Analysis Plan

2.1 Descriptive analyses

1. Descriptive analyses of baseline characteristics of the sample. (Table 1)

   Describe the two populations (Insurers & Employers) and Health System and the sample, stratified by arm (include numbers (%) missing for each variable)

   a. Age
   b. Sex
   c. Race/ethnicity
   d. Income/household size/financial situation/marital status
   e. Education
   f. Self-reported medication adherence
   g. Self-reported measures of health condition

h. Patient Activation Measure (PAM)

2. Descriptive analyses of LDL-c
    a. Baseline LDL-c (mean, median, IQR, SD, 95% CI on mean), show boxplots of this variable stratified by arm. Assess normality graphically.
    b. Repeat for 6 months and 12 months in cross-sectional fashion
    c. Longitudinal (spaghetti) plots of LDL-c over time stratified by arm (baseline, 6 months, 12 months)
    d. Means by arm over visit (baseline, 6 months, 12 months)
    e. Spaghetti plot of individual LDL & change in LDL cholesterol, Δ_{LDL}, Δ_{LDL} over time. Specify time as a visit, and also as a day post randomization. Plot $\Delta_{LDL}$from baseline to 6 months and baseline to 12 months as a function of baseline LDL-c, by arm
    f. Correlation of LDL-c at months 6 and 12 with baseline LDL, by arm
    g. Rate of missing LDL-c by arm at 6 months and 12 months
    h. Waterfall plots of baseline LDL-c by arm and Δ_{LDL} by arm.

2.2 Primary analysis

Linear regression model of Δ_{LDL}, from baseline to month 12 as a function of study arm with baseline LDL included as a covariate and stratified by population (Employer/Insurer and UPHS)

a. Use multiple imputation to adjust for missing data, and compare to complete case analysis. Report both but specify multiple imputation as the primary analysis.
    i. Description of characteristics of subjects who are missing 12-month data (with +/- 30 days window)
    ii. Imputation model: includes arm assignment, and all of the baseline demographics described above.
b. Hypothesis testing as described in the protocol. Maintain family-wise type I error rate of 0.05.
    i. Step 1: Compare each arm to control using Holm-Bonferroni adjustment;
    ii. Step 2: Pairwise comparison of arms found different to control in Step 1 to each other using Tukey HSD adjustment.

2.3 Secondary analyses

Secondary analyses (Δ_{LDL}).

a. Repeat primary analysis using linear regression model of Δ_{LDL} from baseline to 6 months and from 6 to 12 months.
    i. Adjust for covariates including race, gender, income, education
b. Subgroup analyses: Include treatment arm in each of the models and do separate analyses of Δ_{LDL}. Consider the following subgroups.
    i. Gender
    ii. Race
    iii. Income: stratify at <$50,00 >$50,000 with No-Answer as a separate group

    iv. LDL-c at baseline: Use the cutoffs previously used to guide clinical judgement: (LDL 100-129, 130-159, 160-190, >190 mg/l)

c. Describe measured adherence via pill bottle openings for the entire 6 months of the study by arm, gender, income, baseline LDL

d. Estimate the association between measured adherence across all 6 months of the intervention and Δ_{LDL} from baseline to 6 months and baseline to 12 months.

3 Additional Analyses Specified Prior to Completion of Trial

We will explore possible impacts of the period with higher rates of device failure. Our study is not powered for hypothesis tests of these effects. These analyses will be exploratory.

It is not feasible to accurately determine whether the devices of each individual participant were defective. We will ask whether exposure to a period of time with high device failure rates was associated with change in LDL-c and the effect of the intervention on the change in LDL-c differed during this period. We identified a period of time when the devices failed at higher rates and when we began replacing devices at higher rates, specifically December 1 2015 to May 31 2016. We then determined whether each subject was potentially exposed to a faulty device based on their calendar time of enrollment.

1. Each participant will be assigned to a stratum based on whether they were enrolled in the study during the 'high-failure' calendar-period of time. For each stratum (subjects participating/not participating during the faulty device period) and for each arm, we will create estimates and 95% CI of the mean Δ_{LDL}varied by arm and by stratum. This will estimate an alteration in effect size (difference in LDL-c reduction between arms) due to exposure to the 'high-failure' window of time.
2. The idea here is identical to (1) except that rather than coding participation during a faulty device period as a binary variable we will create a quantitative variable defining the proportion of time in the window with properly functioning devices. For most subjects, this variable will be 1, but for subjects with say 2 months in the calendar window of faulty devices, this would be 60/360=.167. We will also create a variable, Z, indicating whether a subject experienced at least one device swap. As in (1) we will estimate an effect on Δ_{LDL}varied of exposure to the high-failure period and the alteration in effect size due to increasing exposure to the 'high-failure' window of time.
3. We will compare the probability of completing the study (lab visit at 12 months) by whether the subject spent time in the 'high-failure' window.
4. Lastly, we used 3 different devices in this study. Of interest is how Δ_{LDL}varied as a function of device type, and whether there was an interaction between device and treatment arm.

4 Exploratory Analyses Specified after Completion of the Trial

4.1 Measured adherence and Δ_{LDL}

1. In addition to measured adherence across 6 months of the intervention, consider measured adherence during the final 30 days of the intervention. Compared to the entire 6 month measured adherence, we hypothesize that measured adherence during the final 30 days of the intervention may be more closely related to Δ_{LDL} from baseline to 6 months.
2. Explore how the association between measured adherence (final 30 days or 6 months) and Δ_{LDL} may vary as a function of intervention.

4.2 Post-study changes in LDL-c for enrolled participants

This analysis will explore how reductions in LDL-c observed in the study are maintained post-study. Using usual care measurements of LDL-c in the electronic health record (EHR), we will determine Δ_{LDL} for participants post-study and plot these data as a function of time since enrollment and intervention arm. This analysis is restricted to participants from Penn Medicine. Demographic characteristics of participants with post-study measurements will be compared to those without post-study measurements.

4.3 Δ_{LDL} from baseline to 6 months in non-enrolled subjects.

Using usual care measurements of LDL-c in the electronic health record (EHR), we will determine Δ_{LDL} for participants who were offered the option of participating in the study but who did not enroll. This analysis is restricted to participants and potential participants from Penn Medicine. Demographic characteristics of enrolled will be compared to non-enrolled participants with post-study measurements. Non-enrolled subjects have baseline measurements but do not have lab measurements at specific timepoints. We will model Δ_{LDL} as a function of time using a mixed effects model with baseline LDL, age, gender and race as covariates in the model. We will use the model to estimate the mean Δ_{LDL} at 6 and 12 months as a function of different demographic groups.

Modification

Basic Info

Confirmation Number:	**cigdccgd**
Protocol Number:	**816003**
Created By:	**NORTON, LAURA A**
Principal Investigator:	**BARANKAY, IWAN**
Protocol Title:	**Testing Behavioral Economic Interventions to Improve Statin Use and Reduce CVD Risk**
Short Title:	**Habit formation for adherence to statin use and LDL reduction**
Protocol Description:	**In a 4-arm, Randomized Control Trial among members of CVS Caremark and Penn Medicine patients with suboptimal cholesterol control who are at high risk for CVD, we propose to test the effectiveness of different behavioral economic techniques in inducing habit formation for adherence to statin use and sustained reductions in LDL cholesterol after financial incentives are discontinued. Primary outcome is changes in LDL from enrollment to 12 months (6 months after cessation of financial incentives).**
Submission Type:	**Biomedical Research**
Application Type:	**EXPEDITED Category 2**

PennERA Protocol Status

Approved (No CR)

Resubmission*
No

Are you submitting a Modification to this protocol?*

Yes

Current Status of Study

Study Status

Closed to subject enrollment (remains active)

If study is currently in progress, please enter the following

Number of subjects enrolled at Penn since the study was initiated

805

Actual enrollment at participating centers

0

If study is closed to further enrollment, please enter the following

Number of subjects in therapy or intervention

0

Number of subjects in long-term follow-up only

0

IRB Determination

If the change represents more than minimal risk to subjects, it must be reviewed and approved by the IRB at a convened meeting. For a modification to be considered more than minimal risk, the proposed change would increase the risk of discomfort or decrease benefit. The IRB must review and approve the proposed change at a convened meeting before the change can be implemented unless the change is necessary to eliminate an immediate hazard to the research participants. In the case of a change implemented to eliminate an immediate hazard to participants, the IRB will review the change to determine that it is consistent with ensuring the participant′s continued welfare. Examples: Convened Board Increase in target enrollment for investigator initiated research or potential Phase I research Expanding inclusion or removing exclusion criteria where the new population may be at increased risk Revised risk information with active participants Minor risk revisions that may affect a subject′s willingness to continue to participate Expedited Review Increase in target enrollment at Penn where overall enrollment target is not exceeded or potentially sponsored research Expanding inclusion or removing exclusion where the new population has the same expected risk as the previous, based on similarities of condition Revised risk information with subjects in long-term follow-up Minor risk revisions with no subjects enrolled to date

Expedited Review

Modification Summary

Please describe any required modification to the protocol. If you are using this form to submit an exception or report a deviation, enter 'N/A' in the box below.

Dear IRB Administrator, On behalf of Dr. Iwan Barankay, we are submitting a modification to add Yoonhee Ha to our IRB protocol. Yoonhee is a MD-PhD student, working under Dr. Reese a co-principle investigator on this study, who will support our current data analysts with secondary and exploratory analyses. We are submitting the following CITI Training certification document for your review: Citi Training Refresher Course_Yoonhee Ha.Pdf We thank you for your continued support of this study. Please be in contact with the study team if you have any additional questions or concerns. You can reach us by phone at (215) 746-8437 khoffer@pennmedicine.upenn.edu

Risk / Benefit

Does this amendment alter the Risk/Benefit profile of the study?

No

Change in Consent

Has there been a change in the consent documents?

No

If YES, please choose from the options below regarding re-consenting

Deviations

Are you reporting a deviation to this protocol?*

No

Exceptions

Are you reporting an exception to this protocol?*

No

Protocol Details

Resubmission*
Yes

Hospital Sites
Will any research activities and/or services be conducted at a Penn Medicine affiliated hospital site?
No

Study Personnel

Principal Investigator

Name:	**BARANKAY, IWAN**
Dept / School / Div:	**706 - Management**
Campus Address Mail Code	**6370**
Address:	**WHAT MGMT-2000 S/D<br>3620 LOCUST WALK**
City State Zip:	**PHILADELPHIA PA 19104-6370**
Phone:	**215-898-6372**
Fax:	**-**
Pager:	
Email:	**barankay@wharton.upenn.edu**
HS Training Completed:	**Yes**
Training Expiration Date:	**08/24/2012**
Name of course completed :	**CITI Protection of Human Subjects Research Training - ORA**

Study Contacts

Name:	**PAGNOTTI, DAVID R**
Dept / School / Div:	**10599 - ME-Division of Health Policy**
Campus Address Mail Code	**6021**
Address:	**BLOCKLEY HALL<br>423 GUARDIAN DR**
City State Zip:	**PHILADELPHIA PA 19104-6021**
Phone:	**215-573-2770**
Fax:	**-**
Pager:	
Email:	**davidrp@pennmedicine.upenn.edu**
HS Training Completed:	**Yes**
Training Expiration Date:	**09/13/2018**
Name of course completed :	**CITI Protection of Human Subjects Research Training - ORA**

Name:	**HOFFER, KAREN L**
Dept / School / Div:	**4607 - CC-Cancer Center**
Campus Address Mail Code	**3319**
Address:	**3401 MARKET ST**<br>**3401 MARKET ST**
City State Zip:	**PHILADELPHIA PA 19104-3319**
Phone:	**215-898-6086**
Fax:	**-**
Pager:	
Email:	**khoffer@mail.med.upenn.edu**
HS Training Completed:	**Yes**
Training Expiration Date:	**04/28/2019**
Name of course completed :	**CITI Protection of Human Subjects Research Training - ORA**

Other Investigator

Name:	**VOLPP, KEVIN G**
Dept / School / Div:	**10599 - ME-Division of Health Policy**
Campus Address Mail Code	**6021**
Address:	**BLOCKLEY HALL**<br>**423 GUARDIAN DR**
City State Zip:	**PHILADELPHIA PA 19104-6021**
Phone:	**215-573-0270**
Fax:	**-**
Pager:	
Email:	**volpp70@wharton.upenn.edu**
HS Training Completed:	**Yes**
Training Expiration Date:	**10/27/2012**
Name of course completed :	**CITI Protection of Human Subjects Research Training - ORA**

Responsible Org (Department/School/Division):

4239 - DM-General Internal Medicine

Key Study Personnel

Name:	**PAGNOTTI, DAVID R**
Department/School/Division:	**ME-Division of Health Policy**
HS Training Completed:	**No**
Training Expiration Date:	
Name of course completed:	

Name:	**YAN, JIALI**
Department/School/Division:	**DM-General Internal Medicine**
HS Training Completed:	**Yes**
Training Expiration Date:	**06/08/2020**
Name of course completed:	**CITI Protection of Human Subjects Research Training - ORA**

Name:	**MUSSELL, ADAM S**
Department/School/Division:	**DM-Renal-Electrolyte and Hypertension**
HS Training Completed:	**Yes**
Training Expiration Date:	**04/16/2016**
Name of course completed:	**CITI Protection of Human Subjects Research Training - ORA**

Name:	**KOPINSKY, MICHAEL**
Department/School/Division:	**Health System**
HS Training Completed:	**Yes**
Training Expiration Date:	**07/23/2017**
Name of course completed:	**CITI Protection of Human Subjects Research Training - ORA**

Name:	**NORTON, LAURA A**
Department/School/Division:	**ME-Division of Health Policy**
HS Training Completed:	**Yes**
Training Expiration Date:	**03/06/2017**
Name of course completed:	**CITI Protection of Human Subjects Research Training - ORA**

Name:	**REESE, PETER**
Department/School/Division:	**DM-Renal-Electrolyte and Hypertension**
HS Training Completed:	**Yes**
Training Expiration Date:	**09/16/2014**
Name of course completed:	**CITI Protection of Human Subjects Research Training - ORA**

Name:	**CLAPP, JUSTIN T**
Department/School/Division:	**AN-Anesthesia**
HS Training Completed:	**Yes**
Training Expiration Date:	**10/15/2018**
Name of course completed:	**CITI Protection of Human Subjects Research Training - ORA**

Name:	**PUTT, MARY**
Department/School/Division:	**BE-Biostatistics Division**
HS Training Completed:	**Yes**
Training Expiration Date:	**10/22/2016**
Name of course completed:	**CITI Protection of Human Subjects Research Training - ORA**

Name:	**HA, YOONHEE P**
Department/School/Division:	**Health System**
HS Training Completed:	**Yes**
Training Expiration Date:	**12/24/2019**
Name of course completed:	**CITI Protection of Human Subjects Research Training - ORA**

Name:	**LEITNER, AARON**
Department/School/Division:	**Health System**
HS Training Completed:	**Yes**
Training Expiration Date:	**04/16/2018**
Name of course completed:	**CITI Protection of Human Subjects Research Training - ORA**

Name:	**PHILLIPS, CAITLIN**
Department/School/Division:	**Health System**
HS Training Completed:	**Yes**
Training Expiration Date:	**10/24/2019**
Name of course completed:	**CITI Protection of Human Subjects Research Training - ORA**

Name:	**RUSSELL, LOUISE B**
Department/School/Division:	**ME-Division of Health Policy**
HS Training Completed:	**Yes**
Training Expiration Date:	**02/12/2021**
Name of course completed:	**CITI Protection of Human Subjects Research Training - ORA**

Disclosure of Significant Financial Interests*
Does any person who is responsible for the design, conduct, or reporting of this research protocol have a **FINANCIAL INTEREST**?
No

Penn Intellectual Property*
To the best of the Principal Investigator's knowledge, does this protocol involve the testing, development or evaluation of a drug, device, product, or other type of intellectual property (IP) that is owned by or assigned to the University of Pennsylvania?
No

Certification
I have reviewed the *Financial Disclosure and Presumptively Prohibited Conflicts for Faculty Participating in Clinical Trials* and the *Financial Disclosure Policy for Research and Sponsored Projects* with all persons who are responsible for the design, conduct, or reporting of this research; and all required Disclosures have been attached to this application.
Yes

Biomedical Research

Clinical Trial*
Is this a clinical trial?
Yes

If Yes, please be aware that for each clinical trial conducted or supported by a Federal department or agency, one IRB-approved informed consent form used to enroll subjects must be posted by the awardee or the Federal department or agency component conducting the trial on a publicly available Federal Web site that will be established as a repository for such informed consent forms.

Investigator Initiated Trial*
Is this an investigator initiated trial?
Yes

If Yes, please be aware that the investigator may be required to create and manage a record of this trial in https://clinicaltrials.gov.

Drugs or Devices*
Does this research study involve Drugs or Devices?
No

IND Exemption

For studies that fall under an IND exemption, please provide the number below

For studies including IND or IDE's, please provide the number(s) below

IDE Review*
NOTE: For research involving investigational devices, you are required to review the guidance on Managing Research Device Inventory. Consult the Penn Manual for Clinical Research: https://www.med.upenn.edu/pennmanual/secure/investigational-product-management-at-sites-not-using-investigational-drug-services-(ids) html Please check the box Yes if you have reviewed the guidance.
Yes

Research Device Management*
Please indicate how research device(s) will be managed.
Not Applicable (no investigational devices)

Drug, Herbal Product or Other Chemical Element Management *
Please indicate how drugs, herbal products or other chemical entities will be managed.
Not Applicable (no drugs, herbal products or other chemical entities)

Radiation Exposure*
Are research subjects receiving any radiation exposure (e.g. X-rays, CT, Fluoroscopy, DEXA, pQCT, FDG, Tc-99m, etc.) that they would not receive if they were not enrolled in this protocol?
No

Gene Transfer*
Does this research involve gene transfer (including all vectors) to human subjects?
No

Human Source Material*
Does this research include collection or use of human source material (i.e., human blood, blood products, tissues or body fluids)?
Yes

CACTIS and CT Studies*
Does the research involve Center for Advanced Computed Tomography Imaging Services (CACTIS) and CT studies that research subjects would not receive if they were not part of this protocol?
No

CAMRIS and MRI Studies*
Does the research involve Center for Advanced Magnetic Resonance Imaging and Spectroscopy (CAMRIS) and MRI studies that research subjects would not receive if they were not part of this

protocol?
No

Investigational Agent or Device within the Operating Room*
Does the research project involve the use of an investigational agent or device within the Operating Room?
No

Cancer Related research not being conducted by an NCI cooperative group*
Does this protocol involve cancer-related studies in any of the following categories?
No

Processing of Materials*
Will the research involve processing (such as over encapsulating, or compounding)?
No

In-House Manufacturing of Materials*
Will the research involve processing (such as over encapsulating, or compounding)?
No

Medical Information Disclosure*
Does the research proposal involve the use and disclosure of research subject's medical information for research purposes?
Yes

If the answer is YES, indicate which items is is provided with this submission:

Modified research informed consent document that incorporates HIPAA requirements

CTRC Resources*
Does the research involve CTRC resources?
No

Pathology and Laboratory Medicine Resources*
Will samples be collected by hospital phlebotomy and/or processed or analyzed by any of the clinical laboratories of the University of Pennsylvania Health System?
No

Research Involves Apheresis, Cell Collection, and/or Blood Product Collection*
Does this research involve collection of blood products in the Penn Donor Center and/or the use of apheresis for treatment or collection of cells or other blood components?
No

Research involving blood transfusion or drug infusions*
Will your research involve blood transfusion or infusion of study drug in 3 Ravdin Apheresis Unit for research purposes?
No

Trial in Radiation Oncology
Is this research a prospective trial being done in Radiation Oncology, and if so, has this protocol been approved by the Radiation Oncology Protocol committee?
No

Study in Radiation Oncology
Is this research a retrospective study being done in Radiation Oncology, and if so, has this project been reviewed by the Radiation Oncology Clinical Research Group?
No

Use of UPHS services*
Does your study require the use of University of Pennsylvania Health System (UPHS) services, tests or procedures*, whether considered routine care or strictly for research purposes?
No

Primary Focus*
Sociobehavioral (i.e. observational or interventional)

Protocol Interventions

x **Sociobehavioral (i.e. cognitive or behavioral therapy)**
Drug
Device - therapeutic
Device - diagnostic (assessing a device for sensitivity or specificity in disease diagnosis)
Surgical
x **Diagnostic test/procedure (research-related diagnostic test or procedure)**
Obtaining human tissue for basic research or biospecimen bank
x **Survey instrument**
None of the above

The following documents are currently attached to this item:

There are no documents attached for this item.

Department budget code

None

Multi-Site Research

Other Sites

No other sites

Management of Information for Multi-Center Research
n/a

The following documents are currently attached to this item:

There are no documents attached for this item.

Protocol

Abstract
Cardiovascular disease (CVD) is the leading cause of death in the United States. Despite strong evidence that reducing low-density lipoproteins (LDL) with statins successfully lowers CVD risk, more than 50% of patients stop taking statins within one year of initial prescription though such therapy typically should be lifelong. In this study, we will test the effectiveness of different behavioral economic interventions in shaping lasting habits for statin adherence and reducing LDL cholesterol among patients with poor cholesterol control who are at very high risk for CVD. The application of conceptual approaches from behavioral economics offers considerable promise in advancing health and health care. Interventions based on financial incentives are successful in changing health behaviors during the intervention period but in many cases not thereafter. In patients with suboptimal cholesterol control who are at high risk for CVD, we propose to test the effectiveness of different behavioral economic techniques in inducing habit formation for adherence to statin use and sustained reductions in LDL cholesterol after financial incentives are discontinued. We will test these approaches among members of CVS Caremark nationwide. Using a 4-arm, randomized controlled trial, we aim to answer these questions: [1] How do daily sweepstakes, habit formation sweepstakes, and hybrid sweepstake deposit contracts, all of which include daily reminders, compare with daily reminders in improving statin adherence and LDL control during the intervention phase? [2] How do these 3 incentives compare to daily reminders in achieving improved adherence and improved LDL control post-intervention? [3]

How do these approaches compare in cost-effectiveness?

Objectives

Overall objectives

Using a 4-arm, randomized controlled trial, we aim to answer these questions: [1] How do daily sweepstakes, habit formation sweepstakes, and hybrid sweepstake deposit contracts, all of which include daily reminders, compare with daily reminders in improving statin adherence and LDL control during the intervention phase? [2] How do these 3 incentives compare to daily reminders in achieving improved adherence and improved LDL control post-intervention? [3] How do these approaches compare in cost-effectiveness?

Primary outcome variable(s)

The primary outcome variable will be changes in LDL from enrollment to 12 months (6 months after cessation of financial incentives).

Secondary outcome variable(s)

A secondary outcome will be statin adherence in the 6 to 12 months after the active phase of the intervention.

Background

Cardiovascular disease (CVD) is the single leading cause of death in the United States.28 1.2 million Americans each year have a new or recurrent myocardial infarction (AMI) and 38% of them die from it in a given year.1 Clinical practice guidelines recommend HMG-CoA reductase inhibitors (statins) to lower cholesterol,29 and clinical trials have shown that statins lower the risk of AMI by about 30%.2-6, 30 Despite their proven benefits and benign side effect profile, the population effectiveness of statins is limited by rates of adherence to statins that are moderate at best: approximately half of patients prescribed statins discontinue usage within a year.8, 31 Poor adherence leads to worse outcomes, higher hospitalization and mortality rates, and increased health care costs among CVD patients.8, 9, 31-37 However, many seemingly successful efforts to improve medication adherence have been too complex to be implemented or required extensive resources, limiting applicability and sustainability.38, 39 Annual direct and indirect US expenditures attributable to CVD are about $500 billion.1 Statins can reduce CVD events requiring hospitalization by nearly 20%, which could save over $15 billion annually in the United States from CVD and stroke hospitalizations alone. For secondary prevention, the cost-effectiveness ratios of statins range from being cost-saving to approximately $30,000 per quality-adjusted life-year (QALY) gained.40 For primary prevention, cost per QALY ratios are well below accepted thresholds; for example, in the United Kingdom from £10,000 to £31,000 per QALY for 10-year CVD risk ranging from 30% to 5%.40, 41,42 A review of 23 studies suggests that improvement in adherence reduces overall treatment costs, reduces disease-related costs, and improves cost-effectiveness of cardiovascular medications;43 in many cases, small improvements in adherence lead to large improvements in cost-effectiveness ratios.44 Behavioral economists have proposed "asymmetric paternalism as an approach to public policy. 45, 46 Approaches using asymmetric paternalism aim to make it easier for people to make good choices, without restricting those choices, e.g., arranging food on a buffet such that healthy foods are more likely to be chosen.46 Asymmetric paternalism is paternalistic in the sense of attempting to help individuals achieve their own goals, as compared to conventional regulation designed to prevent harm to others. Asymmetric paternalism is asymmetric in the sense of helping individuals prone to making irrational decisions while not limiting freedom of choice and not harming those making informed, deliberate, decisions. Setting default options to the most desirable, beneficial, or popular choices is an example of choice architecture. Using financial incentives to encourage certain behaviors is another example of asymmetric paternalism.20, 21 In our own work we have proposed that biases that ordinarily lead to self-harming behavior can be used in interventions to promote healthy behaviors.20, 47 Individuals put disproportionate value on present relative to future costs and benefits. This present-biased preference 48 typically works against healthy behaviors, However, incentives can be structured (e.g., providing tangible small but frequent positive feedback or rewards) so that present-bias works in favor of adopting healthy behaviors. For patients, the most effective approaches have been those requiring monitoring several times a week, suggesting the importance of frequent feedback.49,50 An important part of our work has been to move beyond thinking about financial incentives as all-or-none, but instead to design the structure and timing of incentives to correspond to established principles of psychology and economics. For example, we have tested the use of daily lotteries for patients to improve medication adherence and weight loss.

Americans spend $48 billion annually on state lottery tickets.51 However, the average pay-out rate across state lotteries is just 52%, ranging from 26-71%.52 Several features combine to make lotteries attractive despite their poor return. Frequent small payoffs give lottery players intermittent positive reinforcement. Feedback is often very rapid: most games have daily draws and instant scratch-off tickets. The small chance of a large payoff is especially attractive because people tend to overweigh small probabilities in making decisions.53, 54 For these reasons, structuring financial incentives as a lottery has several benefits for a daily incentive. Financial incentives need to be designed in view of shaping good and lasting habits in medication adherence and LDL levels. In prior work we showed how lotteries conditional on adhering to medication raise adherence levels as long as lotteries are available but patients revert to prior adherence levels once lotteries are discontinued 19. One way to address this intervention limitation would be to provide patients with lottery-type incentives indefinitely, but clearly that would be less cost-effective than an approach that achieved sustained effects after a time-limited intervention. In this study, we test the effectiveness of different ways of designing time-limited lotteries in inducing sustained behavior change following the time period in which the daily lotteries are offered. Our study will draw on a conceptual framework from both traditional and behavioral economics. Key elements of this are the use of lotteries/sweepstakes and the augmentation of lotteries/sweepstakes by deadlines and loss aversion. The planned approach also leverages prospect theory53, anticipated regret, and incentives designed to improve self-control 48, 55-57l. In all 3 intervention arms, patients will receive incentives via a daily sweepstake structured to take advantage of several effects identified in the behavioral economics literature on incentives. First, consistent with research on the importance of hyperbolic time discounting and the knowledge that small, frequent rewards (and punishments) can have great incentive value,58-61 we will provide patients daily feedback about whether they won (or would have won had they been adherent) via a daily sweepstake. Second, based on research showing that people are motivated by the experience of past rewards and the prospect of future rewards,62 are particularly attracted to small probabilities of large rewards,63 and tend to overweigh small probabilities in making decisions,53 the sweepstakes are tailored to provide frequent small payoffs and infrequent large payoffs. Third, because regret aversion affects decision making under risk,64, 65 non-adherent patients will receive daily feedback about what they would have won had they been adherent, maximizing the threat of regret among people who fail to adhere. The use of deadlines (in the habit formation sweepstake in which participants receive smaller rewards if they only take their medication after receiving a reminder is motivated by the observation that externally imposed deadlines are more effective that self-imposed deadlines.66 Note that as such the sweepstake incentives have a lower expected value in arm 3 than in arm 2 and traditional economics would predict that they would be thereby less effective. However, we anticipate this design will engage patients to think more about adherence and thereby augment effectiveness. The hybrid sweepstake deposit contract arm divides the reward such that half is provided through a sweepstake as in the standard sweepstake arm and the other half is held in a deposit account, which will be paid out at the end of the month. Participants start with $45 in their deposit account and $1.50 gets deducted each day the participant is non-adherent. The balance paid out at the end of the month is proportional to how many days the participant was adherent. Every day we will report the accumulated balance within the deposit account so that participants get daily feedback about the amount they will forfeit in case of non-adherence. Deposit or pre-commitment contracts have become popular as a way of helping individuals augment motivation by leveraging loss aversion, as we know people are much more sensitive to the disutility of losses than the utility of gains 53, to augment ongoing motivation. However, a big limitation in many of the ongoing efforts using deposit contracts has been low rates of participant uptake, as only the most motivated individuals are willing to put their own money at risk; here we overcome that limitation by automatically seeding a deposit contract for the individual if they are adherent. 1. WRITING GROUP MEMBERS, Lloyd-Jones D, Adams RJ, Brown TM, Carnethon M, Dai S, De Simone G, Ferguson TB, Ford E, Furie K, Gillespie C, Go A, Greenlund K, Haase N, Hailpern S, Ho PM, Howard V, Kissela B, Kittner S, Lackland D, Lisabeth L, Marelli A, McDermott MM, Meigs J, Mozaffarian D, Mussolino M, Nichol G, Roger VL, Rosamond W, Sacco R, Sorlie P, Stafford R, Thom T, Wasserthiel-Smoller S, Wong ND, Wylie-Rosett J, Committee obotAHAS, Stroke Statistics Subcommittee. Heart Disease and Stroke Statistics--2010 Update: A Report From the American Heart Association. Circulation. 2010;121(7):e46-215. 2. Baigent C, Keech A, Kearney PM, Blackwell L, Buck G, Pollicino C, Kirby A, Sourjina T, Peto R, Collins R, Simes R. Efficacy and safety of cholesterol-lowering treatment: prospective meta-analysis of data from 90,056 participants in 14 randomised trials of statins. Lancet. 2005;366(9493):1267-1278. 3. Scandinavian Simvastatin Study Group. Randomised trial of cholesterol lowering in 4444 patients with coronary heart disease: the Scandinavian Simvastatin Survival Study (4S). Lancet. 1994;344 (8934):1383-1389. 4. Sacks FM, Pfeffer MA, Moye LA, Rouleau JL, Rutherford JD, Cole TG, Brown L, Warnica JW, Arnold JM, Wun CC, Davis BR, Braunwald E. The effect of pravastatin on coronary

events after myocardial infarction in patients with average cholesterol levels. Cholesterol and Recurrent Events Trial investigators. N Engl J Med. 1996;335(14):1001-1009. 5. Downs JR, Clearfield M, Weis S, Whitney E, Shapiro DR, Beere PA, Langendorfer A, Stein EA, Kruyer W, Gotto AM, Jr. Primary prevention of acute coronary events with lovastatin in men and women with average cholesterol levels: results of AFCAPS/TexCAPS. Air Force/Texas Coronary Atherosclerosis Prevention Study. Jama. 1998;279(20):1615-1622. 6. The Long-Term Intervention with Pravastatin in Ischaemic Disease (LIPID) Study Group. Prevention of cardiovascular events and death with pravastatin in patients with coronary heart disease and a broad range of initial cholesterol levels. The Long-Term Intervention with Pravastatin in Ischaemic Disease (LIPID) Study Group. N Engl J Med. 1998;339(19):1349-1357. 7. Gandara E, Moniz TT, Dolan ML, Melia C, Dudley J, Smith A, Kachalia A. Improving Adherence to Treatment Guidelines. Critical Pathways in Cardiology. 2009;8(4):139-145. 8. Jackevicius CA, Mamdani M, Tu JV. Adherence with statin therapy in elderly patients with and without acute coronary syndromes. JAMA. 2002;288(4):462-467. 9. Newby LK, LaPointe NM, Chen AY, Kramer JM, Hammill BG, DeLong ER, Muhlbaier LH, Califf RM. Long-term adherence to evidence-based secondary prevention therapies in coronary artery disease. Circulation. 2006;113(2):203-212. 10. Benner JS, Glynn RJ, Mogun H, Neumann PJ, Weinstein MC, Avorn J. Long-term persistence in use of statin therapy in elderly patients. JAMA. 2002;288:455-461. 11. Gislason GH, Rasmussen JN, Abildstrom SZ, Gadsboll N, Buch P, Friberg J, Rasmussen S, Kober L, Stender S, Madsen M, Torp-Pedersen C. Long-term compliance with beta-blockers, angiotensin-converting enzyme inhibitors, and statins after acute myocardial infarction. Eur Heart J. 2006;27(10):1153-1158. 12. Kulkarni SP, Alexander KP, Lytle B, Heiss G, Peterson ED. Long-term adherence with cardiovascular drug regimens. Am Heart J. 2006;151(1):185-191. 13. Simpson E, Beck C, Richard H, Eisenberg MJ, Pilote L. Drug prescriptions after acute myocardial infarction: dosage, compliance, and persistence. Am Heart J. 2003;145 (3):438-444. 14. Hudson M, Richard H, Pilote L. Parabolas of medication use and discontinuation after myocardial infarction--are we closing the treatment gap? Pharmacoepidemiol Drug Saf. 2007;16 (7):773-785. 15. Eagle KA, Kline-Rogers E, Goodman SG, Gurfinkel EP, Avezum A, Flather MD, Granger CB, Erickson S, White K, Steg PG. Adherence to evidence-based therapies after discharge for acute coronary syndromes: an ongoing prospective, observational study. Am J Med. 2004;117(2):73-81. 16. Volpp KG, Gurmankin Levy A, Asch DA, Berlin JA, Murphy JJ, Gomez A, Sox H, Zhu J, Lerman C. A randomized controlled trial of financial incentives for smoking cessation. Cancer Epidemiol Biomarkers Prev. 2006;15(1):12-18. 17. Volpp KG, Das A. Comparative Effectiveness: Thinking Beyond Medication A vs. Medication B. N Engl J Med. 2009;361:331-333. 18. John L, Loewenstein G, Troxel A, Norton L, Fassbender J, Volpp KG. Financial Incentives for Extended Weight Loss: A Randomized, Controlled Trial. Journal of General Internal Medicine. 2011;26(6):621-626. 19. Volpp KG, Loewenstein G, Troxel A, Doshi J, Price M, Laskin M, Kimmel SK. A test of financial incentives to improve warfarin adherence. BMC Health Services Research. 2008;8:272. PMCID: PMC2635367. 20. Loewenstein G, Brennan T, Volpp KG. Asymmetric paternalism to improve health behaviors. JAMA. 2007;298(20):2415-2417. 21. Halpern SD, Ubel PA, Asch DA. Harnessing the power of default options to improve health care. New England Journal of Medicine. 2007;357(13):1340-1344. 22. Higgins ST, Badger GJ, Budney AJ. Initial abstinence and success in achieving longer term cocaine abstinence. Exp Clin Psychopharmacol. 2000;8(3):377-386. 23. Kane RL, Johnson PE, Town RJ, Butler M. A structured review of the effect of economic incentives on consumers' preventive behavior. Am J Prev Med. 2004;27(4):327-352. 24. Volpp KG, Troxel AB, Pauly MV, Glick HA, Puig A, Asch DA, Galvin R, Zhu J, Wan F, DeGuzman J, Corbett E, Weiner J, Audrain-McGovern J. A randomized, controlled trial of financial incentives for smoking cessation. . New England Journal of Medicine. 2009;360(7):699-709. 25. Higgins ST, Budney AJ, Bickel WK, Foerg FE, Donham R, Badger GJ. Incentives improve outcome in outpatient behavioral treatment of cocaine dependence. Arch Gen Psychiatry. 1994;51(7):568-576. 26. Sindelar J. Paying for Performance: the power of incentives over habits. Health Economics. 2008;17(4):449-451. 27. Volpp KG, John LK, Troxel AB, Norton L, Fassbender J, Loewenstein G. Financial incentive-based approaches for weight loss: a randomized trial. JAMA. 2008;300(22):2631-2637. 28. Census 2000 Gazetteer Files. 2006 [updated 2006 August 9; cited]; Available from: www.census.gov/geo/www/gazetteer/places2k.html. 29. Gibbons RJ, Abrams J, Chatterjee K, Daley J, Deedwania PC, Douglas JS, Ferguson TB, Jr, Fihn SD, Fraker TD, Jr, Gardin JM, O'Rourke RA, Pasternak RC, Williams SV. ACC/AHA 2002 guidelines update for the management of patients with chronic stable angina: a report of the American College of Cardiology/American Heart Association Task Force on Practice Guidelines (Committee to Update the 1999 Guidelines for the Management of Patients With Chronic Stable Angina). 2002. 30. Ridker PM, Danielson E, Fonseca FA, Genest J, Gotto AM, Jr., Kastelein JJ, Koenig W, Libby P, Lorenzatti AJ, Macfadyen JG, Nordestgaard BG, Shepherd J, Willerson JT, Glynn RJ. Reduction in C-reactive protein and LDL cholesterol and cardiovascular event rates after initiation of rosuvastatin: a prospective study of the JUPITER trial.

Lancet. 2009;373(9670):1175-1182. 31. Ho PM, Spertus JA, Masoudi FA, Reid KJ, Peterson ED, Magid DJ, Krumholz HM, Rumsfeld JS. Impact of medication therapy discontinuation on mortality after myocardial infarction. Arch Intern Med. 2006;166(17):1842-1847. 32. Ho PM, Magid DJ, Masoudi FA, McClure DL, Rumsfeld JS. Adherence to cardioprotective medications and mortality among patients with diabetes and ischemic heart disease. BMC Cardiovasc Disord. 2006;6:48. 33. Ho PM, Rumsfeld JS, Masoudi FA, McClure DL, Plomondon ME, Steiner JF, Magid DJ. Effect of medication nonadherence on hospitalization and mortality among patients with diabetes mellitus. Arch Intern Med. 2006;166(17):1836-1841. 34. Rasmussen JN, Chong A, Alter DA. Relationship between adherence to evidence-based pharmacotherapy and long-term mortality after acute myocardial infarction. JAMA. 2007;297(2):177-186. 35. Wei L, Flynn R, Murray GD, MacDonald TM. Use and adherence to beta-blockers for secondary prevention of myocardial infarction: who is not getting the treatment? Pharmacoepidemiol Drug Saf. 2004;13(11):761-766. 36. Wei L, Wang J, Thompson P, Wong S, Struthers AD, MacDonald TM. Adherence to statin treatment and readmission of patients after myocardial infarction: a six year follow up study. Heart. 2002;88(3):229-233. 37. Blackburn DF, Dobson RT, Blackburn JL, Wilson TW, Stang MR, Semchuk WM. Adherence to statins, beta-blockers and angiotensin-converting enzyme inhibitors following a first cardiovascular event: a retrospective cohort study. Can J Cardiol. 2005;21(6):485-488. 38. Haynes RB, McKibbon KA, Kanani R. Systematic review of randomised trials of interventions to assist patients to follow prescriptions for medications. Lancet. 1996;348(9024):383-386. 39. Haynes RB. Improving Patient Adherence: State of the art, with a special focus on medication taking for cardiovascular disorders. In: Burke LE, Ockene IS, editors. Compliance in Healthcare and Research. Armonk, NY: Futura Publishing Company, Inc.; 2001. p. 3-21. 40. Prosser LA, Stinnett AA, Goldman PA, Williams LW, Hunink MG, Goldman L, Weinstein MC. Cost-effectiveness of cholesterol-lowering therapies according to selected patient characteristics.[Comment]. Annals of Internal Medicine. 2000;132(10):769-779. 41. Johannesson M. At what coronary risk level is it cost-effective to initiate cholesterol lowering drug treatment in primary prevention?. European Heart Journal. 2001;22(11):919-925. 42. National Institute for Clinical Excellence. Guide to the Methods of Technology Appraisal National Health Service, UK April 2004 [updated April 2004; cited]; Available from: http://www nice.org.uk/page.aspx?o=201973. 43. Muszbek N, Brixner D, Benedict A, Keskinaslan A, Khan ZM. The economic consequences of noncompliance in cardiovascular disease and related conditions: a literature review. Int J Clin Pract. 2008;62(2):338-351. PMCID: 2325652. 44. Hughes DA, Bagust A, Haycox A, Walley T. The impact of non-compliance on the cost-effectiveness of pharmaceuticals: a review of the literature. Health Econ. 2001;10(7):601-615. 45. Camerer C, Issacharoff S, Loewenstein G, ODonoghue T, Rabin M. Regulation for conservatives: Behavioral economics and the case for asymmetric paternalism". University of Pennsylvania Law Review. 2003;151(3):1211-1254. 46. Thaler RH, Sunstein CR. Libertarian Paternalism. The American Economic Review 2003;93(2):175-179. 47. Loewenstein G, John LK, Volpp KG. Using Decision Errors to Help People Help Themselves. In: Shafir E, editor. Behavioral Foundations of Policy. New York: Russell Sage Foundation Press; 2009, in press. 48. O'Donoghue T, Rabin M. Doing it now or later. American Economic Review. 1999;89(1):103-124. 49. Higgins ST. Applying Behavioral Economics to the Challenge of Reducing Cocaine Abuse. In: Chaloupka FJ, Grossman M, Bickel WK, Saffer H, editors. The Economic Analysis of Substance Use and Abuse. Cambridge, MA: NBER; 1999. p. 157-174. 50. Higgins ST, Wong CJ, Badger GJ, Ogden DE, Dantona RL. Contingent reinforcement increases cocaine abstinence during outpatient treatment and 1 year of follow-up. J Consult Clin Psychol. 2000;68(1):64-72. 51. Coughlin CC, Garrett TA, Hernandez-Murillo R. The geography, economics, and politics of lottery adoption. Federal Reserve Bank of St Louis Review. 2006;88 (3):165-180. 52. Clotfelter CT, Cook PJ. Selling Hope: State Lotteries in America. Cambridge, MA: Harvard University Press; 1989. 53. Kahneman D, Tversky A. Prospect theory: An analysis of decision under risk. Econometrica. 1979;47:263-291. 54. Prelec D. The Probability Weighting Function. Econometrica. 1998;66(3):497-527. 55. Gul F, Pesendorfer W. Temptation and Self-Control. Econometrica. 2001;69(6):1403-1435. 56. Gul F, Pesendorfer W. Self-control and the theory of consumption. Econometrica. 2004;72(1):119-158. 57. Laibson DI. Golden eggs and hyperbolic discounting. Quarterly Journal of Economics. 1997;62: 443-477. 58. Ainslie G. Specious reward: A behavioral theory of impulsiveness and impulse control. Psychol Bull. 1975;82:463-496. 59. Thaler RH. Some empirical evidence on time inconsistency. Review of Economic Studies. 1981;23:165-180. 60. Loewenstein G, Prelec D. Anomalies in intertemporal choice: Evidence and an interpretation. Quarterly Journal of Economics. 1992;107:573-597. 61. Kirby K. Bidding on the future: evidence against normative discounting of delayed rewards. Journal of Experimental Psychology: General. 1997;126:54-70. 62. Camerer C, Ho T-H. Experience-Weighted Attraction Learning in Normal Form Games. Econometrica. 1999;67:837-874. 63. Loewenstein G, Weber EU, Hsee CK, Welch N. Risk as feelings. Psychological Bulletin. 2001;127(2):267-286. 64. Connolly T, Butler DU. Regret in Economic

and Psychological Theories of Choice. Journal of Behavioral Decision Making. 2006;19(2):148-158. 65. Zeelenberg M, Pieters R. Consequences of regret aversion in real life: The case of the Dutch postcode lottery. Organizational Behavior & Human Decision Processes. 2004;93(2):155-168. 66. Ariely D, Wertenbroch K. Procrastination, deadlines, and performance: self-control by precommitment. Psychol Sci. 2002;13(3):219-224.

Study Design

Phase*
Not applicable

Design
We propose a 4-arm RCT to test the relative effectiveness and cost-effectiveness of several innovative approaches to improving LDL control through statin use in patients at high risk for CVD. All incentives in arms 2-4 will be awarded monthly based on statin adherence during the intervention period. Adherence in all groups, including arm 1, will be measured using Vitality GlowCaps, MedSignals/ Vitalsignals, [redacted], or Wisepill as a recording device and in all arms patients will receive daily reminders to take their statins. Patients will be randomized evenly into one of 4 arms. In the Control arm, patients will get electronic reminders daily to match the timing of taking a statin on a daily basis. As the focus of the other arms is on the structure of sweepstake payments that include daily reminders it is necessary for a clean comparison to have daily reminders about adherence in arm 1 as well. In arms 2-4, patients will get adherence feedback electronically with daily sweepstake awards conditional on adherence during each day of a 6-month intervention period. In the simple sweepstake arm, we will make participants eligible for daily sweepstakes, where winning is conditional on adherence the previous day. In the habit formation sweepstake arm, we aim to activate patients mental accounting so they learn to be adherent rather than to just respond to the monetary sweepstake incentive. Incentive eligibility will proceed similarly to the simple sweepstake but the sweepstake prizes are halved whenever participants take their medication after they receive a reminder at a pre-announced time. In the hybrid sweepstake deposit arm, we package together two approaches. As patients discount future benefits of being adherent, we make the benefits of adherence more immediate using the standard sweepstake described in the simple sweepstake arm above but with half the reward. Then we take the other half of the expected reward ($45) and use this to seed a deposit contract that decreases by $1.50 each day the participant is non-adherent. This will reset monthly and participants will receive the balance of the deposit account at the end of the month. As the focus is on habit formation, we will follow all patients for six to 12 months after the cessation of the incentive payments at month 6 to measure the degree to which improvements in LDL and adherence are sustained. Sweepstake winnings will be paid out monthly. This design allows a variety of comparisons across arms, answering conceptually and procedurally important questions in the application of behavioral economic approaches to advance health: 1. How does the provision of simple, habit formation, and hybrid sweepstake deposit contracts conditional on statin adherence with daily reminders compare to just daily reminders in terms of adherence and improved LDL control during the intervention phase? 2. How does the provision of standard, habit formation, and hybrid sweepstake deposit contracts compare to just daily reminders in terms of achieving improved adherence and improved LDL control post-intervention? 3. How do these approaches compare in cost-effectiveness in improving LDL control? Patients will receive an active intervention for 6 months followed by 6 to 12 months of observation without incentives or other intervention to examine sustainability post-intervention. The primary outcome will be change in LDL cholesterol from baseline to 12 months. Incentives for patients will be structured as an adherence-based sweepstake with an expected value of $2.80. Study sites. The study will be run by faculty and staff based at the LDI CHIBE. Study participants will be recruited by Penn Medicine and CVS Caremark on behalf of the health plans as a business associate. As a PBM, and business associate to the health plans, CVS Caremark provides services for and maintains data on approximately 40 million members of employer-sponsored health plans across the United States, the number who are on statins and have diabetes or CVD and MPR80% is more than sufficient for our study. CVS Caremark will identify potential participants by reviewing pharmacy claims records it holds as a PBM and business associate to employer-sponsored health using our eligibility criteria of statin use and MPR80%. We are working with CVS Caremark not only because they have a sufficiently large population of potentially eligible participants but because testing this type of approach in partnership with them dramatically increases the likelihood of the research being translated into practice. The study will be run by UPENN investigators, who will communicate with participants through the Way to Health portal, by phone, and by email. Inclusion criteria. Individuals at high risk of a cardiac event,

specifically one of the following: -Individuals with clinical CVD (defined as diagnosis with myocardial infarction, stroke, or peripheral vascular disease) with an LDL greater than or equal to 100 mg/dl ; -Individuals with Diabetes (between the ages of 40-75) with an LDL greater than or equal to 100 mg/dl; -Individuals without clinical CVD or diabetes with LDL greater than or equal to 100 mg/dl and estimated 10-year CVD risk 7.5%; -Individuals without clinical CVD or diabetes with LDL cholesterol greater than or equal to 190 mg/dl A prescription filled for a statin medication within the last 12 months (derived from pharmacy records); Imperfect statin adherence level as defined by one of the following: -Medication Possession Ratio (MPR) less than or equal to 80% -A score 0 on the 8-item Morisky Medication Adherence Questionnaire completed during enrollment Exclusion criteria. Patients will be excluded if they have a known allergy or history of side effects to statins, are less than 18 years old (see Protection of Human Subjects), will not or cannot give consent, are currently participants in another experimental study, have a markedly shortened life expectancy (diagnosis of metastatic cancer, or dementia), active or progressive liver disease, or are prescribed by their doctor PCSK9 inhibitor injections coupled with statin therapy. Study procedures. Recruitment. Study participants will be recruited by Penn Medicine or CVS Caremark on behalf of the health plans as a business associate in its role as a PBM. CVS Caremark will identify potential participants by reviewing pharmacy claims records it holds as a PBM and business associate to health plans using our eligibility criteria of statin use and MPR less than 80%. The pharmacy claims records are Protected Health Information ("PHI") governed by the HIPAA Privacy Rule. CVS Caremark holds the pharmacy claims data in its role as a PBM and business associate to employer-sponsored health plans. As a result, CVS Caremark must obtain the consent of the health plans prior to using the pharmacy claims records for these recruitment purposes. Further, CVS Caremark must be authorized by its business associate agreement with the health plans to conduct the preparatory to research activities To do so, CVS Caremark must demonstrate to the health plans that CVS Caremark's use of pharmacy claims data to identify health plan members who meet the research study's criteria, and to then contact qualified health plan members to recruit them to enroll in the study, is permissible under the HIPAA Privacy Rule. See Subject Confidentiality and Subject Privacy, below. Potential participants will be identified by reviewing CVS-Caremark pharmacy claims records using our eligibility criteria of statin use and MPR less than 80%. CVS-Caremark will facilitate communication to these individuals using existing communication channels (email, mail) by forwarding recruitment materials created by Penn. Potentially eligible individuals will be invited to visit the WTH portal or to call our study staff to enroll. Participants will also be recruited from the University of Pennsylvania Health System. Potential participants will be identified by monitoring laboratory data, via weekly queries of the EPIC electronic medical record database. Primary care providers in the Penn Health System will be notified when one of their patients are eligible to participate via the secure UPHS email system. Providers will have one week to opt patients out of receiving the recruitment letter if they feel the patient is not fit to participate in research. All eligible Penn Health System patients will be mailed letters by the study staff inviting them to sign up on the study website. Follow-up phone calls and emails will also be completed with these patients since contact information will be available through EPIC. If a participant has a direct LDL value meeting study inclusion criteria within the past 4 weeks, that value will be used as their baseline LDL and they will not need to complete the baseline lab test. Baseline pre-treatment assessment for patients. Potentially eligible patients will be sent letters inviting them to participate. Patients interested in enrolling who agree to provide written consent (See Protection of Human Subjects) will complete an intake form and consent using our Way to Health web portal. Scheduling of baseline LDL and/or ensuring LDL greater than or equal to 100 (if a diagnosis of CVD or diabetes) or LDL greater than 190 for non-diabetic or CVD individuals will be the final step in confirming eligibility. Penn Medicine patients will receive a follow-up phone call from a study coordinator one week after the recruitment letter is sent to them. At this time, the coordinator will describe the study to the participant and if interested, will offer to begin enrollment over the phone. The participant will verbally complete the screening survey with the coordinator recording their answers in Way to Health. The coordinator will then go over the consent form with the participant and ask if they agree to take part in the study. If they agree to participate the participant will be mailed a copy of the consent form. The participant will then be asked to log on to the platform to complete the baseline survey before being fully enrolled into the study. This ensures that the participant has internet access and will be able to engage with the platform throughout the study. Additionally, we will incorporate a sweepstakes to our recruitment process. Participants can enter a sweepstake by completing and submitting their profile. They do not need to participate in the trial and participating in the trial will not change the likelihood of winning the prize. Beginning on the first day of each month at 12:00 AM (EDT) through the last day of each month at 11:59 PM (EDT) (the Promotion Period) confirm contact details by using the link found in the letter accompanying these rules. Valid entries must be submitted by 11:59 PM (EDT) before the end date of

the Promotion Period. The last contest ends on December 31 2016 at 11:59pm EST. Winner Selection: At the conclusion of the Promotion Period, one (1) winner will be selected in a random drawing from among all eligible entries received. Drawing will be conducted by Sponsor whose decisions are final. Odds of winning will depend upon the total number of eligible entries received. Prizes & Approximate Retail Value: A total of one (1) winner will receive a new Apple® iPad Air®(WiFi + 16GB, approximate retail value $399. Winners will be notified via email, at Sponsors discretion, within 10 business days following the drawing. Randomization. Randomization of patients to one of the 4 study arms will be performed through the Way to Health platform. Randomization will be stratified by employer, only if more than one employer is involved, and will use block randomization with variable block sizes. After confirmation of patient eligibility, research staff will notify each patient participant of assignment using their preferred means of communication (text message, email, phone) and ask for confirmation of receipt. Participants will be given detailed instructions via Way to Health, phone or by mail for the arm of the study to which they have been assigned and patients in all arms will be given the GlowCaps/electronic pill container and instructions on use. Patients will be instructed to call study staff for all questions or problems with GlowCaps/electronic pill container use. Scheduling. Direct low density lipoprotein (LDL) and a full lipid panel will be performed for each participant at baseline, 6 months, 12 months and possibly 18 months. Surveys will be completed at baseline, 6, and 12 months. The amount of blood drawn at baseline will be 3 mL and the amount drawn at the remainder of the lab tests will be 1 mL. Participants will return GlowCaps after they complete the study in 12 months. The Way to Health participant tracking system will automatically remind the study coordinators that each enrolled subject has a scheduled follow-up visit at the end of months 6 and 12. We will obtain extensive contact information from each participant and update it at each follow-up visit. We will call participants who miss follow-up visits weekly for 4 weeks and send 2 letters during these 4 weeks. If any participants appear lost to follow-up, we will call their primary physician to ascertain their status. To enhance retention, those who enroll in the study will be compensated $25 for baseline (if they require a lab test), $25 if they are eligible and start in the study, and an additional $75 for 6,12 and 18 months. Generous participation incentives have succeeded in minimizing differential drop out in our previous studies. Implementation of intervention arms. For each of the incentive arms, eligibility for an incentive will be based on daily statin adherence as described in Section B.3.d.i but the details of the incentive design implementation varies as described below. Standard sweepstake incentive. At study entry, we will assign each participant a two-digit number. Each day GlowCaps/electronic pill container will automatically upload adherence data for the previous day via the internet to a secure server housed at UPENN. The Way to Health system generates two-digit random daily sweepstake numbers and compares them electronically to the participants two-digit identification number to determine eligibility for awards. In the standard sweepstake arm, if the two digit number matches, which will happen about 1 in 100 times, the participant will be eligible to win $100 if s/he was adherent the day before. If the two digit number does not match, but either the first digit or second digit matches in the right place, the subject is eligible to win $10, which will happen about 1 in 5 times (more precisely, 18 in 100 times). The expected value of this sweepstake is $2.80/day such that total winnings per 6 months for a fully adherent participant have an expected value of $512.40 (but could be more or less for an individual participant depending upon chance). As in our previous studies, we have designed the sweepstake-based incentives so that, each day, adherent participants receive rapid feedback about whether they won, and non-adherent participants receive rapid feedback about whether they would have won had they been adherent. This program incorporates key aspects of optimal design, including objective and reliable confirmation of behavior change at frequent intervals, large potential payments to reinforce the target behavior, frequent reinforcement using smaller payments, and the use of anticipated regret, a powerful motivator. We set the expected value of the sweepstake at about $3/day based on our success in significantly affecting weight loss and medication adherence with these parameters. In addition, there is evidence that a patient with established CVD has health expenditures ranging from $18,000-$30,000 per year1, suggesting that incentives of $3 a day, if effective, could be cost effective due to the potential savings. Information on whether the participant won will be electronically transmitted each day by text message, phone, or email (patient choice) and will also be available on the patients page on the Way to Health portal. Habit formation incentive: Same as in the standard sweepstake except that participant will receive half of the daily incentive if they do not take their statin before receiving a reminder at 9pm (the exact time can be altered per patient preference) Hybrid sweepstake and Deposit contract: Same as in the standard sweepstake whereby patients automatically enroll into a daily sweepstake when being adherent except that the sweepstakes is only worth half of the amount. Participants in this arm will receive $5 for matching one number and $50 for matching two numbers. Additionally, $45 will be placed in a deposit account until the end of the month and will decrease by $1.50 every day the participant is non-adherent. Participants will receive the balance in their deposit account at the end of

each month. When the next month begins, the deposit account re-sets to $45. Measures LDL cholesterol (primary outcome). The evidence base linking improvements in LDL cholesterol to reductions in CVD is extensive, supporting about a 20% reduction in CVD per 40 mg/dl reduction. LDL cholesterol is easily monitored through a simple blood test. The primary outcome will be change in LDL between baseline (prior to randomization) and 12 months. Adherence measured using Vitality GlowCaps, MedSignals/Vitalsignals, [redacted] or Wisepill /an electronic pill container (secondary outcome). Several methods have been used to measure adherence; no gold standard exists. There are multiple limitations to methods such as patient self-report and pill counts. GlowCaps/electronic pill container provides an unbiased assessment of pill bottle opening and a valid approach to verifying self-administered pill taking, reflecting not only daily use but also patterns of drug use and timing. This method assumes that each time the cap is opened, a dose is taken, and that doses are not taken when the cap is not opened. GlowCaps are just like regular pill bottles so there is little need for patients to decant pills into other containersa process that can lead to false negative measures of adherence. Similarly, although it is possible for patients to open a pill bottle but not take their medication, evidence suggests that once an individual opens a pill bottle, pills are nearly always taken and numerous studies have established the validity of electronic pill container measures. Each day the GlowCap/electronic pill container will electronically transmit whether a participant opened his/her prescription bottle to take his/her statin via a built-in modem to the central server (there is no internet charge to participants) and a simple wireless device plugged into an outlet. Participants will be considered adherent only if we receive electronic notification signaling that the statin bottle was opened once the previous day. GlowCaps and the wireless transmitters are easily portable and can be used while traveling. Each patient will receive instructions to call the study nurse for any changes in dose frequency (an unlikely event in the context of statins, all of which are recommended as once-a-day medications), in which case the GlowCap will be reprogrammed.

Study duration

This is a four year study. The estimated time for enrolling all subjects through completing all follow ups is 38 months. Each subject's participation is 12-18 months (6 months intervention plus 6-12 months of follow-up). The proposed project dates are September 2012 - September 2016.

Resources necessary for human research protection

Describe research staff and justify that the staff are adequate in number and qualifications to conduct the research. Describe how you will ensure that all staff assisting with the research are adequately informed about the protocol and their research related duties. Please allow adequate time for the researchers to conduct and complete the research. Please confirm that there are adequate facilities for the research.

The project will take place at the Leonard Davis Institute Center for Health Incentives and Behavioral Economics (LDI CHIBE) at the University of Pennsylvania (UPENN) in partnership with CVS Caremark, with whom the LDI CHIBE has a behavioral economics research partnership. The team will also have support from Louise Russell from Rutgers and George Loewenstein from Carnegie Mellon University; these sites provide substantial research experience, infrastructure support, and expertise in areas important to this project. The team includes investigators experienced in clinical medicine, health behavior interventions, clinical trials, behavioral economics, cost-effectiveness analysis, and program evaluation. Details are provided in the Biosketches and Budget Justification, but expertise is highlighted below. Multiple PIs: Dr. Iwan Barankay is Associate Professor of Management at the Wharton School and a Sloan Research Fellow. He has extensive experience designing and implementing field studies to evaluate a broad range of performance incentives. Dr. Kevin Volpp directs the LDI CHIBE and the NIA-funded PENN-CMU Roybal P30 Center on Behavioral Economics and Health and is a Professor of Medicine at the Perelman School of Medicine (SOM) and Professor of Health Care Management at the Wharton School at UPENN. He has led numerous studies of patient financial incentives. Dr. Peter Reese is an internist with experience using Vitality GlowCaps/electronic pill container as an adherence tool in a clinical trial setting; he has also examined the role of financial incentives in different clinical contexts including organ donation. Behavioral Economics: Dr. George Loewenstein (Consultant) is the Herbert A. Simon Professor of Economics and Psychology at Carnegie Mellon University and a founder of the fields of behavioral economics and neuroeconomics. Statistical Analysis: Dr. Mary Putt (Co-I, Statistician) is Professor of Biostatistics at UPENN and Director of two NIH-funded Biostatistics Cores. She has 18 years of experience in the design, conduct, and analysis of clinical studies, including trials involving repeated measurements. Cost Effectiveness Analysis: Dr. Louise Russell (Co-I) is Research Professor at the Institute for Health and Professor in the Department of Economics, Rutgers University. She is an international leader in the methods and application of cost-effectiveness analysis.

A member of the National Academy of Medicine since 1983, Dr. Russell co-chaired the U.S. Public Health Service Panel on Cost-Effectiveness in Health and Medicine. Advisory Board: Dr. Troy Brennan is Chief Medical Officer at CVS-Caremark corporation and a board member for the LDI CHIBE as well as a member of the IOM; John List is the Homer J. Livingston Professor of Economics at the University of Chicago; Uri Gneezy is the Art Brody Chair in Behavioral Economics at the University of California San Diegos Rady School of Management. In-person meetings among the UPENN, Rutgers, and CMU team members are planned upon project initiation and quarterly thereafter. Ongoing project management will be facilitated by weekly or bi-weekly meetings of the UPENN team and the use of Basecamp online project management software. Team members will correspond as frequently as needed via email and telephone. To minimize the impact of geographic distance between the participation sites, the team will use available technologies appropriate to the particular meeting, including videoconferencing or webinar. The team will be organized similarly to how we have conducted multi-center studies previously, with project leaders at both the staff and faculty level and clear lines of responsibility for achievement of milestones.

Characteristics of the Study Population

Target population

Study participants will be recruited by Penn Medicine or CVS Caremark on behalf of the health plans as a business associate. As a PBM, CVS Caremark provides services for and maintains data on approximately 40 million members of employer-sponsored health plans across the United States, the number who are on statins and have diabetes or CVD and MPR less than 80% is more than sufficient for our study. CVS Caremark will identify potential participants by reviewing pharmacy claims records using our eligibility criteria of statin use and MPR less than 80%. We are working with CVS Caremark not only because they have a sufficiently large population of potentially eligible participants but because testing this type of approach in partnership with them dramatically increases the likelihood of the research being translated into practice. The study will be run by UPENN investigators, who will communicate with participants through the Way to Health portal, by phone, and by email.

Subjects enrolled by Penn Researchers

800

Subjects enrolled by Collaborating Researchers

0

Accrual

Potential participants will be identified by reviewing CVS Caremark reviewing the pharmacy claims records it holds as a PBM and business associate to health plans and using our eligibility criteria of statin use and MPR80%. CVS Caremark will facilitate communication to these members using existing communication channels (email, mail). Potentially eligible individuals will be invited to visit the WTH portal or to call our study staff to enroll. Patients interested in enrolling who agree to provide written consent will complete an intake form and consent using our Way to Health web portal. Scheduling of baseline LDL and ensuring LDL greater than or equal to 100 (if a diagnosis of CVD or diabetes) or LDL greater than 190 for non-diabetic or CVD individuals will be the final step in confirming eligibility. Participants will also be recruited from the University of Pennsylvania Health System. Potential participants will be identified by monitoring laboratory data, via weekly queries of the EPIC electronic medical record database. Primary care providers in the Penn Health System will be notified when one of their patients are eligible to participate via the secure UPHS email system. Providers will have one week to opt patients out of receiving the recruitment letter if they feel the patient is not fit to participate in research. All eligible Penn Health System patients will be mailed letters by the study staff inviting them to sign up on the study website. Follow-up phone calls, emails and text messages will also be completed with these patients since contact information will be available through EPIC. If a participant has a direct LDL value meeting study inclusion criteria within the past 4 weeks, that value will be used as their baseline LDL and they will not need to complete the baseline lab test. Potentially eligible patients will be sent opt-out letters stating that they are pre-enrolled into the study and they have one of three options. Option 1: Participant completes enrollment online by visiting pennheartstudy.com (this consists of screening survey, consent, W-9 and baseline survey). If the

participant completes enrollment online within a week of sending the recruitment letter, they do not receive a phone call from the study coordinator. After the participant enrolls, the study coordinator mails them a package with a Glowcap and instructions. The participant receives future communications from the study website. Lab visits are scheduled at 6 and 12 months after the participant enrolls in the study. Option 2: Participant does not enroll online within a week of mailing the recruitment letter. Coordinator uses Google voice to send the following HIPAA-compliant message via text to participants that have cell phone numbers available in the EPIC database "This is Darra Finnerty at Penn Medicine. We pre-enrolled you in a health improvement study. When is a good time to call you to complete enrollment?" The study coordinator records the time requested by the participant and calls the participant at the requested time to complete enrollment by phone (this consists of screening survey, consent, W-9 and baseline survey). If the participant does not reply to the text message, the study coordinator makes call attempts at varying times during normal business hours (Mon-Fri between 9-5pm). Once the phone enrollment is complete, participants are mailed a package with a Glowcap and instructions. Labs visits are scheduled at 6 and 12 months after the participant enrolls in the study. Should the participant reply to the SMS message that s/he is not interested in the study, then that persons will be removed from the contact list and will no longer be called or mailed. Note that if a participant "opts -out" (option 3), they would not receive the text message. Option 3: Participant emails study coordinator if they want to "opt-out" of the study. Participants that "opt-out" are removed from our recruitment list and do not receive a phone call or text message to complete their enrollment. Additionally, we will incorporate a sweepstakes to our recruitment process. Participants can enter a sweepstake by completing and submitting their profile. They do not need to participate in the trial and participating in the trial will not change the likelihood of winning the prize. Randomization of patients to one of the 4 study arms will be performed through the Way to Health platform. Randomization will be stratified by employer, if more than one employer is involved, and will use block randomization with variable block sizes. After confirmation of patient eligibility, research staff will notify each patient participant of assignment using their preferred means of communication (text message, email, phone) and ask for confirmation of receipt. Participants will be given detailed instructions via Way to Health, phone or by mail for the arm of the study to which they have been assigned and patients in all arms will be given the GlowCaps and instructions on use. Patients will be instructed to call study staff for all questions or problems with GlowCaps use. Statistical Methods for determining sample size: LDL cholesterol is strongly associated with CVD outcomes so much so that even small movements in LDL are clinically meaningful. We use a 10 mg/dl change as our threshold, based on a meta-analysis by the Cholesterol Treatment Trialists (CTT) Collaboration on 90,000 patients from 14 trials in which such a change would equal about a 5% reduction in CVD events. We developed a two-step approach to the power calculation that reflects our intention to first make comparisons between each of the interventions arms #2 - 4 versus control (3 comparisons), and then to compare any intervention arms that differs from the control (up to 3 additional comparisons). For these sample size estimates, we assumed a standard deviation of 24.5 mg/dl (based on a prior RCT of adherence in which participants had a mean adherence rate of 60%). For the first phase of testing (comparisons of each intervention arms to the control arm), we required sufficient power to detect a 10 mg/dl difference between intervention arms. Further, to accommodate the three hypotheses to be tested, we use a standard Holm Bonferroni correction to maintain the experiment-wise Type 1 error at the nominal level of 0.05. We further assume 20% loss to follow up for the LDL measurement at 12 months. Using simulations based on these assumptions, we estimate that 200 participants in each of the 4 arms will provide at least 90% power to detect a 10 mg/dl difference between interventions. This number of patients also provides at least 80% power to detect an 8.5 mg/dl mean difference between at least one pair of intervention arms, using a Tukey Honest Significant Difference approach that employs the studentized range distribution to assess the significance of pairwise comparisons while maintaining control of the Type I error rate. The smaller 8.5 mg/dl difference between intervention arms is consistent with the idea that differences among active arms are likely to be smaller in magnitude than the differences between active arms and control.

Key inclusion criteria
Individuals at high risk of a cardiac event, specifically one of the following: - Individuals with clinical CVD (defined as diagnosis with myocardial infarction, stroke, or peripheral vascular disease) with an LDL greater than or equal to 100 mg/dl ; - Individuals with Diabetes (between the ages of 40-75) with an LDL greater than or equal to 100 mg/dl; - Individuals without clinical CVD or diabetes with LDL greater than or equal to 100 mg/dl and estimated 10-year CVD risk 7.5%; - Individuals without clinical CVD or diabetes with LDL cholesterol greater than or equal to 190 mg/dl A prescription filled for a statin medication within the last 12 months (derived from pharmacy records); Imperfect statin adherence level as defined by one of the following: - Medication Possession Ratio (MPR) less than or

equal to 80% - A score 0 on the 8-item Morisky Medication Adherence Questionnaire completed during enrollment

Key exclusion criteria
Younger than 18 years old Have a contraindication to further statin use or have suffered side effects from statins, such as myopathy Will not or cannot give consent Have a history of active or progressive liver disease Are currently participating in another clinical trial with related aims Have co-morbidities likely to lead to death within a short-period (e.g. metastatic cancer). No one will be excluded on the basis of sex or race.

Vulnerable Populations

Children Form

Pregnant women (if the study procedures may affect the condition of the pregnant woman or fetus) Form

Fetuses and/or Neonates Form

Prisoners Form

Other

x **None of the above populations are included in the research study**

The following documents are currently attached to this item:

There are no documents attached for this item.

Populations vulnerable to undue influence or coercion
All participants will be mentally able, literate, working adults participating in the study of their own free will. This population is not unusually vulnerable.

Subject recruitment
Study participants will be recruited by Penn Medicine or CVS Caremark on behalf of the health plans as a business associate. CVS Caremark will identify potential participants by reviewing its pharmacy claims records using our eligibility criteria of statin use and MPR less than 80%. The pharmacy claims records are Protected Health Information ("PHI") governed by the HIPAA Privacy Rule. CVS Caremark holds the pharmacy claims data in its role as a PBM and business associate to employer-sponsored health plans. As a result, CVS Caremark must obtain the consent of the health plans prior to using the pharmacy claims records for these recruitment purposes. Further, CVS Caremark must be authorized by its business associate agreement with the health plans to conduct the preparatory to research activities. See Subject Confidentiality and Subject Privacy, below. CVS Caremark will facilitate communication to these individuals using existing communication channels (email, mail) by forwarding recruitment materials created by Penn. Potentially eligible individuals will be invited to visit the WTH portal or to call our study staff to enroll. Participants will also be recruited from the University of Pennsylvania Health System. Potential participants will be identified by monitoring laboratory data, via weekly queries of the EPIC electronic medical record database. Primary care providers in the Penn Health System will be notified when one of their patients are eligible to participate via the secure UPHS email system. Providers will have one week to opt patients out of receiving the recruitment letter if they feel the patient is not fit to participate in research. All eligible Penn Health System patients will be mailed letters by the study staff inviting them to sign up on the study website. Follow-up phone calls, emails and text messages will also be completed with these patients since contact information will be available through EPIC. If a participant has a direct LDL value meeting study inclusion criteria within the past 4 weeks, that value will be used as their baseline LDL and they will not need to complete the baseline lab test. Potentially eligible patients will be sent opt-out letters stating that they are pre-enrolled into the study and they have one of three options. Option 1: Participant completes enrollment online by visiting pennheartstudy.com (this consists of screening survey, consent, W-9 and baseline survey). If the participant completes enrollment online within a week of sending the recruitment letter, they do not receive a phone call from the study coordinator. After the participant enrolls, the study coordinator mails them a package with a Glowcap and instructions. The participant receives future communications from the study website. Lab visits are scheduled at 6 and 12 months after the participant enrolls in the study. Option 2: Participant does not enroll online within a week of mailing the recruitment letter. Coordinator uses Google voice to send the following HIPAA-compliant message via

text to participants that have cell phone numbers available in the EPIC database "This is Darra Finnerty at Penn Medicine. We pre-enrolled you in a health improvement study. When is a good time to call you to complete enrollment?" The study coordinator records the time requested by the participant and calls the participant at the requested time to complete enrollment by phone (this consists of screening survey, consent, W-9 and baseline survey). If the participant does not reply to the text message, the study coordinator makes call attempts at varying times during normal business hours (Mon-Fri between 9-5pm). Once the phone enrollment is complete, participants are mailed a package with a Glowcap and instructions. Labs visits are scheduled at 6 and 12 months after the participant enrolls in the study. Should the participant reply to the SMS message that s/he is not interested in the study, then that persons will be removed from the contact list and will no longer be called or mailed. Note that if a participant "opts -out" (option 3), they would not receive the text message. Option 3: Participant emails study coordinator if they want to "opt-out" of the study. Participants that "opt-out" are removed from our recruitment list and do not receive a phone call or text message to complete their enrollment. Additionally, we will incorporate a sweepstakes to our recruitment process. Participants can enter a sweepstake by completing and submitting their profile. They do not need to participate in the trial and participating in the trial will not change the likelihood of winning the prize. Randomization of patients to one of the 4 study arms will be performed through the Way to Health platform. Randomization will be stratified by employer, if more than one employer is involved, and will use block randomization with variable block sizes. After confirmation of patient eligibility, research staff will notify each patient participant of assignment using their preferred means of communication (text message, email, phone) and ask for confirmation of receipt. Participants will be given detailed instructions for the arm of the study to which they have been assigned and patients in all arms will be given the GlowCaps and instructions on use. Patients will be instructed to call study staff for all questions or problems with GlowCaps use.

Will the recruitment plan propose to use any Penn media services (communications, marketing, etc.) for outreach via social media avenues (examples include: Facebook, Twitter, blogging, texting, etc.) or does the study team plan to directly use social media to recruit for the research?
No

The following documents are currently attached to this item:

There are no documents attached for this item.

Subject compensation*
Will subjects be financially compensated for their participation?
Yes

The following documents are currently attached to this item:

There are no documents attached for this item.

If there is subject compensation, provide the schedule for compensation per study visit or session and total amount for entire participation, either as text or separate document

Those who enroll in the study will be compensated $25 for their first series of tests (a subset of participants from the Home Depot population will receive this $25 in the form of an Amazon Gift Card instead of a check), $25 if they are eligible and start in the study, and $75 each for their proceeding series of tests. Patients who undergo eligibility screening but do not enroll in the study will be compensated $25 for their time. Generous participation incentives have succeeded in minimizing differential drop out in our previous studies. For those randomized to an incentive arm, each participant will be assigned a two-digit numbers. Each day GlowCaps will automatically upload adherence data for the previous day via the internet to a secure server housed at UPENN. The Way to Health system generates two-digit random daily sweepstake numbers and compares them electronically to the participants two-digit identification number to determine eligibility for awards. Standard Sweepstake Arm: In the standard sweepstake arm, if the two digit number matches, which will happen about 1 in 100 times, the participant will be eligible to win $100 if s/he was adherent the day before. If the two digit number does not match, but either the first digit or second digit matches in the right place, the subject is eligible to win $10, which will happen about 1 in 5 times (more precisely, 18 in 100 times). The expected value of this sweepstake is $2.80/day such that total winnings per 6 months for a fully

adherent participant have an expected value of $512.40 (but could be more or less for an individual participant depending upon chance). Habit formation incentive: Same as in the standard sweepstake except that participant will receive half of the daily incentive if they do not take their statin before receiving a reminder at 9pm (the exact time can be altered per patient preference) Hybrid Sweepstake and Deposit contract: Same as in the standard sweepstake whereby patients automatically enroll into a daily sweepstake when being adherent except that the sweepstakes is only worth half of the amount. Participants in this arm will receive $5 for matching one number and $50 for matching two numbers. Additionally, $45 will be placed in a deposit account until the end of the month and will decrease by $1.50 every day the participant is non-adherent.. Participants will receive the balance in their deposit account at the end of each month. When the next month begins, the deposit account re-sets to $45.

Study Procedures

Suicidal Ideation and Behavior

Does this research qualify as a clinical investigation that will utilize a test article (ie- drug or biological) which may carry a potential for central nervous system (CNS) effect(s)?

No

Procedures

We propose a 4-arm RCT to test the relative effectiveness and cost-effectiveness of several innovative approaches to improving LDL control through statin use in patients at high risk for CVD. All incentives in arms 2-4 will be awarded monthly based on statin adherence during the intervention period. Adherence in all groups, including arm 1, will be measured using Vitality GlowCaps, Medsignals, [redacted] or Wisepill as a recording device and in all arms patients will receive daily reminders to take their statins. Patients will be randomized evenly into one of 4 arms described in Figure 5 (attached). In the Control arm, patients will get electronic reminders daily to match the timing of taking a statin on a daily basis. As the focus of the other arms is on the structure of sweepstake payments that include daily reminders it is necessary for a clean comparison to have daily reminders about adherence in arm 1 as well. In arms 2-4, patients will get adherence feedback electronically with daily sweepstake awards conditional on adherence during each day of a 6-month intervention period. In the simple sweepstake arm, we will make participants eligible for daily sweepstakes, where winning is conditional on adherence the previous day. In the habit formation sweepstake arm, we aim to activate patients mental accounting so they learn to be adherent rather than to just respond to the monetary sweepstake incentive. Incentive eligibility will proceed similarly to the simple sweepstake but the sweepstake prizes are halved whenever participants take their medication after they receive a reminder at a pre-announced time. In the hybrid sweepstake deposit arm, we package together two approaches. As patients discount future benefits of being adherent, we make the benefits of adherence more immediate using the standard sweepstake described in the simple sweepstake arm above but with half the reward. Then we seed a deposit contract with $45 at the beginning of each month that decreases every day that the participant in non-adherent. This will reset monthly and participants will receive their deposit account balance at the end of the month. As the focus is on habit formation, we will follow all patients for six months after the cessation of the incentive payments at month 6 to measure the degree to which improvements in LDL and adherence are sustained. Sweepstake winnings will be paid out monthly. This design allows a variety of comparisons across arms, answering conceptually and procedurally important questions in the application of behavioral economic approaches to advance health: 1. How does the provision of simple, habit formation, and hybrid deposit sweepstake contracts conditional on statin adherence with daily reminders compare to just daily reminders in terms of adherence and improved LDL control during the intervention phase? 2. How does the provision of standard, habit formation, and hybrid sweepstake deposit contracts compare to just daily reminders in terms of achieving improved adherence and improved LDL control post- intervention? 3. How do these approaches compare in cost-effectiveness in improving LDL control? Patients will receive an active intervention for 6 months followed by 6 months of observation without incentives or other intervention to examine sustainability post-intervention. The primary outcome will be change in LDL cholesterol from baseline to 12 months. Incentives for patients will be structured as an adherence-based sweepstake with an expected value of $2.80, described in §B.3.d.v.e. Study sites. The study will be run by faculty and staff based at the LDI CHIBE and participants will be recruited among CVS Caremark members across the United States. CVS Caremark has approximately 40 million members; the number who are on statins and have

diabetes or CVD and MPR80% is more than sufficient for our study. We are working with CVS Caremark not only because they have a sufficiently large population of potentially eligible participants but because testing this type of approach in partnership with them dramatically increases the likelihood of the research being translated into practice. The study will be run by UPENN investigators, who will communicate with participants through the Way to Health portal, by phone, and by email. Inclusion criteria. Individuals at high risk of a cardiac event, specifically one of the following: -Individuals with clinical CVD (defined as diagnosis with myocardial infarction, stroke, or peripheral vascular disease) with an LDL greater than or equal to 100 mg/dl ; -Individuals with Diabetes (between the ages of 40-75) with an LDL greater than or equal to 100 mg/dl; -Individuals without clinical CVD or diabetes with LDL greater than or equal to 100 mg/dl and estimated 10-year CVD risk 7.5%; -Individuals without clinical CVD or diabetes with LDL cholesterol greater than or equal to 190 mg/dl A prescription filled for a statin medication within the last 12 months (derived from pharmacy records); Imperfect statin adherence level as defined by one of the following: -Medication Possession Ratio (MPR) less than or equal to 80% -A score 0 on the 8-item Morisky Medication Adherence Questionnaire completed during enrollment Exclusion criteria. Patients will be excluded if they have a known allergy or history of side effects to statins, are younger than 18 years old (see Protection of Human Subjects), will not or cannot give consent, are currently participants in another experimental study, have a markedly shortened life expectancy (diagnosis of metastatic cancer, or dementia), active or progressive liver disease, or ALT or AST 3x normal. Study procedures: Recruitment. Potential participants will be identified by reviewing CVS Caremark pharmacy claims records using our eligibility criteria of statin use and MPR less than or equal to 80%. CVS Caremark will facilitate communication to these members using existing communication channels (email, mail) by forwarding recruitment materials created by Penn. Potentially eligible individuals will be invited to visit the WTH portal or to call our study staff to enroll. Baseline pre-treatment assessment for patients. Potentially eligible patients will be sent letters inviting them to participate. Patients interested in enrolling who agree to provide written consent (See Protection of Human Subjects) will complete an intake form and consent using our Way to Health web portal. Scheduling of baseline LDL and ensuring LDL greater than or equal to 100 (if a diagnosis of CVD or diabetes) or LDL greater than 190 for non-diabetic or CVD individuals will be the final step in confirming eligibility.. Penn Medicine patients will receive a follow-up phone call from a study coordinator one week after the recruitment letter is sent to them. At this time, the coordinator will describe the study to the participant and if interested, will offer to begin enrollment over the phone. The participant will verbally complete the screening survey with the coordinator recording their answers in Way to Health. The coordinator will then go over the consent form with the participant and ask if they agree to take part in the study. If they agree to participate the participant will be mailed a copy of the consent form. The participant will then be asked to log on to the platform to complete the baseline survey before being fully enrolled into the study. This ensures that the participant has internet access and will be able to engage with the platform throughout the study. Additionally, we will incorporate a sweepstakes to our recruitment process. Participants can enter a sweepstake by completing and submitting their profile. They do not need to participate in the trial and participating in the trial will not change the likelihood of winning the prize Randomization. Randomization of patients to one of the 4 study arms will be performed through the Way to Health platform. Randomization will be stratified by employer, if more than one employer is involved, and will use block randomization with variable block sizes. After confirmation of patient eligibility, research staff will notify each patient participant of assignment using their preferred means of communication (text message, email, phone) and ask for confirmation of receipt. Participants will be given detailed instructions via Way to Health, phone or by mail for the arm of the study to which they have been assigned and patients in all arms will be given the GlowCaps and instructions on use. Scheduling. Direct low density lipoprotein (LDL) and a full lipid panel will be performed for each participant at baseline, 6 months, 12 months and possibly 18 months. The amount of blood drawn at baseline will be 3 ML and the amount drawn at 6 and 12 months will be 1 mL. Surveys will be completed at baseline, 6, and 12 months. Participants will return GlowCaps at the end of the 6-month intervention. The Way to Health participant tracking system will automatically remind the study coordinators that each enrolled subject has a scheduled follow-up visit at the end of months 6, 12 and possibly 18. We will obtain extensive contact information from each participant and update it at each follow-up visit. We will call participants who miss follow-up visits weekly for 4 weeks and send 2 letters during these 4 weeks. If any participants appear lost to follow-up, we will call their primary physician to ascertain their status. To enhance retention, those who enroll in the study will be compensated $25 for baseline labs (if they require a lab test), $25 if they are eligible and start in the study, and $75 for tests and 6 and 12 months, for a total payment of $200 per participant. Patients who undergo eligibility screening but do not enroll in the study will be compensated $25 for their time. Generous participation incentives have succeeded

in minimizing differential drop out in our previous studies. Implementation of intervention arms. For each of the incentive arms, eligibility for an incentive will be based on daily statin adherence as described in Section B.3.d.i but the details of the incentive design implementation varies as described below. Standard sweepstake incentive. At study entry, we will assign each participant a two-digit number. Each day GlowCaps will automatically upload adherence data for the previous day via the internet to a secure server housed at UPENN. The Way to Health system generates two-digit random daily sweepstake numbers and compares them electronically to the participants two-digit identification number to determine eligibility for awards. In the standard sweepstake arm, if the two digit number matches, which will happen about 1 in 100 times, the participant will be eligible to win $100 if s/he was adherent the day before. If the two digit number does not match, but either the first digit or second digit matches in the right place, the subject is eligible to win $10, which will happen about 1 in 5 times (more precisely, 18 in 100 times). The expected value of this sweepstake is $2.80/day such that total winnings per 6 month for a fully adherent participant have an expected value of $512.40 (but could be more or less for an individual participant depending upon chance). As in our previous studies, we have designed the sqweepstake-based incentives so that, each day, adherent participants receive rapid feedback about whether they won, and non-adherent participants receive rapid feedback about whether they would have won had they been adherent. This program incorporates key aspects of optimal design, including objective and reliable confirmation of behavior change at frequent intervals, large potential payments to reinforce the target behavior, frequent reinforcement using smaller payments, and the use of anticipated regret, a powerful motivator. We set the expected value of the sweepstake at about $3/day based on our success in significantly affecting weight loss and medication adherence with these parameters. In addition, there is evidence that a patient with established CVD has health expenditures ranging from $18,000-$30,000 per year, suggesting that incentives of $3 a day, if effective, could be cost effective due to the potential savings. Information on whether the participant won will be electronically transmitted each day by text message, phone, or email (patient choice) and will also be available on the patients page on the Way to Health portal. Habit formation incentive: Same as in the standard sweepstake except that participant will receive half of the daily incentive if they do not take their statin before receiving a reminder at 9pm (the exact time can be altered per patient preference). Hybrid sweepstake and Deposit contract: Same as in the standard sweepstake whereby patients automatically enroll into a daily sweepstake when being adherent except that the sweepstakes is only worth half of the amount. Participants in this arm will receive $5 for matching one number and $50 for matching two numbers. Additionally, $45 will be placed in a deposit account until the end of the month and will decrease by $1.50 every day the participant is non-adherent. Participants will receive the balance in their deposit account at the end of each month. When the next month begins, the deposit account re-sets to $45. Measures LDL cholesterol (primary outcome). The evidence base linking improvements in LDL cholesterol to reductions in CVD is extensive, supporting about a 20% reduction in CVD per 40 mg/dl reduction. LDL cholesterol is easily monitored through a simple blood test. The primary outcome will be change in LDL between baseline (prior to randomization) and 12 months. Adherence measured using Vitality GlowCaps, Medsignals, [redacted] Wisepill (secondary outcome). Several methods have been used to measure adherence; no gold standard exists. There are multiple limitations to methods such as patient self-report and pill counts. GlowCaps provides an unbiased assessment of pill bottle opening and a valid approach to verifying self-administered pill taking, reflecting not only daily use but also patterns of drug use and timing. This method assumes that each time the cap is opened, a dose is taken, and that doses are not taken when the cap is not opened. GlowCaps are just like regular pill bottles so there is little need for patients to decant pills into other containersa process that can lead to false negative measures of adherence. Similarly, although it is possible for patients to open a pill bottle but not take their medication, evidence suggests that once an individual opens a pill bottle, pills are nearly always taken and numerous studies have established the validity of electronic pill container measures. Each day the GlowCap will electronically transmit whether a participant opened his/her prescription bottle to take his/her statin via a built-in modem to the central server (there is no internet charge to participants) and a simple wireless device plugged into an outlet. Participants will be considered adherent only if we receive electronic notification signaling that the statin bottle was opened once the previous day. GlowCaps and the wireless transmitters are easily portable and can be used while traveling. Each patient will receive instructions to call the study nurse for any changes in dose frequency (an unlikely event in the context of statins, all of which are recommended as once-a-day medications), in which case the GlowCap will be reprogrammed. Statin adherence assessed through the Medication Possession Ratio (MPR, secondary outcome) Because all participants will have pharmacy benefits through CVS-Caremark, we will also have data about when patients filled statin prescriptions and number of statin pills that the patients received. The MPR is calculated as the number of days for which a medication is supplied (numerator, consisting of number

of statin pills provided by CVS to the participant) separately for the six month intervention and the six month post-intervention period. The MPR will provide a secondary means to compare statin adherence across each arm. Limitations to analyzing the MPR data include the assumption that patients may receive their statin medication but not take the pills, and that, if prescribing physicians lower the statin dose, adherent patients may continue to use their existing statin pills (e.g. cutting a 40mg tablet into 20 mg half-tablets and refill their medications at a longer interval, creating a falsely low MPR. Potential confounders and mediators. Although this randomized trial is designed to balance all factors that could alter LDL levels (other than the interventions), we will measure potential residual confounders at baseline and adjust for them in later analyses. Many of these variables may also serve as either moderators (factors that predict which people are helped by the intervention) or mediators (variables related to mechanisms whereby the intervention works) in the intervention-outcome pathway. We will have information on patient demographics, socioeconomic status, comorbidities, and baseline LDL. Method of data collection. Baseline data will be collected via the web using standardized data collection forms modeled after data collection instruments used in our previous studies. Baseline data will include detailed demographics (e.g., age, sex, race/ethnicity, income, education, marital status, employment, health insurance). Other variables to be collected include: (1) Risk perceptions measured using a visual analog scale; (2) Numeracy using pre-validated measures of numeracy; and (3) Health status measured using the SF-12 to assess health-related quality of life and the Health Utility Index (HUI) to assess health preferences. At the 12-month visit, participants will be surveyed about all variables that may change, such as health status.

The following documents are currently attached to this item:

There are no documents attached for this item.

Deception
Does your project use deception?
No

International Research
Are you conducting research outside of the United States?
No

Analysis Plan
Data analysis plans. Prior to analysis, we will produce data summaries including graphical methods to assess data quality, examine central tendencies and distributional assumptions and randomization balance. The primary analysis will consist of unadjusted intent-to-treat hypothesis tests for the significance of coefficients associated with treatment assignment in linear models of change in LDL between baseline and 12 months. We will also estimate regression models adjusted for covariates of interest (such as patient sex, income, race, baseline LDL, and study site) which may have imbalances across groups despite the randomization. We will assess interaction terms between the a priori potential effect modifiers such as study site, income level, race, and baseline LDL; these will be largely exploratory. We will also fit longitudinal models of the LDL levels at baseline, 6 months, 12 months, and possibly 18 months and assess whether the changes in LDL over time are different between the first 6 months and the second-third 6 months; this can be accomplished using piecewise linear regression with a knot at 6 months to allow the rate of change over time to differ at that point. We can test whether these differences are statistically significant, and assess using model diagnostics whether linear trends with time are appropriate. Interaction terms between the time variables and treatment arm will indicate whether some groups have more rapid declines in LDL. All hypothesis tests will be two-sided and use adjusted Type I error rates as described above to maintain control of false positive test results. Models will be assessed using standard diagnostic techniques. We will assess the normality of the outcome and use transformations to improve the approximation if necessary or robust regression techniques if suitable transformations cannot be found. Handling of missing data is an important issue in all RCTs. Follow-up data will be missing if participants miss visits and do not have labs taken. We anticipate low levels of loss to follow-up, but will conservatively assume that these patients fail to achieve any reduction in LDL and are non-adherent. We will compare dropout rates by arm, and will attempt to find the reasons for missing data and will compare baseline characteristics in participants with complete vs. incomplete follow-up. In secondary analyses we will investigate the sensitivity to modeling assumptions using imputation models and inverse-probability-weighted estimating equations and models that adjust for informative missing data. The analyses for secondary outcomes in Aims 1, 2, and

3 will parallel those for the primary outcome. As part of the analysis plan, the research team will pull additional existing LDL data if it is available in the Penn Medicine EMR, for only the Penn Medicine subjects enrolled in the study, up to a 36-month time point from the date of enrollment for each subject. There are many advantages to looking at our subjects LDL data to further advance knowledge that will be valuable to the practice of medicine among patients with adherence problems. In addition, given this study is focusing on the question of habit formation and long-term effects from a time limited intervention, society may benefit to a much greater extent having these additional data. There is no risk to subjects because these data have already been collected and in the EMR system. We are eager to learn as much as we can from the subjects participation in the study and we appreciate your consideration in an effort to move our analysis forward. The research team will also ascertain if there is a deceased status in the subjects record, as this will be important to know for our analysis. In addition, the research team will pull additional existing LDL data and demographic data (deceased, age, gender, race/ethnicity) if it is available in the Penn Medicine EMR, for patients we have identified as eligible for the study but who did not enroll in the study, to supplement the primary analysis results. Sensitivity Analyses: Between November 2015 and July 2016 the device failed at rates that exceeded rates obtained in our earlier studies. We anticipated that this might increase the variance of the primary outcome, with some subjects perhaps responding positively with increased compliance to statin medication and some negatively with decreased compliance. Positive responses might reflect increased intervention from study staff, or the swapping out of one device for another spurring increased compliance. Negative responses might reflect frustration, particularly on the Process arm or the Process & Outcome arm, with the inconsistent devices. We note that our completion rates for lab visits are close to our expected rates of 85%, suggesting that the device issue has not had an impact on subject retention. Sensitivity Analyses, Device Failure: While the primary analysis is intent to treat, we will characterize our subjects in a number of ways to explore possible impacts of the period with higher rates of device failure. Our study is not powered for hypothesis tests of these effects, and thus the analysis will be exploratory. The following analyses will be conducted: 1. We will stratify the primary analysis by whether a subject experienced two or more weeks of time in the window of time when the devices failed at rates higher than previous studies. Estimates and 95% CI of the effect sizes for the two strata will be determined with the goal of determining whether is evidence of a reduction in effect size due to an excess of faulty devices. 2. For each subject we will create a variable quantifying the proportion of their treatment time in the window with properly functioning devices. For most subjects, this variable will be 1, but for a subjects with say 2 months in the window of faulty devices, this would be 61/356=.83. We will additionally include a variable indicating whether a subject required a device swap. These variables will be included in the primary regression model. Of interest is whether the LDL levels at 12 months (change from baseline) are associated with either variable, and whether the association between LDL level at 12 months and treatment group is confounded by either variable. Additionally, we anticipate that any effect of device malfunction should primary affect the response of subjects on the Process or the Process & Outcome arms. We will thus ¬¬repeat our analyses separately with these two arms. 3. We will use a logistic regression to determine whether the likelihood of completing the study (lab visit at 12 months) is associated with the proportion of time with a properly functioning device, or the occurrence of a device swap. 4. We will explore patterns of adherence over time as a function of device type. Unfortunately having a faulty device means that we do not have reliable adherence data during the period of device failure. Thus questions will be explored using regression models with data from the periods when the devices were functioning normally. These include: a. Do mean rates of compliance differ by device for subjects randomized to a single device for the entire study period. Does the decline over time in compliance differ by device for subjects randomized to a single device over the study period. b. On average, during the period when our devices returned to normal functioning, are compliance rates similar for subjects who were exposed to faulty devices versus subjects who had normally functioning devices. These will be GEE models with a binary outcome for each day (compliant or not). The predictors include time on study, an indicator variable of whether the subject was on study during the period when the devices failed (or a quantitative variable indicating proportion of time with a functioning device), and an indicator variable for whether subjects experienced more than one device. The analysis will be carried out for the study as a whole (including a treatment indicator), and for each treatment group. Qualitative Study To improve the design of future interventions, we will engage in a post-study qualitative process evaluation to better understand why some study participants succeed in changing behavior and others do not, and what elements of the approach were acceptable to participants. Patient Interviews The proposed qualitative study will consist of semi-structured phone interviews, meaning there is a conversational component to it. The sample size will consist of 60 (15 per arm) participants who were the least and most successful in improving LDL. Likely, saturation will be achieved with 10-15 interviews per arm. The participants will be offered $50

for completing the post-study phone interview. Examples of topics that will guide full script development include: motivations for enrolling, perceived benefits and barriers to participation, and the impact of financial incentives. Procedures Research personnel will contact patient subjects by phone and ask whether they would like to participate in a post-study phone interview. Research personnel will follow a detailed phone script including reviewing a written statement of research before the interview begins, which explains the elements of the interview and processes for data confidentiality. Participants will be informed that they can choose not to participate in any element of the interview (including audio recording) without penalty. Verbal consent will be obtained prior to conducting the phone interview. The research personnel will then conduct the phone interview. The phone interviews are expected to last approximately 30 minutes. All audio is only stored for set periods of time and then purged completely from the digital recorders. Analysis All phone interviews will be digitally recorded and sent to a transcription service (ADA Transcription) to be transcribed. ADA Transcription is a transcription agency located in Mount Holly, NJ. (http://www.adatranscription.com/). Identifying patient information will be de-identified prior to sending to ADA Transcription. The purpose of the analysis will be to extract themes and narratives relevant to the research questions. Audio recordings of the interviews will be uploaded to ADA Transcription's website. ADA Transcription uses a file transfer program called Citrix Sharefile. All communications between Citrix ShareFile and the user are encrypted using either Secure SocketLayer (SSL) or Transport Layer Security (TLS) encryption protocols and up to AES 256-bit encryption, a level of encryption that is similar to what banks use (which is higher than most medical facilities). The data will be encrypted during uploads and downloads, and ShareFile also encrypts stored files when they are at rest on our servers for an additional layer of security. ADA password protects all audio files and can track users' access to the data. All audio is only stored for set periods of time and then purged completely from the system. Transcripts are returned to the research personnel in password-protected Word files via email. Audio recordings will be downloaded and transcribed with the recordings themselves subsequently erased at the completion of the study to avoid audible identification of participants. The transcripts will be coded qualitatively for thematic and content analysis and synthesized for peer-reviewed publication. The analysis will be mostly qualitative, with descriptive statistics used to summarize categories of data that arise (such as demographics). Data Management All interview notes and audio records will be stored under encryption without use of patient identifiers. Audio recording will be conducted with Digital Device Recorders (DDR). Files will be stored on computers located in locked offices, and after transfer, the DDRs will be wiped of content. The DDRs will be stored in the previously described locked office. Additionally, the name of the subject being interviewed will never be stored on the DDR, only a study number will be used. REDCap will be used to develop and manage the study database, and only approved study personnel will have access to records. Subjects can withdraw and request disposal of their records at any time. ADA Transcription will be used to ensure HIPAA compliant professional transcription. Subject Confidentiality Precautions are in place to ensure the data is secure by using passwords and encryption. The research personnel will use the subjects' existing study identification number to identify all subject study data in research databases. Once the interviews are completed, no personally identifiable information will be associated with participant's responses or their data. In addition to these measures, all information that is collected as part of this study will not be shared with other groups or investigators who are part of the research team, except as required by the Institutional Review Board for the protection of human subjects. Subject Privacy Each participant in the study has already been assigned a unique study ID number. The link between name and ID number will be kept in the study's existing RedCap database that is accessible only to the key study personnel. Names of participants will not be included on the transcripts that derive from the interviews. The audio copies of the interviews will be kept in a locked drawer of the locked office of a member of the research team. The recordings will not be shared with anyone outside the research team. We will take extensive precautions to protect the privacy of subjects. A key containing information will be kept in locked file cabinets until study interviews are completed and the data have been checked for completeness and accuracy. Consent Process Overview Prior to participation in the post-study interviews, all participants will be asked to provide verbal consent. The interview script will be read aloud by the individual conducting the interview. It will be made clear to all subjects that all information will be kept confidential, and that their participation is entirely voluntary, and they are allowed to leave or withdraw consent at any time. Potential Study Risks There are minimal risks involved in participating in the phone interviews. There is a slight risk of potential breaches of confidentiality for subjects participating in the phone interviews. Regarding the possibility of confidentiality loss, the collection of subject identifiers will be minimized, and any identifiers will be eliminated in transcripts and other electronic documents. Audio recordings and physical documentation will be kept locked away until their destruction after transcription. Every effort will be made to maintain subject privacy and confidentiality. This qualitative study has the potential to provide a strong

and broad benefit to society through informing future interventions to enhance patient outcomes. Potential Study Benefits From the perspective of those interviewed, there are few individual benefits from participating in the interviews than being given an opportunity to voice their personal experiences and opinions about participating in the study. Interview participants might also benefit from feeling that their efforts will affect positive change in patient health outcomes. Qualitative Study To improve the design of future interventions, we will engage in a post-study qualitative process evaluation to better understand why some study participants succeed in changing behavior and others do not, and what elements of the approach were acceptable to participants. Patient Interviews The proposed qualitative study will consist of semi-structured phone interviews, meaning there is a conversational component to it. The sample size will consist of 60 (15 per arm) participants who were the least and most successful in improving LDL. Likely, saturation will be achieved with 10-15 interviews per arm. The participants will be offered $50 for completing the post-study phone interview. Examples of topics that will guide full script development include: motivations for enrolling, perceived benefits and barriers to participation, and the impact of financial incentives. Procedures Research personnel will contact patient subjects by phone and ask whether they would like to participate in a post-study phone interview. Research personnel will follow a detailed phone script including reviewing a written statement of research before the interview begins, which explains the elements of the interview and processes for data confidentiality. Participants will be informed that they can choose not to participate in any element of the interview (including audio recording) without penalty. Verbal consent will be obtained prior to conducting the phone interview. The research personnel will then conduct the phone interview. The phone interviews are expected to last approximately 30 minutes. All audio is only stored for set periods of time and then purged completely from the digital recorders. Analysis All phone interviews will be digitally recorded and sent to a transcription service (ADA Transcription) to be transcribed. ADA Transcription is a transcription agency located in Mount Holly, NJ. (http://www.adatranscription.com/). Identifying patient information will be de-identified prior to sending to ADA Transcription. The purpose of the analysis will be to extract themes and narratives relevant to the research questions. Audio recordings of the interviews will be uploaded to ADA Transcription's website. ADA Transcription uses a file transfer program called Citrix Sharefile. All communications between Citrix ShareFile and the user are encrypted using either Secure SocketLayer (SSL) or Transport Layer Security (TLS) encryption protocols and up to AES 256-bit encryption, a level of encryption that is similar to what banks use (which is higher than most medical facilities). The data will be encrypted during uploads and downloads, and ShareFile also encrypts stored files when they are at rest on our servers for an additional layer of security. ADA password protects all audio files and can track users' access to the data. All audio is only stored for set periods of time and then purged completely from the system. Transcripts are returned to the research personnel in password-protected Word files via email. Audio recordings will be downloaded and transcribed with the recordings themselves subsequently erased at the completion of the study to avoid audible identification of participants. The transcripts will be coded qualitatively for thematic and content analysis and synthesized for peer-reviewed publication. The analysis will be mostly qualitative, with descriptive statistics used to summarize categories of data that arise (such as demographics). Data Management All interview notes and audio records will be stored under encryption without use of patient identifiers. Audio recording will be conducted with Digital Device Recorders (DDR). Files will be stored on computers located in locked offices, and after transfer, the DDRs will be wiped of content. The DDRs will be stored in the previously described locked office. Additionally, the name of the subject being interviewed will never be stored on the DDR, only a study number will be used. REDCap will be used to develop and manage the study database, and only approved study personnel will have access to records. Subjects can withdraw and request disposal of their records at any time. ADA Transcription will be used to ensure HIPAA compliant professional transcription. Subject Confidentiality Precautions are in place to ensure the data is secure by using passwords and encryption. The research personnel will use the subjects' existing study identification number to identify all subject study data in research databases. Once the interviews are completed, no personally identifiable information will be associated with participant's responses or their data. In addition to these measures, all information that is collected as part of this study will not be shared with other groups or investigators who are part of the research team, except as required by the Institutional Review Board for the protection of human subjects. Subject Privacy Each participant in the study has already been assigned a unique study ID number. The link between name and ID number will be kept in the study's existing RedCap database that is accessible only to the key study personnel. Names of participants will not be included on the transcripts that derive from the interviews. The audio copies of the interviews will be kept in a locked drawer of the locked office of a member of the research team. The recordings will not be shared with anyone outside the research team. We will take extensive precautions to protect the privacy of subjects. A key containing information will be kept in locked file

cabinets until study interviews are completed and the data have been checked for completeness and accuracy. Consent Process Overview Prior to participation in the post-study interviews, all participants will be asked to provide verbal consent. The interview script will be read aloud by the individual conducting the interview. It will be made clear to all subjects that all information will be kept confidential, and that their participation is entirely voluntary, and they are allowed to leave or withdraw consent at any time. Potential Study Risks There are minimal risks involved in participating in the phone interviews. There is a slight risk of potential breaches of confidentiality for subjects participating in the phone interviews. Regarding the possibility of confidentiality loss, the collection of subject identifiers will be minimized, and any identifiers will be eliminated in transcripts and other electronic documents. Audio recordings and physical documentation will be kept locked away until their destruction after transcription. Every effort will be made to maintain subject privacy and confidentiality. This qualitative study has the potential to provide a strong and broad benefit to society through informing future interventions to enhance patient outcomes. Potential Study Benefits From the perspective of those interviewed, there are few individual benefits from participating in the interviews than being given an opportunity to voice their personal experiences and opinions about participating in the study. Interview participants might also benefit from feeling that their efforts will affect positive change in patient health outcomes. Cost effectiveness analysis. Measurement of costs and cost effectiveness. For our initial analysis, we will take a payer perspective. We will complete a within-trial analysis comparing incremental costs and incremental change in LDL in each arm, compared with control, during the 12 months of the trial. As a secondary analysis, we will substitute a societal perspective, which will include costs to patients, such as transportation and the time the patient devotes to the intervention. For both these analyses, costs will include: (1) incentive payments to participants (excluding incentives specific to the research); (2) operational costs of implementing the interventions, including staff time administering the incentives and the computer platform to deliver the incentives (the Way to Health platform used in this study or an alternative that might be used by an employer); and (3) costs of the adherence measurement device (the electronic pill bottle and fees for connecting to the computer platform). We will also include the drug costs associated with statin medication use when adherence improves. Following usual practice in cost-effectiveness analysis we will conduct sensitivity analyses to assess the impact of uncertainty about these data, e.g., the standard error of the estimate of effectiveness, and to explore variations that might occur across employers. For the societal perspective cost-effectiveness analysis, we will follow the recommendations of the Second Panel on Cost-Effectiveness in Health and Medicine to compute additional costs to patients related to participating in an incentives program, including medication side effects, and time spent on the intervention, including travel and waiting time (using data from the American Time Use Survey); time will be valued at appropriate wage rates.1 Long-term Effects of LDL Reductions on CVD Events and Cost-Effectiveness: The goal of lowering LDL is to prevent CVD complications. To estimate the cost and effectiveness associated with preventing cardiac outcomes, if our primary analyses of effectiveness indicate significant effects of the intervention, we will use a validated model of long-term cardiac risk, the Coronary Heart Disease (CHD) Policy Model. The CHD Policy Model is a computer-simulation, state-transition (Markov cohort) model of the incidence and prevalence of CVD (myocardial infarction, sudden death, revascularization, angina, stroke) and of the mortality and costs associated with these conditions in U.S. adults.62 The Model has been used to describe trends in CVD, project the effects of interventions to reduce CVD risk, and model the cost-effectiveness of interventions.2,3,4-5 Dr. Bibbins-Domingo, who leads the core modeling team, will adapt the Model to our study population, individuals with diabetes and/or known CVD. Based on decreases in LDL observed in the arms of this study, the Model will estimate the number and type of CVD events avoided, the quality-adjusted life years (QALYs) gained, and CVD treatment costs in each arm. We will use these estimates to calculate the cost-effectiveness of the intervention in each arm. Costs and health effects will be discounted at 3% per annum. Return on investment: In addition to the cost-effectiveness analysis, if our primary analyses of effectiveness indicate significant effects of the intervention, we will conduct an analysis of potential return on investment for employers, the business case for preventing expensive health complications in the short- to medium-term. Using the CHD Policy Model we will estimate costs and savings of an incentives plan to the employer 3, 5, and 7 years after implementation. Savings will include reduced medical expenditures due to avoided CVD events and complications as well as increases in projected productivity as a result of reductions in CVD disability and death.6 Cost-effectiveness References 1. Peter J. Neumann, Gillian D. Sanders, Louise B. Russell, Joanna E. Siegel, Theodore G. Ganiats, editors, Cost-Effectiveness in Health and Medicine, second edition, New York: Oxford University Press, 2016. 2.Bibbins-Domingo K, Coxson P, Pletcher MJ, Lightwood J, Goldman L. Adolescent overweight and future adult coronary heart disease. N Engl J Med 2007;357:2371-9. 3.Odden MC, Coxson PG, Moran A, Lightwood JM, Goldman L, Bibbins-Domingo K. The impact of the aging population on

coronary heart disease in the United States. Am J Med 2011;124:827-33 e5. 4. Lazar LD, Pletcher MJ, Coxson PG, Bibbins-Domingo K, Goldman L. Cost-effectiveness of statin therapy for primary prevention in a low-cost statin era. Circulation 2011;124:146-53. 5.Pletcher MJ, Lazar L, Bibbins-Domingo K, et al. Comparing impact and cost-effectiveness of primary prevention strategies for lipid-lowering. Ann Intern Med 2009;150:243-54. 6. Lightwood J, Bibbins-Domingo K, Coxson P, Wang YC, Williams L, Goldman L. Forecasting the future economic burden of current adolescent overweight: an estimate of the coronary heart disease policy model. Am J Public Health 2009;99:2230-7.

The following documents are currently attached to this item:

There are no documents attached for this item.

Data confidentiality

x **Paper-based records will be kept in a secure location and only be accessible to personnel involved in the study.**

x **Computer-based files will only be made available to personnel involved in the study through the use of access privileges and passwords.**

Prior to access to any study-related information, personnel will be required to sign statements agreeing to protect the security and confidentiality of identifiable information.

x **Wherever feasible, identifiers will be removed from study-related information.**

A Certificate of Confidentiality will be obtained, because the research could place the subject at risk of criminal or civil liability or cause damage to the subject's financial standing, employability, or liability.

A waiver of documentation of consent is being requested, because the only link between the subject and the study would be the consent document and the primary risk is a breach of confidentiality. (This is not an option for FDA-regulated research.)

x **Precautions are in place to ensure the data is secure by using passwords and encryption, because the research involves web-based surveys.**

Audio and/or video recordings will be transcribed and then destroyed to eliminate audible identification of subjects.

Subject Confidentiality

Study participants will be recruited by Penn Medicine or CVS Caremark on behalf of the health plans as a business associate. As a PBM, CVS Caremark provides services for and maintains data on approximately 40 million members of employer-sponsored health plans across the United States. CVS Caremark will identify potential participants by reviewing the pharmacy claims records it holds as a PBM using our eligibility criteria of statin use and MPR80%. The pharmacy claims records are PHI governed by the HIPAA Privacy Rule. CVS Caremark holds the pharmacy claims data in its role as a PBM and business associate to employer-sponsored health plans. As a result, CVS Caremark must obtain the consent of the health plans prior to using the pharmacy claims records for these recruitment purposes. Further, CVS Caremark must be authorized by its business associate agreement with the health plans to conduct the preparatory to research activities. As required by HIPAA to conduct preparatory to research activities, CVS Caremark, as a researcher and business associate of the employer-sponsored health plans, must provide representations to the health plans that (i) the use or disclosure is sought solely to review PHI as necessary for purposes preparatory to research, i.e., identifying potential study participants; (ii) CVS Caremark will not remove any PHI from the health plans in the course of the review, i.e., CVS Caremark will only access PHI maintained on behalf of the health plans; and (iii) the PHI for which use or access is sought is necessary for the research purposes. The receipt of such assurances will satisfy the health plans' obligation to only permit uses and disclosures of the PHI of their members as authorized under HIPAA. CVS Caremark will take the following steps so that its access to and use of the PHI will involve no more than a minimal risk to the privacy of health plan members: First of all, steps that will be taken to protect the PHI while it is being accessed and analyzed to identify eligible study participants. CVS Caremark will only permit a very limited number of its personnel to access the PHI to conduct the analysis. All such personnel will be trained on HIPAA-compliance, including that they cannot further use or disclose the PHI for any purposes except for the specific study recruitment activities. CVS Caremark will apply its standard security policies, procedures and measures to protect the security of the data. The PHI will be encrypted

and stored on CVS Caremarks password protected SASNODE server. Steps will also be taken to protect the PHI when CVS Caremark communicates to the potential study participants about enrolling in the study. Such steps include making sure that any written materials are sent in a manner that does not unnecessarily disclose the individual's diagnosis or status. If written materials are being sent in the mail, they will be enclosed in an envelope and e-mails will only be sent if it would otherwise be appropriate to send PHI to that individual via e-mail. Personnel, including vendors, who will be making the communications will also be trained on HIPAA-compliance, including that they cannot further use or disclose the PHI for any purposes except for the specific study recruitment activities. CVS Caremark personnel who will be conducting the recruitment activities will destroy the identifiers at the earliest opportunity consistent with conduct of the research, unless there is a health or research justification for retaining the identifiers or such retention is otherwise required by law. As a Business Associate, CVS Caremark is prohibited by both HIPAA and by its business associate agreements with the health plans to not reuse or disclose the PHI to any other person or entity, except as required by law or as otherwise permitted under HIPAA. In addition, the research agreement between CVS Caremark and UPenn also prohibits CVS Caremark from using or disclose the PHI except as required by law or other permitted under HIPAA. Upon identification of potentially eligible participants, CVS Caremark, as a business associate on behalf of the health plans, will communicate recruitment materials created by Penn and will do so using existing communication channels it has with such individuals, consistent with the measures identified above to protect the privacy of the individuals. Potentially eligible individuals will be invited to visit the WTH portal or to call our study staff to enroll. Individuals interested in enrolling who agree to provide written consent (See Protection of Human Subjects) will complete an intake form and consent using our Way to Health web portal. CVS Caremark will not disclose or otherwise provide any PHI to UPenn until it receives evidence of executed written consent forms containing valid HIPAA-compliant language authorizing CVS Caremark to do so. Research material will be obtained from participant interviews, the GlowCaps adherence monitoring devices, and laboratory data from in-person blood draws for cholesterol lipid panel (including LDL). All participants will provide informed consent for access to these materials. The data to be collected include demographic data (e.g., age, sex, self-identified race), outcome data, adherence data (from the GlowCaps), and psychosocial measures (e.g., SF-12), and medical conditions and medications. Research material that is obtained will be used for research purposes only. The same procedure used for the analysis of automated data sources to ensure protection of patient information will be used for the survey data, in that patient identifiers will be used only for linkage purposes or to contact patients. The study identification number, and not other identifying information, will be used on all data collection instruments. All study staff will be reminded to appreciate the confidential nature of the data collected and contained in these databases. The UPENN Biomedical Informatics Consortium (BMIC) will be the hub for the hardware and database infrastructure that will support the project and where the Way to Health web portal is based. The BMIC is a joint effort of the University of Pennsylvania's Abramson Cancer Center, the Cardiovascular Institute, the Department of Pathology, and the Leonard Davis Institute. The BMIC provides a secure computing environment for a large volume of highly sensitive data, including clinical, genetic, socioeconomic, and financial information. Among the IT projects currently managed by BMIC are: (1) the capture and organization of complex, longitudinal clinical data via web and clinical applications portals from cancer patients enrolled in clinical trials; (2) the integration of genetic array databases and clinical data obtained from patients with cardiovascular disease; (3) computational biology and cytometry database management and analyses; (4) economic and health policy research using Medicare claims from over 40 million Medicare beneficiaries. BMIC requires all users of data or applications on BMIC servers to complete a BMIC-hosted cybersecurity awareness course annually, which stresses federal data security policies under data use agreements with the university. Curriculum includes HIPAA training and covers secure data transfer, passwords, computer security habits and knowledge of what constitutes misuse or inappropriate use of the server. We will implement multiple, redundant protective measures to guarantee the privacy and security of the participant data. All investigators and research staff with direct access to the identifiable data will be required to undergo annual responsible conduct of research, cybersecurity, and Health Insurance Portability and Accountability Act certification in accordance with University of Pennsylvania regulations. All data for this project will be stored on the secure/firewalled servers of the BMIC Data Center, in data files that will be protected by multiple password layers. These data servers are maintained in a guarded facility behind several locked doors, with very limited physical access rights. They are also cyber-protected by extensive firewalls and multiple layers of communication encryption. Electronic access rights are carefully controlled by University of Pennsylvania system managers. We will use highly secure methods of data encryption for all transactions involving participants financial information using a level of security comparable to what is used in commercial financial transactions. We believe this multi-layer system of data security,

identical to the system protecting the University of Pennsylvania Health Systems medical records, greatly minimizes the risk of loss of privacy. In addition, risk of loss of confidentiality will be minimized by storing completed paper copies of the surveys and signed informed consent forms in locked file cabinets in locked offices accessible only to trained study staff. Each subject will be assigned a unique identifier without identifying information, and data will be entered into an electronic database using only the unique identifier. Only trained study staff will have access to the code that links the unique identifier to the subjects identity. Electronic data will be stored on secure, password-protected firewalled servers at UPENN.

Sensitive Research Information*

Does this research involve collection of sensitive information about the subjects that should be excluded from the electronic medical record?

No

Subject Privacy

Privacy refers to the person's desire to control access of others to themselves. Privacy concerns people, whereas confidentiality concerns data. Describe the strategies to protect privacy giving consideration to the following: The degree to which privacy can be expected in the proposed research and the safeguards that will be put into place to respect those boundaries. The methods used to identify and contact potential participants. The settings in which an individual will be interacting with an investigator. The privacy guidelines developed by relevant professions, professional associations and scholarly disciplines (e.g., psychiatry, genetic counseling, oral history, anthropology, psychology).

Study participants will be recruited by Penn Medicine or CVS Caremark on behalf of the health plans as a business associate. As a PBM, CVS Caremark provides services for and maintains data on approximately 40 million members of employer-sponsored health plans across the United States. CVS Caremark will identify potential participants by reviewing the pharmacy claims records it holds as a PBM using our eligibility criteria of statin use and MPR80%. The pharmacy claims records are PHI governed by the HIPAA Privacy Rule. CVS Caremark holds the pharmacy claims data in its role as a PBM and business associate to employer-sponsored health plans. As a result, CVS Caremark must obtain the consent of the health plans prior to using the pharmacy claims records for these recruitment purposes. Further, CVS Caremark must be authorized by its business associate agreement with the health plans to conduct the preparatory to research activities As required by HIPAA to conduct preparatory to research activities, CVS Caremark, as a researcher and business associate of the employer-sponsored health plans, must provide representations to the health plans that (i) the use or disclosure is sought solely to review PHI as necessary for purposes preparatory to research, i.e., identifying potential study participants; (ii) CVS Caremark will not remove any PHI from the health plans in the course of the review, i.e., CVS Caremark will only access PHI maintained on behalf of the health plans; and (iii) the PHI for which use or access is sought is necessary for the research purposes. The receipt of such assurances will satisfy the health plans' obligation to only permit uses and disclosures of the PHI of their members as authorized under HIPAA. CVS Caremark will take the following steps so that its access to and use of the PHI will involve no more than a minimal risk to the privacy of health plan members: First of all, steps that will be taken to protect the PHI while it is being accessed and analyzed to identify eligible study participants. CVS Caremark will only permit a very limited number of its personnel to access the PHI to conduct the analysis. All such personnel will be trained on HIPAA-compliance, including that they cannot further use or disclose the PHI for any purposes except for the specific study recruitment activities. CVS Caremark will apply its standard security policies, procedures and measures to protect the security of the data. The PHI will be encrypted and stored on CVS Caremarks password protected SASNODE server. Steps will also be taken to protect the PHI when CVS Caremark communicates to the potential study participants about enrolling in the study. Such steps include making sure that any written materials are sent in a manner that does not unnecessarily disclose the individual's diagnosis or status. If written materials are being sent in the mail, they will be enclosed in an envelope and e-mails will only be sent if it would otherwise be appropriate to send PHI to that individual via e-mail. Personnel, including vendors, who will be making the communications will also be trained on HIPAA-compliance, including that they cannot further use or disclose the PHI for any purposes except for the specific study recruitment activities. CVS Caremark personnel who will be conducting the recruitment activities will destroy the identifiers at the earliest opportunity consistent with conduct of the research, unless there is a health or research justification for retaining the identifiers or such retention is otherwise required by law. As a Business Associate, CVS Caremark is prohibited by both HIPAA and by its business associate agreements with the health plans to not reuse or disclose the PHI to any other person or entity, except as required by law or as otherwise

permitted under HIPAA. In addition, the research agreement between CVS Caremark and UPenn also prohibits CVS Caremark from using or disclose the PHI except as required by law or other permitted under HIPAA. Upon identification of potentially eligible participants, CVS Caremark, as a business associate on behalf of the health plans, will communicate recruitment materials created by Penn and will do so using existing communication channels it has with such members, consistent with the measures identified above to protect the privacy of the individuals. Potentially eligible individuals will be invited to visit the WTH portal or to call our study staff to enroll. Individuals interested in enrolling who agree to provide written consent (See Protection of Human Subjects) will complete an intake form and consent using our Way to Health web portal. CVS Caremark will not disclose or otherwise provide any PHI to UPenn until it receives evidence of executed written consent forms containing valid HIPAA-compliant language authorizing CVS Caremark to do so. Interested employees go to the study website to provide informed consent. These individuals will have the option of entering data related to eligibility and their demographic and clinical characteristics through the Way to health internet portal or by phone. Each potential participant will visit a local participating laboratory (through the Quest national chain of commercial laboratories) to confirm that their LDL is greater than or equal to 100 (if a diagnosis of CVD or diabetes) or their LDL is greater than 190 for non-diabetic or CVD individuals. Enrollment will include a description of the voluntary nature of participation, the study procedures, risks and potential benefits in detail. The enrollment procedure will provide the opportunity for potential participants to ask questions and review the consent form information with family and friends prior to making a decision to participate. Participants will be told that they do not have to answer any questions if they do not wish and can drop out of the study at any time, without affecting their medical care or the cost of their care. They will be told that they may not benefit directly from the study and that all information will be kept strictly confidential, except as required by law. Subjects will be given a copy of the consent document. All efforts will be made by study staff to ensure subject privacy.

Data Disclosure

Will the data be disclosed to anyone who is not listed under Personnel?

The following entities, besides the members of the research team, may receive PHI for this research study: Vitality, Inc., Medsignals, [redacted], or Wisepill the companies which records the responses from the electronic pill container. Daily adherence information will be stored on their secure computers. Quest Diagnostics, the company that will be used for blood sample collection and analysis. Labs will be ordered on Quest's secure server know as Care 360 and lab results from Quest will be made available via Care 360, secure Quest server. The Office of Human Research Protections at the University of Pennsylvania Federal and state agencies (for example, the Department of Health and Human Services, the National Institutes of Health, and /or the Office for Human Research Protections), or other domestic or foreign government bodies if required by law and/or necessary for oversight purposes A data and safety monitoring board organized to oversee this research

*Data Protection**

x **Name**
x **Street address, city, county, precinct, zip code, and equivalent geocodes**
x **All elements of dates (except year) for dates directly related to an individual and all ages over 89**
x **Telephone and fax number**
x **Electronic mail addresses**
x **Social security numbers**
x **Medical record numbers**
x **Health plan ID numbers**
Account numbers
Certificate/license numbers
Vehicle identifiers and serial numbers, including license plate numbers
Device identifiers/serial numbers
Web addresses (URLs)
Internet IP addresses
Biometric identifiers, incl. finger and voice prints
Full face photographic images and any comparable images
Any other unique identifying number, characteristic, or code
None

Does your research request both a waiver of HIPAA authorization for collection of patient information and involve providing Protected Health Information ("PHI") that is classified as a "limited data set" (city/town/state/zip code, dates except year, ages less than 90 or aggregate report for over 90) to a recipient outside of the University of Pennsylvania covered entity?
No

Tissue Specimens Obtained as Part of Research*
Are Tissue Specimens being obtained for research?
No

Tissue Specimens - Collected during regular care*
Will tissue specimens be collected during regulator clinical care (for treatment or diagnosis)?
No

Tissue Specimens - otherwise discarded*
Would specimens otherwise be discarded?
No

Tissue Specimens - publicly available*
Will tissue specimens be publicly available?
No

Tissue Specimens - Collected as part of research protocol*
Will tissue specimens be collected as part of the research protocol?
No

Tissue Specimens - Banking of blood, tissue etc. for future use*
Does research involve banking of blood, tissue, etc. for future use?
No

Genetic testing
If genetic testing is involved, describe the nature of the tests, including if the testing is predicative or exploratory in nature. If predictive, please describe plan for disclosing results to subjects and provision

of genetic counseling. Describe how subject confidentiality will be protected Note: If no genetic testing is to be obtained, write: "Not applicable."
Not applicable

Consent

1. Consent Process

Overview
Potential participants will be identified by reviewing CVS Caremark pharmacy claims records using our eligibility criteria of statin use and MPR less than or equal to 80%. CVS Caremark will facilitate communication to these members using existing communication channels (email, mail). Potentially eligible individuals will be invited to visit the WTH portal or to call our study staff to enroll. Potentially eligible individuals can enroll directly through the WTH platform, which steps them through screening and consent process electronically or may have study staff walk them through the consent process on the phone. Preparatory to Research For the recruitment phase, CVS Caremark must review PHI consisting of pharmacy claims data to identify qualified potential study subjects, and then must further use PHI to communicate with such potential subjects to provide them with recruitment materials. CVS Caremark holds the pharmacy claims data in its role as a PBM and business associate to the employer-sponsored health plans. As a result, CVS Caremark must obtain the consent of the health plans prior to using the pharmacy claims records for these recruitment purposes. Further, CVS Caremark must be authorized by its business associate agreement with the health plan to conduct the preparatory to research activities. As required by HIPAA to conduct preparatory to research activities, CVS Caremark, as a researcher and business associate of the health plans, must provide representations to the employer-sponsored health plans that (i) the use or disclosure is sought solely to review PHI as necessary for purposes preparatory to research, i.e., identifying potential study participants; (ii) CVS Caremark will not remove any PHI from the health plans in the course of the review, i.e., CVS Caremark will only access PHI maintained on behalf of the health plans; and (iii) the PHI for which use or access is sought is necessary for the research purposes. The receipt of such assurances will satisfy the health plans' obligation to only permit uses and disclosures of the PHI of their members as authorized under HIPAA. CVS Caremark will take the following steps so that its access to and use of the PHI will involve no more than a minimal risk to the privacy of health plan members: First of all, steps that will be taken to protect the PHI while it is being accessed and analyzed to identify eligible study participants. CVS Caremark will only permit a very limited number of its personnel to access the PHI to conduct the analysis. All such personnel will be trained on HIPAA-compliance, including that they cannot further use or disclose the PHI for any purposes except for the specific study recruitment activities. CVS Caremark will apply its standard security policies, procedures and measures to protect the security of the data. The PHI will be encrypted and stored on CVS Caremarks password protected SASNODE server. Steps will also be taken to protect the PHI when CVS Caremark communicates to the potential study participants about enrolling in the study. Such steps include making sure that any written materials are sent in a manner that does not unnecessarily disclose the individual's diagnosis or status. If written materials are being sent in the mail, they will be enclosed in an envelope and e-mails will only be sent if it would otherwise be appropriate to send PHI to that individual via e-mail. Personnel, including vendors, who will be making the communications will also be trained on HIPAA-compliance, including that they cannot further use or disclose the PHI for any purposes except for the specific study recruitment activities. CVS Caremark personnel who will be conducting the recruitment activities will destroy the identifiers at the earliest opportunity consistent with conduct of the research, unless there is a health or research justification for retaining the identifiers or such retention is otherwise required by law. As a Business Associate, CVS Caremark is prohibited by both HIPAA and by its business associate agreements with the health plans to not reuse or disclose the PHI to any other person or entity, except as required by law or as otherwise permitted under HIPAA. In addition, the research agreement between CVS Caremark and UPenn also prohibits CVS Caremark from using or disclose the PHI except as required by law or other permitted under HIPAA. Upon identification of potentially eligible participants, CVS Caremark, as a business associate on behalf of the health plans, will communicate recruitment materials created by Penn and will do so using existing communication channels it has with such individuals, consistent with the measures identified above to protect the privacy of the individuals. Potentially eligible individuals will be invited to visit the WTH portal or to call our study staff to enroll.

Individuals interested in enrolling who agree to provide written consent (See Protection of Human Subjects) will complete an intake form and consent using our Way to Health web portal. CVS Caremark will not disclose or otherwise provide any PHI until it receives evidence of executed written consent forms containing valid HIPAA-compliant language authorizing CVS Caremark to do so. Penn Medicine patients will receive a follow-up phone call from a study coordinator one week after the recruitment letter is sent to them. At this time, the coordinator will describe the study to the participant and if interested, will offer to begin enrollment over the phone. The participant will verbally complete the screening survey with the coordinator recording their answers in Way to Health. The coordinator will then go over the consent form with the participant and ask if they agree to take part in the study. If they agree to participate the participant will be mailed a copy of the consent form. The participant will then be asked to log on to the platform to complete the baseline survey before being fully enrolled into the study. This ensures that the participant has internet access and will be able to engage with the platform throughout the study.

Children and Adolescents
There will be no children or adolescents enrolled in this study.

Adult Subjects Not Competent to Give Consent
Adult subjects will be competent to give informed consent.

2. Waiver of Consent

Waiver or Alteration of Informed Consent*
Waiver of written documentation of informed consent: the research presents no more than minimal risk of harm to subjects and involves no procedures for which written consent is normally required outside of the research context

Minimal Risk*

Impact on Subject Rights and Welfare*

Waiver Essential to Research*

Additional Information to Subjects

Written Statement of Research*
No

If no written statement will be provided, please provide justificiation

This specific modification does not involve the subjects enrolled in the study. See all required documents requested by the IRB from our initial submission and response from the IRB.

The following documents are currently attached to this item:

There are no documents attached for this item.

Risk / Benefit

Potential Study Risks
Risks Involved in the Main Study: There are minimal risks associated with providing sweepstake-based financial incentives to improve statin adherence. The main risk is loss of confidentiality, which will be protected as described below. Another small risk exists for local trauma (hematoma) during blood draw for cholesterol level /lipid testing, as well as bruising, bleeding, infection and fainting. However, this is a minimal risk since blood will be drawn by skilled phlebotomists through Quest Diagnostics. There are no potential risks associated with any other measures or data to be collected. During the consent process, we will inform subjects of the risks associated with blood draws and loss of confidentiality. Database Security/Protection against Risk. To assure that patient, physician and other informant confidentiality is preserved, individual identifiers (such as name and medical record number/physician billing identifier) are stored in a single password protected system that is accessible only to study

research, analysis and IT staff. This system is hosted on site at The University of Pennsylvania (UPenn) and is protected by a secure firewall. Once a participant is in this system, they will be given a unique study identification number (ID). Any datasets and computer files that leave the firewall will be stripped of all identifiers and individuals will be referred to by their study ID. The study ID will also be used on all analytical files. Additionally, any information that leaves this system to communicate with third party data sources (biometrics devices, survey software, etc.) will be stripped of any identifiers and transmitted in encrypted format. The same unique study ID will be used to link these outside data to the participants. Social security numbers, bank account and routing numbers for all participants to whom payments are sent will also be transmitted in encrypted format to UPenns Financial Systems/ Comptrollers Department where data will be stored for compliance with W-9 form reporting requirements. After the social security numbers are no longer needed they will be deleted from our system. The Way to Health (WTH) Research Data Center staff is responsible for preventing unauthorized access to the trial participant tracking system database. It is important to note that the Way to Health database server and individual study databases have never been compromised as a result of the extremely rigorous and secure network firewall technologies. The secure servers are located in a specially designed, highly secured facility at UPenn with dedicated uninterrupted power supply and strictly limited access. The study will utilize a client-server deployed Data Management System (DMS) rather than a 'Store and Forward' database configuration, obviating research site database security concerns. Confidential participant information will be entered into the database. If this information exists on paper CRFs, it will be filed under lock and key, with generation of a participant ID. Thereafter, confidential information will be made available to authorized users only as specifically needed. No one can gain access to an individual MySQL database table unless explicitly granted a user ID, password, and specific access. Even those with user names and passwords cannot gain access to the tables that contain the identifying participant information. No results will be reported in a personally identifiable manner. All tracking system data will be password-protected with several levels of protection. The first will allow access to the operating system of the computer. The second will allow access to the basic menus of the integrated system; within certain menu options, such as database browsing, a third password will be required. Our prior research employing similar precautions has demonstrated that these techniques are very successful in assuring the protection of subjects. The same procedure used for the analysis of automated data sources to ensure protection of patient information will be used for the survey data, in that patient identifiers will be used only for linkage purposes or to contact patients. The study identification number, and not other identifying information, will be used on all data collection instruments. All study staff will be reminded to appreciate the confidential nature of the data collected and contained in these databases.

Potential Study Benefits

Participants in this study may not receive any direct benefits. Some may benefit directly by improving their adherence to statin medications and thus lowering their risk for future heart attacks, strokes and death, improved quality of life, and reduced medical care costs. The control group is unlikely to directly benefit, but this group will continue to receive usual care. Knowledge gained from the study will assist in development of interventions in others who are not adherent to their prescribed medications. The potential public health impact of a successful intervention to improve adherence to statin medications is enormous and could reduce the number of deaths from heart attacks and strokes by tens of thousands in the United States each year. The risks of loss of confidentiality are minimal in this study. Thus, the benefits of this research to the participants studied, and to society at large, far surpass the risks.

Alternatives to Participation (optional)

To not participate in the study.

Data and Safety Monitoring

The data and safety monitoring plan will have three parts. First, the BMIC will develop and implement methods of verifying entered data and of quality control. Second, the PI will be directly responsible for identifying and reporting all serious adverse events, protocol deviations/violations and unanticipated events to the IRBs and funding agency promptly, as appropriate. The PI will also report all adverse events, accrual rates, retention rates, and all other logistical issues to the DSMB (described below) at least biannually (and more frequently if there are serious adverse events). Third, there will be a DSMB responsible for monitoring the trial. A written research protocol will undergo formal institutional scientific and institutional review board (IRB) review at the University of Pennsylvania (UPENN) to ensure protection of the rights and welfare of human research subjects. Specifically, the multiple principal investigators (PIs) and the IRB will be responsible for ensuring risks to human subjects are

minimized, risks are reasonable, subject selection is equitable, the research team has access to adequate resources to conduct the study, the informed consent process meets regulatory and ethical requirements, adequate provision is made to protect human subjects by monitoring the data collected and there are adequate provisions to protect subject privacy per HIPAA regulations and confidentiality of data. All senior/key personnel and research staff who will be involved in the design and conduct of the study must receive education in human research subjects protection from a training program that is approved by a properly constituted independent Ethics Committee or Institutional Review Board. The multiple PIs will be responsible for ensuring project faculty and staff have the equipment and training required to protect privacy and confidentiality and will monitor and document that these individuals are properly certified. If new senior/key personnel and staff become involved in the research, documentation that they have received the required education will be included in the annual progress reports. The UPENN Office of Regulatory Affairs currently requires HIPAA training upon designation as research investigator/staff and recertification in human research subjects protection every three years. The UPENN IRB will serve as the IRB of record for any external ethics review boards or IRBs applicable to researchers from other institutions who may have access to human research subjects identified data. Data and Safety Monitoring Board. The DSMB will be composed of experts in clinical trials, medical economics, general internal medicine, and biostatistics, along with multiple PIs Drs. Barankay and Volpp as non-voting members. We consider the proposed trial to be relatively low risk. Therefore, we have arranged for a monitoring committee that is assigned to review the study and staff training protocols, monitor the trial for safety and adverse events, and conduct a semi-annual meeting. These members will not be involved directly with the trial. The members that we propose to serve on this committee and their activities are: Philip Greenland, M.D.: Dr. Greenland is the Harry W. Dingham Professor and Senior Associate Dean for Clinical and Translational Research at Northwestern University and Director of the Northwestern University Clinical and Translational Sciences (NUCATS) Institute. He is a well-known expert in the field of prevention of cardiovascular disease. Michael K. Parides, Ph.D.: Dr. Parides is Professor of Health Policy at the Mount Sinai School of Medicine, and Director of Biostatistics at the International Center for Health Outcomes and Innovation Research (InCHOIR) who specializes in clinical trials, linear models and analysis of categorical data. He has extensive experience in both conducting and monitoring randomized trials of health interventions with a particular focus in cardiology. Allison Rosen, M.D., M.P.H, Sc.D.: Dr. Rosen is an Associate Professor of Quantitative Health Sciences at the University of Massachusetts Medical School. Her research includes finding ways to increase the use of underutilized therapies in patients with chronic diseases. Risk of loss of confidentiality will be minimized by storing completed paper copies of the surveys and signed informed consent forms in locked file cabinets in locked offices accessible only to trained study staff. Each subject will be assigned a unique identifier without identifying information, and data will be entered into an electronic database using only the unique identifier. Only trained study staff will have access to the code that links the unique identifier to the subjects identity. Electronic data will be stored on secure, password-protected firewalled servers at UPENN. The DSMB members will perform several duties. First, they will review and approve the research protocol and plans for data and safety monitoring prior to initiation of the study. Second, they will evaluate the progress of the trial. This will include assessment of data quality, participant recruitment, accrual and retention, participant risk versus benefit, performance of trial sites, and study outcomes. This assessment will be performed at meetings every 6 months during the clinical trials and more frequently if needed. Third, they will make recommendations to ensure that all of the issues above are appropriately addressed. The multiple PIs of the project will be responsible for responding to all recommendations of the DSMB and submitting DSMB reports to the respective IRBs.

The following documents are currently attached to this item:

There are no documents attached for this item.

Risk / Benefit Assessment

Poor statin medication adherence is a major public health problem with few scalable, cost-effective solutions. This study is designed to test two novel interventions that, if successful, will provide the research and public health communities with critically important information about these new methods. This approach has potentially broad generalizability in treating people at risk for coronary events and death nationally, as these types of sweepstakes could be set up by insurers and broadly utilized. Moreover, the interventions can serve as a model for improving adherence among other medications for chronic diseases. Because of the large scientific and public health benefits of the knowledge gained from this study, the minimal risks to participants are reasonable in relation to the importance of the

knowledge that reasonably may be expected to result.

General Attachments

The following documents are currently attached to this item:

Cover Letter (irbcoverletter_3.20.2019.doc)

Additional forms (cititrainingrefreshercourse_yoonheeha.pdf)
