# Supplementary material for: Effect of Patient Financial Incentives on Statin Adherence and Lipid Control: A Randomized Clinical Trial
Source: JAMA Netw Open. 2020 Oct 9;3(10):e2019429. doi: 10.1001/jamanetworkopen.2020.19429 (PMC7547367; doi:10.1001/jamanetworkopen.2020.19429)
Supplement: Supplement 2. — eTable 1. Baseline Characteristics by Arm eTable 2. Measured Adherence 0-6 Months (Means, 95% CI) by Arm eTable 3. Measured Adherence During Final 30 Days of Intervention (Means, 95% CI) by Arm eTable 4. Change in LDL-C From Baseline to 12 Months (Means, 95% CI) by Arm eTable 5. Penn Medicine: Baseline Characteristics of Participants With and Without a Post-Study LDL-C Measurement eFigure 1. Penn Medicine: Change in LDL-C Post-Study With Loess Smoothed Curves eTable 6. Penn Medicine: Baseline Characteristics of Eligible Nonenrolled Patients Compared to Enrolled Participants in Control Group eFigure 2. Penn Medicine: Nonenrolled Individuals: Change in LDL-C From Baseline With Loess Smoothing Curves eTable 7. Penn Medicine: Mean (95% CI) Change From Baseline in LDL-C at 6 and 12 Months for Participants Enrolled to the Control Arm and for Nonenrolled Individuals eTable 8. Regression Models for the Reduction in ΔLDL From Baseline to Either 6- or 12-Months by 6-Month Measured Adherence (Proportion of Days With Electronic Pill Bottle Opened Over 180 Days) eFigure 3. Change in LDL-C From Baseline to 6 Months in Individuals With Measured Adherence of Zero During Either the Entire 6 Months (n=2) or the Final 30 Days of the Intervention (n=23 for Control and n=16 for Incentives) [file jamanetwopen-e2019429-s002.pdf]

Supplemental Online Content

Barankay I, Reese PP, Putt ME, et al. Effect of patient financial incentives on statin adherence and lipid control: a randomized clinical trial. *JAMA Netw Open*. 2020;3(10):e2019429. doi:10.1001/jamanetworkopen.2020.19429

eTable 1. Baseline Characteristics by Arm

eTable 2. Measured Adherence 0-6 Months (Means, 95% CI) by Arm

eTable 3. Measured Adherence During Final 30 Days of Intervention (Means, 95% CI) by Arm

eTable 4. Change in LDL-C From Baseline to 12 Months (Means, 95% CI) by Arm

eTable 5. Penn Medicine: Baseline Characteristics of Participants With and Without a Post-Study LDL-C Measurement

eFigure 1. Penn Medicine: Change in LDL-C Post-Study With Loess Smoothed Curves

eTable 6. Penn Medicine: Baseline Characteristics of Eligible Nonenrolled Patients Compared to Enrolled Participants in Control Group

eFigure 2. Penn Medicine: Nonenrolled Individuals: Change in LDL-C From Baseline With Loess Smoothing Curves

eTable 7. Penn Medicine: Mean (95% CI) Change From Baseline in LDL-C at 6 and 12 Months for Participants Enrolled to the Control Arm and for Nonenrolled Individuals

eTable 8. Regression Models for the Reduction in Δ_{LDL} From Baseline to Either 6- or 12-Months by 6-Month Measured Adherence (Proportion of Days With Electronic Pill Bottle Opened Over 180 Days)

eFigure 3. Change in LDL-C From Baseline to 6 Months in Individuals With Measured Adherence of Zero During Either the Entire 6 Months (n=2) or the Final 30 Days of the Intervention (n=23 for Control and n=16 for Incentives)

This supplemental material has been provided by the authors to give readers additional information about their work.

© 2020 Barankay I et al. *JAMA Network Open.*

1 Descriptive Analyses of Baseline Participant Characteristics

Characteristics collected at baseline but not included in Table 1 of main paper. This analysis is based on the original statistical analysis plan.

1.1 eTable 1. Baseline Characteristics by Arm

Characteristic	Total (n=805)	Control (n=201)	Sweepstakes (n=199)	Deadline Sweepstakes (n=204)	Sweepstakes & Contract (n=201)
Health Condition, n (%)					
Excellent	29 (3.6)	4 (2.0)	8 (4.0)	6 (2.9)	11 (5.5)
Very Good	181 (22.5)	53 (26.4)	37 (18.6)	42 (20.6)	49 (24.4)
Good	365 (45.3)	86 (42.8)	93 (46.7)	98 (48.0)	88 (43.8)
Fair	211 (26.2)	56 (27.9)	57 (28.6)	54 (26.5)	44 (21.9)
Poor	19 (2.4)	2 (1.0)	4 (2.0)	4 (2.0)	9 (4.5)
Stages of Change, n (%)[a]					
Maintenance Stage	549 (68.2)	140 (69.7)	124 (62.3)	144 (70.6)	141 (70.1)
Action Stage	220 (27.3)	54 (26.9)	63 (31.7)	53 (26.0)	50 (24.9)
Preparation Stage	26 (3.2)	4 (2.0)	10 (5.0)	5 (2.5)	7 (3.5)
Contemplation Stage	8 (1.0)	2 (1.0)	1 (0.5)	2 (1.0)	3 (1.5)
Pre-contemplation Stage	2 (0.2)	1 (0.5)	1 (0.5)	0 (0.0)	0 (0.0)
Patient Activation Measure Level, n (%)[b]					
May not yet believe that the patient role is important	23 (2.9)	7 (3.5)	5 (2.5)	5 (2.5)	6 (3.0)
Lacks confidence and knowledge to take action	29 (3.6)	7 (3.5)	8 (4.0)	8 (3.9)	6 (3.0)
Beginning to take action	85 (10.6)	21 (10.4)	20 (10.1)	23 (11.3)	21 (10.4)
Has difficulty maintaining behaviors over time	668 (83.0)	166 (82.6)	166 (83.4)	168 (82.4)	168 (83.6)
Financial incentives will help me to take my statin[c], n (%)					
Strongly disagree	127 (15.8)	30 (14.9)	39 (19.6)	27 (13.3)	31 (15.4)
Somewhat disagree	112 (13.9)	26 (12.9)	28 (14.1)	30 (14.8)	28 (13.9)
Neither agree or disagree	234 (29.1)	59 (29.4)	54 (27.1)	65 (32.0)	56 (27.9)
Agree	212 (26.4)	55 (27.4)	45 (22.6)	51 (25.1)	61 (30.3)
Strongly agree	119 (14.8)	31 (15.4)	33 (16.6)	30 (14.8)	25 (12.4)
Financial incentives will help ot hers to take their statin[c], n (%)					
Strongly disagree	36 (4.5)	10 (5.0)	9 (4.5)	6 (3.0)	11 (5.5)
Somewhat disagree	57 (7.1)	18 (9.0)	13 (6.5)	13 (6.4)	13 (6.5)
Neither agree or disagree	236 (29.4)	56 (27.9)	59 (29.6)	55 (27.1)	66 (32.8)
Agree	346 (43.0)	85 (42.3)	82 (41.2)	101 (49.8)	78 (38.8)
Strongly agree	129 (16.0)	32 (15.9)	36 (18.1)	28 (13.8)	33 (16.4)
Household Size[c], mean (SD)	2.5 (1.4)	2.6 (1.4)	2.7 (1.5)	2.4 (1.5)	2.5 (1.3)

© 2020 Barankay I et al. *JAMA Network Open.*

Characteristic	Total (n=805)	Control (n=201)	Sweepstakes (n=199)	Deadline Sweepstakes (n=204)	Sweepstakes & Contract (n=201)
Financial situation[c,g], n (%)					
Very constrained	150 (18.7)	38 (18.9)	42 (21.2)	35 (17.2)	35 (17.5)
Somewhat constrained	228 (28.4)	65 (32.3)	57 (28.8)	55 (27.1)	51 (25.5)
Neutral	205 (25.6)	44 (21.9)	52 (26.3)	49 (24.1)	60 (30.0)
Somewhat comfortable	171 (21.3)	43 (21.4)	34 (17.2)	55 (27.1)	39 (19.5)
Very comfortable	48 (6.0)	11 (5.5)	13 (6.6)	9 (4.4)	15 (7.5)
Spare money this week[c,d], mean (SD)	3.7 (2.8)	3.7 (2.7)	3.4 (2.5)	3.9 (2.9)	3.9 (3.0)
Able to pay next month[c,e,g] mean (SD)	4.2 (3.8)	3.9 (3.7)	4 (3.7)	4.6 (3.9)	4.3 (3.9)
Are you generally fully prepared to take risks[c,f,g], mean (SD)	4.9 (2.6)	5 (2.6)	4.7 (2.5)	5.1 (2.7)	5 (2.8)

[a]Nigg, C.R., Burbank, P., Padula, C., Dufresne, R., Rossi, J. S., Velicer, W. F., Laforge, R. G. & Prochaska, J. O. (1999). Stages of change across ten health risk behaviors for older adults. *The Gerontologist*, 39, 473-482

[b] Insignia Health. Patient Activation Measure; Copyright 2003-2010, University of Oregon. All Rights reserved."

[c] Not all participants responded; incomplete data.

[d] Think about your specific expenses and your available spare money this week. How much available spare money do you have this week? This question scores from 1 – Very little available money to 11 – A lot of available money

[e] Imagine that next month you had an unexpected expense of $1,500 such as a medical bill or a necessary car repair. How likely is it that you would be able to pay this bill in full and on time without having to dip into your retirement fund, borrow money or charge it to a credit card? This question scores from 1 – Very Unlikely to 11 – Very likely

[f] Score from 1 – Not prepared to take risks to 10 – Fully prepared to take risks

[g] From SLACK questionnaire as described in: Zauberman, G. & Lynch, J.G. (2005) Resource Slack and Propensity to Discount Delayed Investments of Time Versus Money. *Journal of Experimental Psychology: General*, 134, 23-37.

2 Measured Adherence Analyses

2.1 eTable 2. Measured Adherence 0-6 Months (Means, 95% CI) by Arm

This analysis is based on the original statistical analysis plan.

Subgroup	Analysis	Metric	Arm			
			**Control**	**Simple Sweepstakes**	**Deadline Sweepstakes**	**Sweepstakes & Deposit**
Gender	Female (n=519)	Individual Group	0.64 (0.60, 0.68)	0.82 (0.78, 0.87)	0.84 (0.80, 0.88)	0.87 (0.83, 0.91)
		Difference from control		0.18 (0.12, 0.24)	0.20 (0.14, 0.26)	0.22 (0.17, 0.28)
		P-value		<.001	<.001	<.001
	Male (n=286)	Individual Group	0.77 (0.73, 0.80)	0.87 (0.83, 0.90)	0.88 (0.84, 0.92)	0.89 (0.85, 0.93)
		Difference from control		0.10 (0.05, 0.15)	0.12 (0.06, 0.17)	0.12 (0.07, 0.18)
		P-value		<.001	<.001	<.001
Race	Black (n=384)	Individual Group	0.65 (0.60, 0.69)	0.81 (0.77, 0.86)	0.83 (0.78, 0.87)	0.85 (0.80, 0.89)
		Difference from control		0.16 (0.10, 0.23)	0.18 (0.12, 0.25)	0.20 (0.14, 0.27)
		P-value		<.001	<.001	<.001
	White (n=370)	Individual Group	0.75 (0.71, 0.78)	0.87 (0.83, 0.91)	0.89 (0.85, 0.93)	0.90 (0.86, 0.94)
		Difference from control		0.13 (0.07, 0.18)	0.14 (0.09, 0.19)	0.15 (0.10, 0.21)
		P-value		<.001	<.001	<.001
	Other (n=53)	Individual Group	0.53 (0.40, 0.66)	0.83 (0.69, 0.97)	0.88 (0.74, 1.02)	0.86 (0.73, 0.99)
		Difference from control		0.30 (0.10, 0.49)	0.35 (0.16, 0.54)	0.33 (0.14, 0.52)
		P-value		0.003	<.001	<.001
Income	Below $50,000 (n=414)	Individual Group	0.67 (0.62, 0.71)	0.83 (0.78, 0.87)	0.84 (0.79, 0.88)	0.87 (0.82, 0.91)
		Difference from control		0.16 (0.10, 0.23)	0.17 (0.11, 0.23)	0.20 (0.14, 0.26)
		P-value		<.001	<.001	<.001
	Above $50,000 (n=380)	Individual Group	0.72 (0.68, 0.75)	0.85 (0.81, 0.89)	0.88 (0.84, 0.91)	0.88 (0.85, 0.92)
		Difference from control		0.13 (0.08, 0.19)	0.16 (0.11, 0.21)	0.17 (0.11, 0.22)
		P-value		<.001	<.001	<.001
Baseline LDL levels	100-129 mg/l (n=419)	Individual Group	0.71 (0.67, 0.75)	0.84 (0.79, 0.88)	0.87 (0.83, 0.91)	0.88 (0.84, 0.92)
		Difference from control		0.13 (0.07, 0.19)	0.16 (0.10, 0.21)	0.17 (0.12, 0.23)
		P-value		<.001	<.001	<.001

© 2020 Barankay I et al. *JAMA Network Open.*

Subgroup	Analysis	Metric	Arm			
			Control	Simple Sweepstakes	Deadline Sweepstakes	Sweepstakes & Deposit
Baseline LDL levels	130-159 mg/l (n=173)	Individual Group	0.68 (0.62, 0.75)	0.84 (0.78, 0.90)	0.87 (0.80, 0.93)	0.86 (0.80, 0.92)
		Difference from control		0.16 (0.07, 0.25)	0.18 (0.09, 0.27)	0.17 (0.09, 0.26)
		P-value		<.001	<.001	<.001
	160-189 mg/l (n=70)	Individual Group	0.68 (0.60, 0.77)	0.87 (0.79, 0.95)	0.85 (0.77, 0.94)	0.88 (0.76, 1.00)
		Difference from control		0.18 (0.06, 0.30)	0.17 (0.05, 0.29)	0.20 (0.05, 0.34)
		P-value		0.003	0.008	0.011
	>190 mg/l (n=143)	Individual Group	0.63 (0.56, 0.70)	0.82 (0.75, 0.90)	0.81 (0.73, 0.90)	0.86 (0.79, 0.94)
		Difference from control		0.19 (0.09, 0.30)	0.18 (0.07, 0.29)	0.23 (0.13, 0.34)
		P-value		<.001	0.001	<.001

2.2 eTable 3. Measured Adherence During Final 30 Days of Intervention (Means, 95% CI) by Arm

This analysis is based on post-hoc additions to the statistical analysis plan.

Subgroup	Analysis	Metric	Arm			
			Control	Simple Sweepstakes	Deadline Sweepstakes	Sweepstakes & Deposit
Gender	Female (n=519)	Individual Group	0.54 (0.49, 0.60)	0.74 (0.69, 0.80)	0.81 (0.75, 0.86)	0.82 (0.77, 0.88)
		Difference from control		0.20 (0.12, 0.28)	0.27 (0.19, 0.34)	0.28 (0.20, 0.36)
		P-value		<.001	<.001	<.001
	Male (n=286)	Individual Group	0.68 (0.62, 0.73)	0.82 (0.76, 0.87)	0.86 (0.81, 0.92)	0.87 (0.81, 0.92)
		Difference from control		0.14 (0.06, 0.22)	0.19 (0.11, 0.27)	0.19 (0.11, 0.27)
		P-value		<.001	<.001	<.001
Race	Black (n=384)	Individual Group	0.55 (0.48, 0.61)	0.74 (0.68, 0.80)	0.79 (0.73, 0.85)	0.79 (0.73, 0.86)
		Difference from control		0.19 (0.11, 0.28)	0.24 (0.15, 0.33)	0.25 (0.15, 0.34)
		P-value		<.001	<.001	<.001
	White (n=370)	Individual Group	0.65 (0.60, 0.71)	0.80 (0.74, 0.86)	0.87 (0.81, 0.93)	0.88 (0.83, 0.94)
		Difference from control		0.15 (0.07, 0.23)	0.22 (0.14, 0.30)	0.23 (0.15, 0.31)
		P-value		<.001	<.001	<.001

© 2020 Barankay I et al. *JAMA Network Open.*

Subgroup	Analysis	Metric	Arm			
			**Control**	**Simple Sweepstakes**	**Deadline Sweepstakes**	**Sweepstakes & Deposit**
Race	Other (n=53)	Individual Group	0.45 (0.28, 0.62)	0.80 (0.61, 0.99)	0.86 (0.67, 1.04)	0.85 (0.67, 1.02)
		Difference from control		0.35 (0.09, 0.61)	0.41 (0.15, 0.66)	0.40 (0.15, 0.64)
		P-value		0.008	0.003	0.002
Income	Below $50,000 (n=414)	Individual Group	0.56 (0.49, 0.62)	0.77 (0.71, 0.83)	0.81 (0.75, 0.87)	0.82 (0.75, 0.88)
		Difference from control		0.21 (0.13, 0.30)	0.25 (0.17, 0.34)	0.26 (0.17, 0.35)
		P-value		<.001	<.001	<.001
	Above $50,000 (n=380)	Individual Group	0.64 (0.58, 0.69)	0.77 (0.72, 0.83)	0.85 (0.79, 0.90)	0.87 (0.82, 0.92)
		Difference from control		0.14 (0.06, 0.21)	0.21 (0.13, 0.29)	0.23 (0.16, 0.31)
		P-value		<.001	<.001	<.001
Baseline LDL levels	100-129 mg/l (n=419)	Individual Group	0.62 (0.56, 0.68)	0.76 (0.70, 0.82)	0.84 (0.79, 0.90)	0.86 (0.81, 0.92)
		Difference from control		0.14 (0.05, 0.22)	0.22 (0.14, 0.30)	0.24 (0.16, 0.32)
		P-value		0.001	<.001	<.001
	130-159 mg/l (n=173)	Individual Group	0.55 (0.46, 0.65)	0.77 (0.68, 0.86)	0.82 (0.72, 0.92)	0.82 (0.73, 0.90)
		Difference from control		0.22 (0.08, 0.35)	0.26 (0.13, 0.40)	0.26 (0.13, 0.39)
		P-value		0.001	<.001	<.001
	160-189 mg/l (n=70)	Individual Group	0.56 (0.42, 0.70)	0.80 (0.67, 0.93)	0.80 (0.67, 0.94)	0.80 (0.61, 0.99)
		Difference from control		0.24 (0.05, 0.43)	0.25 (0.05, 0.44)	0.24 (0.01, 0.48)
		P-value		0.014	0.014	0.044
	>190 mg/l (n=143)	Individual Group	0.56 (0.47, 0.66)	0.78 (0.67, 0.88)	0.80 (0.69, 0.91)	0.81 (0.70, 0.91)
		Difference from control		0.21 (0.07, 0.35)	0.24 (0.09, 0.38)	0.24 (0.10, 0.38)
		P-value		0.003	0.001	<.001

© 2020 Barankay I et al. *JAMA Network Open.*

3 Change in LDL-c Analyses

3.1 Change in LDL-c during the study

3.1.1 eTable 4. Change in LDL-C From Baseline to 12 Months (Means, 95% CI) by Arm [a]

This analysis is based on the original statistical analysis plan.

Subgroup	Analysis	Metric	Arm			
			Control	Simple Sweepstakes	Deadline Sweepstakes	Sweepstakes & Deposit
-	Complete Case, adjusting for baseline LDL[b] (n=636)	Individual Group	-33.9 (-39.2, -28.6)	-32.9 (-38.2, -27.7)	-33.2 (-38.4, -28.1)	-36.9 (-42.2, -31.7)
		Difference from control		0.9 (-6.5, 8.4)	0.6 (-6.8, 8.0)	-3.0 (-10.5, 4.4)
		P-value		0.803	0.866	0.422
-	Without adjusting for baseline LDL (n=805)	Individual Group	-35.8 (-42.1, -29.4)	-33.9 (-40.2, -27.6)	-31.5 (-37.8, -25.3)	-34.6 (-40.9, -28.2)
		Difference from control		1.8 (-7.1, 10.7)	4.2 (-4.7, 13.2)	1.2 (-7.7, 10.1)
		P-value		0.687	0.354	0.790
-	Adjusting for baseline LDL, race, gender, income and education (n=805)	Individual Group	-39.3 (-49.1, -29.4)	-38.6 (-48.3, -28.9)	-39.1 (-48.4, -29.7)	-42.1 (-51.7, -32.5)
		Difference from control		-0.6 (-7.8, 6.5)	-0.4 (-7.7, 6.8)	-3.5 (-10.8, 3.8)
		P-value		0.862	0.910	0.350
Gender	Female, adjusting for baseline LDL (n=519)	Individual Group	-31.6 (-38.1, -25.2)	-30.9 (-37.4, -24.4)	-31.0 (-37.3, -24.6)	-34.5 (-41.1, -27.8)
		Difference from control		0.7 (-8.4, 9.9)	0.6 (-8.6, 9.8)	-2.9 (-12.0, 6.3)
		P-value		0.874	0.893	0.542
	Male, adjusting for baseline LDL (n=286)	Individual Group	-37.3 (-45.7, -28.8)	-35.5 (-43.6, -27.4)	-37.1 (-45.3, -28.9)	-40.0 (-48.2, -31.7)
		Difference from control		1.8 (-9.9, 13.5)	0.2 (-11.5, 11.9)	-2.7 (-14.3, 8.9)
		P-value		0.762	0.975	0.650
Race	Black, adjusting for baseline LDL (n=384)	Individual Group	-29.9 (-37.8, -22.1)	-30.9 (-38.1, -23.7)	-24.9 (-32.1, -17.6)	-31.9 (-39.8, -24.0)
		Difference from control		-1.0 (-11.7, 9.7)	5.1 (-5.6, 15.8)	-2.0 (-13.0, 9.1)
		P-value		0.859	0.352	0.728
	White, adjusting for baseline LDL (n=370)	Individual Group	-36.0 (-42.9, -29.0)	-32.4 (-39.9, -25.0)	-39.6 (-46.8, -32.4)	-39.6 (-46.6, -32.6)
		Difference from control		3.6 (-6.5, 13.6)	-3.6 (-13.5, 6.3)	-3.6 (-13.3, 6.1)
		P-value		0.489	0.472	0.468
	Other, adjusting for baseline LDL (n=53)	Individual Group	-39.7 (-61.1, -18.4)	-42.2 (-63.8, -20.6)	-58.1 (-81.0, -35.2)	-42.8 (-63.9, -21.7)
		Difference from control		-2.5 (-32.7, 27.7)	-18.3 (-49.3, 12.6)	-3.0 (-32.7, 26.6)
		P-value		0.872	0.246	0.841

© 2020 Barankay I et al. *JAMA Network Open.*

Subgroup	Analysis	Metric	Arm			
			**Control**	**Simple Sweepstakes**	**Deadline Sweepstakes**	**Sweepstakes & Deposit**
Income	Below $50,000, adjusting for baseline LDL (n=414)	Individual Group	-29.8 (-37.1, -22.5)	-28.8 (-36.0, -21.7)	-26.3 (-33.7, -19.0)	-30.7 (-38.1, -23.3)
		Difference from control		1.0 (-9.3, 11.2)	3.5 (-6.9, 13.9)	-0.9 (-11.2, 9.5)
		P-value		0.855	0.512	0.871
	Above $50,000, adjusting for baseline LDL (n=380)	Individual Group	-37.4 (-44.7, -30.0)	-35.9 (-43.1, -28.7)	-39.9 (-47.0, -32.8)	-41.9 (-49.1, -34.6)
		Difference from control		1.5 (-8.7, 11.7)	-2.5 (-12.7, 7.7)	-4.5 (-14.8, 5.8)
		P-value		0.775	0.626	0.390
Baseline LDL levels	100-129 mg/l (n=419)	Individual Group	-18.2 (-24.5, -11.8)	-14.7 (-21.0, -8.4)	-15.5 (-21.4, -9.6)	-15.7 (-21.8, -9.6)
		Difference from control		3.4 (-5.3, 12.2)	2.7 (-6.0, 11.3)	2.4 (-6.3, 11.2)
		P-value		0.441	0.544	0.584
	130-159 mg/l (n=173)	Individual Group	-36.5 (-48.3, -24.7)	-21.3 (-32.9, -9.7)	-34.5 (-47.0, -21.9)	-33.4 (-44.2, -22.7)
		Difference from control		15.2 (-1.3, 31.7)	2.1 (-14.9, 19.0)	3.1 (-12.8, 19.0)
		P-value		0.070	0.812	0.703
	160-189 mg/l (n=70)	Individual Group	-36.6 (-54.8, -18.5)	-59.5 (-75.2, -43.9)	-46.4 (-63.5, -29.4)	-63.9 (-86.4, -41.4)
		Difference from control		-22.9 (-46.7, 0.9)	-9.8 (-35.1, 15.5)	-27.3 (-55.6, 1.1)
		P-value		0.060	0.448	0.060
	>190 mg/l (n=143)	Individual Group	-75.1 (-89.3, -60.8)	-84.1 (-99.0, -69.3)	-77.7 (-93.3, -62.0)	-90.4 (-106.2, -74.6)
		Difference from control		-9.1 (-29.7, 11.5)	-2.6 (-23.9, 18.7)	-15.4 (-36.8, 6.0)
		P-value		0.387	0.808	0.159
Recruitment site	Employer or Insurance, adjusting for baseline LDL (n=67)	Individual Group	-18.9 (-35.3, -2.6)	-13.4 (-27.8, 0.9)	-31.5 (-45.7, -17.4)	-20.2 (-35.6, -4.7)
		Difference from control		5.5 (-16.2, 27.3)	-12.6 (-34.2, 9.0)	-1.2 (-23.4, 21.0)
		P-value		0.618	0.252	0.915
	Penn Medicine, adjusting for baseline LDL (n=738)	Individual Group	-35.0 (-40.4, -29.5)	-34.2 (-39.6, -28.7)	-33.3 (-38.7, -27.9)	-37.9 (-43.4, -32.5)
		Difference from control		0.8 (-6.8, 8.4)	1.7 (-6.1, 9.5)	-2.9 (-10.6, 4.7)
		P-value		0.833	0.669	0.450

[a] Incomplete follow-up LDL measurements were addressed using multiple imputation unless noted otherwise

[b] Incomplete follow-up LDL measurements were NOT addressed using multiple imputation

© 2020 Barankay I et al. *JAMA Network Open.*

3.2 Maintenance of LDL-C Post-study

This analysis is based on post-hoc additions to the statistical analysis plan.

We considered whether participation in the trial succeeded in creating behaviors consistent with long-term maintenance of LDL-C levels obtained at the end of the official trial period. For 738 participants who were enrolled from the Penn Medicine, we obtained lab results from the Electronic Health Record (EHR) up to 36 months after participation in the study ended. At the time of data extraction, 498 (67.5%) participants had at least one post-study LDL measurement. 100% of the Penn Medicine participants had at least 24 months of follow-up, and 365 (49.5%) of the Penn Medicine participants had 36 months of follow-up.
As seen in eTable 5, participants who lacked any post-study measurement tended to have higher baseline LDL-C and were more likely to be black.

3.2.1 eTable 5. Penn Medicine: Baseline Characteristics of Participants With and Without a Post-Study LDL-C Measurement

A total of 399 participants had LDL-C measured both at 12-months and at least once as part of usual-care post study. Usual-care measurements were determined from the EHR up to 36 months post-study. Compared to individuals without a post-study measurement, Individuals with at least one post-study measurement were more likely to be White, have somewhat lower LDL-C at baseline, and were less likely to be from the control group. We fit a loess (locally estimated scatterplot smoothing) curve for each intervention group. eFigure 1 suggests that while there was considerable variance in LDL-C among these participants, mean LDL-C level was little changed at 2 years.

Characteristic	A least one post-study measurement (n=501)		No post-study measurement (n=237)
	With an on-study measurement at 12 months (n=399)	Without an on-study measurement at 12 months (n=102)	
Age, mean (SD)	59.7 (9.5)	58.1 (11.4)	58.3 (11.1)
Gender, n (%)			
Female	263 (65.9)	65(63.7)	155 (65.4)
Male	136 (34.1)	37 (36.3)	82 (34.6)
Race [a], n (%)			
Black	185 (46.5)	53 (52.0)	133 (56.1)
White	190 (47.7)	42 (41.2)	93 (39.2)
Other	23 (5.8)	7 (6.9)	11 (4.6)
Baseline LDL, mean (SD)	139.7 (41.6)	144.8 (37.5)	153.2 (48.3)
Intervention, n (%)			
Control	106 (26.6)	27 (26.5)	51 (21.5)
Simple Sweepstakes	98 (24.6)	24 (23.5)	61 (25.7)

© 2020 Barankay I et al. *JAMA Network Open.*

Deadline Sweepstakes	95 (23.8)	26 (25.5)	65 (27.4)
Sweepstakes & Deposit	100 (25.1)	25 (24.5)	60 (25.3)

[a] Not all participants responded; incomplete data.

3.2.2 **eFigure 1.** Penn Medicine: Change in LDL-C Post-Study With Loess Smoothed Curves

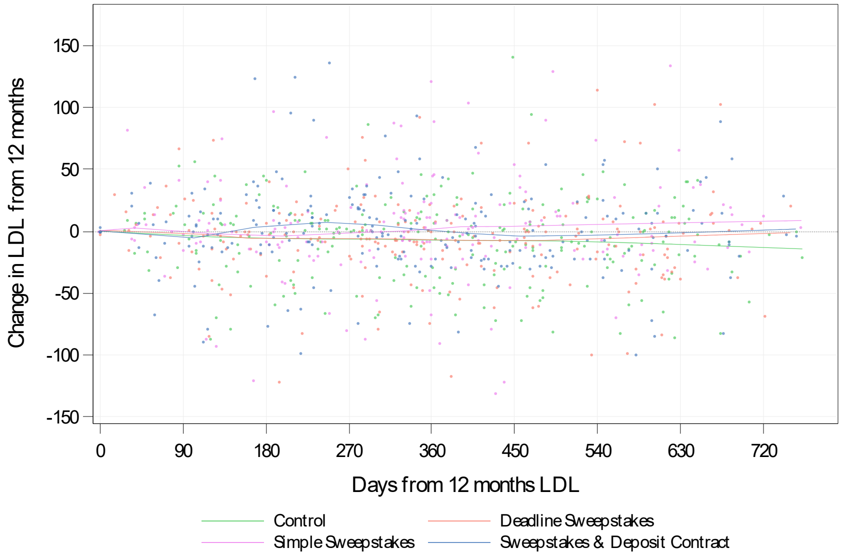

3.3 Comparison with eligible non-enrolled Penn Medicine patients.

This analysis is based on post-hoc additions to the statistical analysis plan.

Among 4404 Penn Medicine patients who were potentially eligible for the study based on an LDL-C measurement and at least one diagnostic criteria, 3666 (83.2%) did not enroll in the study. We received IRB-approval to collect information on demographics and any LDL-C value recorded in the EHR. A total of 2490 (67.9%) patients had at least one LDL-C measurement recorded in the EHR within the first year of their eligibility date.

eTable 6 suggests the non-enrolled participants tended to have somewhat lower LDL, were somewhat older and more likely to be male and white.

© 2020 Barankay I et al. *JAMA Network Open.*

3.3.1 eTable 6. Penn Medicine: Baseline Characteristics of Eligible Nonenrolled Patients Compared to Enrolled Participants in Control Group

Analyses are for participants with at least one post-baseline measurement.

Baseline Characteristic	Control (n=165)	Non-enrolled (n=2,490)
Age, mean (SD)	58.2 (10.1)	63.6 (11.2)
Gender, n (%)		
Female	111 (67.3)	1362 (54.7)
Male	54 (32.7)	1128 (45.3)
Race, n (%)		
Black	78 (47.3)	937 (37.6)
White	76 (46.1)	1320 (53.0)
Other	11 (6.7)	233 (9.4)
Missing		
Baseline LDL-C, mean (SD)	149 (46.6)	137.7 (40.9)
# Follow-ups, n (%)		
1	35 (21.2)	1360 (54.6)
2	130 (78.8)	785 (31.5)
>2	0 (0.0)	345 (13.9)

We fit a loess curve to Δ_{LDL} as a function of time for 2490 non-enrolled Penn Medicine patients with at least one post-baseline measurement. eFigure 2 suggests that LDL-C level dropped substantially in the first 90 days, and then remained reasonably constant throughout the remainder of the year.

3.3.2 eFigure 2. Penn Medicine: Nonenrolled Individuals: Change in LDL-C From Baseline With Loess Smoothing Curves

Participant had at least one post baseline measurement. Dashed line at 0 change in LDL-C.

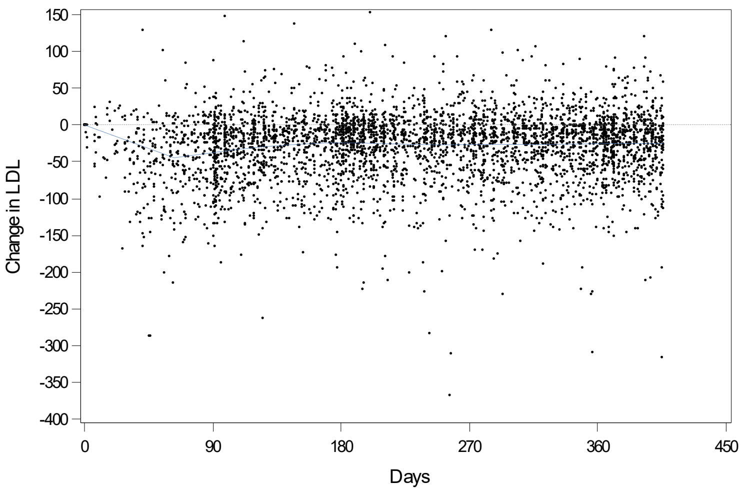

© 2020 Barankay I et al. *JAMA Network Open.*

We modeled Δ_{LDL} as a function of time using a mixed effects model with a piecewise linear spline adjusting for baseline LDL, age, gender and race as covariates in the model. This model was used to estimate the mean change from baseline at 6 and 12 months. eTable 7 includes results for the non-enrolled along with those of participants who enrolled and were randomized to the control arm.

3.3.3 eTable 7. Penn Medicine: Mean (95% CI) Change From Baseline in LDL-C at 6 and 12 Months for Participants Enrolled to the Control Arm and for Nonenrolled Individuals.

All Participants had at least one measurement post-baseline. Results are adjusted for a baseline of 143.2 mg/dl and for an individual of age 58.5 years. Results for the control arm are based on measurements collected at specific visits; results for the non-enrolled are based on the mixed effects model fit to measurements of LDL-C collected from usual-care.

Groups	Enrolled to Control Arm		Non-enrolled	
	n	Δ_{LDL}	n	Δ_{LDL}
**6 Months Endpoint (Day 180)**				
All Participants	165	-39.8 (-45.8, -33.7)	2490	-31.0 (-32.5, -29.5)
Black & Female	64	-44.3 (-51.4, -37.2)	612	-35.2 (-37.0, -33.4)
Black & Male	14	-34.2 (-47.6, -20.8)	325	-29.3 (-32.5, -26.1)
White & Female	44	-40.1 (-52.9, -27.2)	644	-35.1 (-38.2, -31.9)
White & Male	32	-39.8 (-45.8, -33.7)	676	-31.0 (-32.5, -29.5)
Other & Female	3	-37.8 (-43.7, -32.0)	106	-25.7 (-27.6, -23.8)
Other & Male	8	-43.7 (-51.6, -35.8)	127	-31.5 (-33.6, -29.4)
**12 Months Endpoint (Day 360)**				
All Participants	165	-35.1 (-40.9, -29.3)	2490	-27.8 (-29.6, -26.0)
Black & Female	32	-39.6 (-46.5, -32.7)	612	-32.0 (-34.0, -30.0)
Black & Male	3	-29.5 (-42.8, -16.2)	325	-26.1 (-29.4, -22.7)
White & Female	8	-35.4 (-48.1, -22.7)	644	-31.8 (-35.1, -28.5)
White & Male	165	-35.1 (-40.9, -29.3)	676	-27.8 (-29.6, -26.0)
Other & Male	64	-33.2 (-38.9, -27.4)	106	-22.5 (-24.6, -20.4)
Other & Female	14	-39.0 (-46.9, -31.2)	127	-28.3 (-30.6, -25.9)

© 2020 Barankay I et al. *JAMA Network Open.*

4 Adherence and Change in LDL Analysis

This analysis is based on post-hoc additions to the statistical analysis plan.

eTable 8 shows results for regression models for Δ_{LDL} at 6 and 12 months as a function of 6-month measured adherence and baseline LDL. The intervention arms were pooled for the analysis under the assumption that any effect of intervention should be mediated by adherence.

4.1 eTable 8. Regression Models for the Reduction in $\boldsymbol{\Delta_{LDL}}$ From Baseline to Either 6- or 12-Months by 6-Month Measured Adherence (Proportion of Days With Electronic Pill Bottle Opened Over 180 Days).

Outcome(mg/dl)	Predictors	Terms (95% CI)	Adjusted r^2
$\Delta_{LDL}(6\ mo)$	Intercept[1]	-11.3 (-22.8, 0.)	0.47
	Adherence (per 0.10 increase)	-2.7 (-4.0, -1.5)	
	Baseline LDL-C (per 10 mg/dl increase)	-6.9 (-6.3, -7.5)	
	Δ_{ctrl}[2]	-6.2 (-12.0, -0.4)	
$\Delta_{LDL}(12\ mo)$	Intercept[1]	-4.5 (-17.5,8.2)	0.41
	Adherence (per 0.10 increase)	-3.4 (-4.8, -2.0)	
	Baseline LDL-C (per 10 mg/dl increase)	-6.3 (-6.9, -5.7)	
	Δ_{ctrl}[2]	-5.0 (-11.3, 1.4)	
[1] Mean outcome for pooled intervention arms at the mean baseline LDL-C			
[2] Mean difference between control and intervention groups			

eFigure 3 shows boxplots for participants with measured adherence of zero. Only two individuals, both in the control arm had a measured adherence of zero for the 6-month intervention. Compared to the incentive arms, participants in the control arm with a measured adherence of zero for the final 30 days of the intervention, tended to have larger reductions in Δ_{LDL}. Some of these participants may have used devices other than their electronic pill bottle to store their medication, suggesting that measured adherence for the controls may have underestimated true adherence.

We note that temporal relationships between adherence to statin medication and LDL-C are not completely understood. Thus, it is also possible that these participants were adherent in the days or weeks preceding the final 30 days, and that this adherence was reflected in larger reductions in LDL-C.

© 2020 Barankay I et al. *JAMA Network Open.*

4.2 eFigure 3. Change in LDL-C From Baseline to 6 Months in Individuals With Measured Adherence of Zero During Either the Entire 6 Months (n=2) or the Final 30 Days of the Intervention (n=23 for Control and n=16 for Incentives).

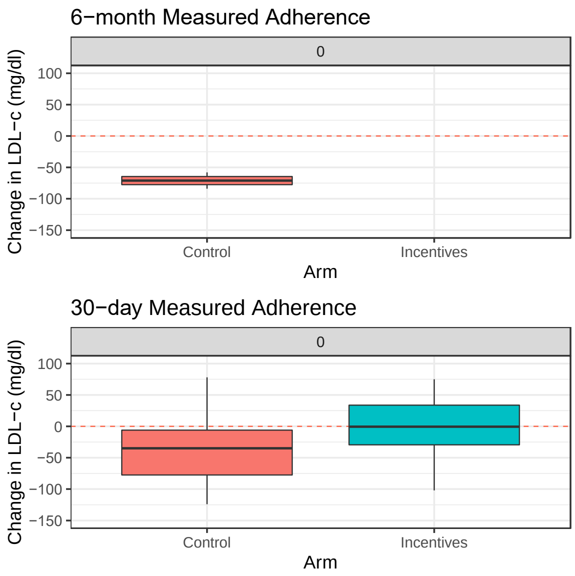

© 2020 Barankay I et al. *JAMA Network Open.*

5 Sensitivity to Failure and Model of Electronic Pill Bottles

This analysis is for additions to the statistical analysis plan specified prior to trial completion.

Among 805 participants, 360 (44.7%) were exposed to the ‘high-failure’ period. On average, participants in this trial experienced 21% of days in the intervention period during the high-failure period. Compared to individuals who were not exposed to the ‘high-failure’ period, participants exposed to the high-failure period had a 4.7 mg/dl (95% CI: -0.5, 10.0, p-value=0.07) bigger reduction in Δ_{LDL} from Baseline to 12 months, after adjusting for baseline LDL-c. We did not observe an impact of exposure to the high-failure period on the effect of the intervention (p=0.95).

A total of 51 (6.3%) of the participants experienced a device ‘swap’ i.e., these participants used at least two different models of electronic pill bottles during the trial. Among participants with at least one device swap vs those who used a single device throughout the trial, mean Δ_{LDL} from baseline to 12 months was similar (p-value=0.38). Participants exposed to the high-failure period or device swap had similar completion rates at 12 months (79.3% for participants without exposure to the high-failure period or a device swap and 78.9% for participants with exposure to the high-failure period or a device swap). We did not observe an impact of exposure to a device swap on the effect of the intervention (p=0.80).

We found significant differences in mean Δ_{LDL} from Baseline to 12 months among the three electronic pill bottle models used in the study (p=0.03). Notably, participants who only used model 3 had 9.9 (95% CI: 1.8, 18.0) mg/dl larger reduction in LDL compared with participants who only used model 1. Electronic pill bottle model did not appear to have an impact on the magnitude of the intervention on the primary outcome (p=0.61).

© 2020 Barankay I et al. *JAMA Network Open.*

6 Overall attrition rate and by arm

Note: The power calculation anticipated a 20% attrition rate (see Putt et al 2019).

Arm	Missing 12 months LDL-C, n (%)
Overall	169 (21.1)
Control	45 (22.1)
Simple Sweepstakes	40 (20.1)
Deadline Sweepstakes	42 (21.1)
Sweepstakes & Deposit Contract	42 (21.1)

© 2020 Barankay I et al. *JAMA Network Open.*
