# Supplementary material for: Effect of Patient Financial Incentives on Statin Adherence and Lipid Control: A Randomized Clinical Trial
Source: JAMA Netw Open. 2020 Oct 9;3(10):e2019429. doi: 10.1001/jamanetworkopen.2020.19429 (PMC7547367; doi:10.1001/jamanetworkopen.2020.19429)
Supplement: Supplement 3. — Data Sharing Statement [file jamanetwopen-e2019429-s003.pdf]

Data Sharing Statement

Barankay. Effect of Patient Financial Incentives on Statin Adherence and Lipid Control. *JAMA Netw Open*. Published October 09, 2020. 10.1001/jamanetworkopen.2020.19429

Data
Data available: Yes
Data types: Deidentified participant data
How to access data: barankay@upenn.edu
When available: With publication

Supporting Documents
Document types: None

Additional Information
Who can access the data: Researchers whose proposed use of the data has been approved
Types of analyses: For any reasonable purpose.
Mechanisms of data availability: After approval of a proposal.
